# Evolutionary convergence and divergence in archaeal chromosomal proteins and Chromo-like domains from bacteria and eukaryotes

**DOI:** 10.1038/s41598-018-24467-z

**Published:** 2018-04-18

**Authors:** Gurmeet Kaur, Lakshminarayan M. Iyer, Srikrishna Subramanian, L. Aravind

**Affiliations:** 1 National Center for Biotechnology Information, National Library of Medicine, National Institutes of Health, Bethesda, MD 20894 USA; 2 Bioinformatics Centre, CSIR-Institute of Microbial Technology, Sector 39A, Chandigarh, 160036 India

## Abstract

SH3-fold-β-barrel domains of the chromo-like superfamily recognize epigenetic marks in eukaryotic proteins. Their provenance has been placed either in archaea, based on apparent structural similarity to chromatin-compacting Sul7d and Cren7 proteins, or in bacteria based on the presence of sequence homologs. Using sequence and structural evidence we establish that the archaeal Cren7/Sul7 proteins emerged from a zinc ribbon (ZnR) ancestor. Further, we show that the ancestral eukaryotic chromo-like domains evolved from bacterial versions, likely acquired from early endosymbioses, which already possessed an aromatic cage for recognition of modified amino-groups. These bacterial versions are part of a radiation of secreted SH3-fold domains, which spawned both chromo-like domains and classical SH3 domains in the context of peptide-recognition in the peptidoglycan or the extracellular matrix. This establishes that Cren7/Sul7 converged to a “SH3”-like state from a ZnR precursor via the loss of metal-chelation and acquisition of stronger hydrophobic interactions; it is unlikely to have participated in the evolution of the chromo-like domains. We show that archaea possess several Cren7/Sul7-related proteins with intact Zn-chelating ligands, which we predict to play previously unstudied roles in chromosome segregation during cell-division comparable to the PRC barrel and CdvA domain proteins.

## Introduction

Three-dimensional structures or folds of proteins are evolutionarily less prone to change than their sequences^1–3^. In the absence of statistically-significant sequence similarity, the detection of structural equivalences can be used to assess evolutionary relatedness^1,4,5^. However, the evidence can, in some instances, be equivocal regarding structural convergence versus divergence: the moot point in these cases is whether the structural similarity in folds in question is a signal of a divergent origin from a common ancestor or independent convergence to a common scaffold^1,6–9^. Automated sequence- and structure-similarity search tools, though widely used for gauging relatedness among proteins, are often of limited utility in these cases. Tracing the correct relationships demands careful case-by-case analysis^5,10^ and on multiple occasions, has helped untangle convergence from extreme divergence, which had otherwise eluded automated similarity search tools^11–17^. In this work, we present such a case regarding the SH3 fold and certain zinc ribbons (ZnRs), with bearing on the function and evolution of key domains involved in chromatin structure and chromosome segregation in archaea, recognition of epigenetic marks in eukaryotes, and bacterial cell-wall dynamics.

The Src homology 3 (SH3) is a small β-barrel domain, comprised of five or six β-strands that are tightly packed into two orthogonal β-sheets^18^. The eponymous SH3 domains are involved in eukaryotic signaling pathways where they mediate protein-protein interactions by binding proline-rich peptide sequences via a conserved cluster of aromatic residues^18–20^. The discovery of bacterial homologs of the SH3 domain presented an interesting contrast as they were primarily found as extracellular domains in periplasmic or cell-wall associated proteins^21–23^. Members of the larger SH3-like β-barrel fold (hereinafter SH3 fold) include a vast collection of superfamilies found in diverse biological functional contexts. The best characterized of them are implicated in a variety of key protein-protein interactions via recognition of short peptide motifs^24^. SH3-fold β-barrels also mediate interactions with nucleic acids^24,25^. For example, PAZ (Piwi Argonaut and Zwille), a SH3-fold β-barrel domain, found in the Piwi and the Dicer proteins in the RNAi system interacts with RNA^26–28^. Likewise, certain representatives of other families with the SH3 fold such as the CarD^29,30^, chromo^31^, and TUDOR domains^32,33^ have been shown to bind DNA.

In recent years, it has become clear that in eukaryotes a large superfamily of domains with the SH3 fold, the chromo-like superfamily, plays a key role in recognition of short peptide-motifs, especially those with covalently modified side-chains in chromatin (chiefly histones) and RNA-processing proteins. This superfamily includes the classical chromo (chromatin organization modifier) domains, BAM/BAH, BMB/PWWP and Tudor-like domains^34–36^. The Tudor-like domains further include within them the Tudor, MBT (malignant brain tumor), Agenet, DUF3590, DUF1325, RAD53BP, Tudor-knot, AuxRF(PF06507) and the MORC C-terminal domains^37,38^ (PFAM clan CL0049). The conserved structural core of the chromo-like domains features a SH3-fold β-barrel with 5 strands that is often capped by a C-terminal helix^34,35^. They share a broadly conserved mode of interaction with peptides, specifically recognizing covalent modifications of positively charged side-chains via cation-π interactions with conserved aromatic residues^39^. While most members bind peptides with methylated lysines, representatives of the Tudor-like domains specialize in binding peptides with methylated arginines.

These covalent modifications along with the chromo-like domains that bind them are defining features of all eukaryotes, which set them apart from prokaryotes^37^. Hence, understanding the provenance of eukaryotes depends on a proper explanation for their origins in the stem eukaryotic lineage. Sequence and structure comparison studies have proposed two distinct possibilities for their origins. In the first, based on structural similarity, an evolutionary relationship was proposed between the eukaryotic chromo-like domains and the SH3-like fold of archaeal Sul7d-like chromatin-compaction proteins^40^. These in turn are structurally and functionally related to the major pan-crenarchaeal Cren7 protein, also involved in DNA-compaction and supercoiling^41,42^. Thus, in this scenario, the eukaryotic chromo-like domains were derived from an archaeal chromatin protein in the context of chromatin function^40^. In the alternative scenario, the presence of unambiguous sequence homologs of the chromo-like domains in bacterial proteins suggest that the eukaryotic versions evolved from the bacterial precursors^38^.

To distinguish between these alternatives and better understand the function of the bacterial chromo-like domains we sought to utilize the wealth of new genomic and structural data. Using sequence and structure comparisons, we demonstrate that the Cren7/Sul7-like “SH3 fold” domains are members of the zinc ribbon fold with certain members, including Cren7, secondarily losing their ability to chelate a zinc ion. We further shown that the Sul7 proteins most likely arose in Sulfolobales as a paralogous family of Cren7. We also show that the eukaryotic chromo-like domains instead evolved from bacterial precursors as part of the radiation of extracellular SH3 fold domains in the context of bacterial cell-wall dynamics. Based on these considerations, we hypothesize that the SH3-like β-barrel architecture convergently emerged from an ancestral ZnR fold in Cren7/Sul7-like proteins.

## Results and Discussion

### Structural diversity of ZnR domains and the relationship of certain versions to the SH3-like β-barrel fold

**Figure 1:**
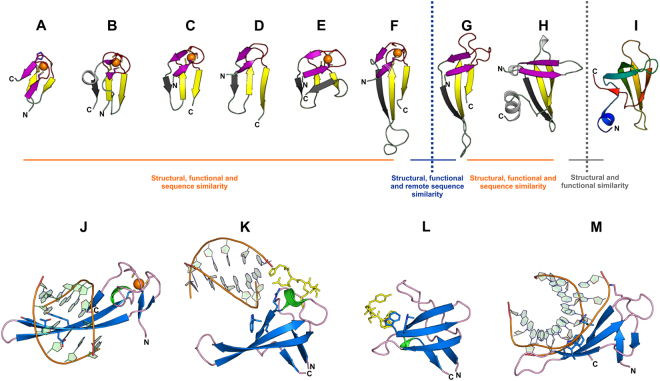
Comparative structural view of the major secondary elements (**A**–**I**) and binding interfaces (**J–M**) in ZnRs, SH3s and Cren7. (**A**) Type-1A ZnR (PR domain-containing protein 11, PDB: 3RAY_A) (**B**) Type-1B zinc ribbon (Rubrerythrin, PDB: 1NNQ_A) (**C**) Type-2 zinc ribbon (Archaeal exosome RNA binding protein CSL4, PDB: 2BA1_A) (**D**) Type-2 zinc ribbon (Uncharacterized BCR *cg1592*, PDB: 2JNY_A) (**E**) ZnRs with three strands on each side (Anaerobic ribonucleoside-triphosphate reductase, PDB: 1H8K_A) (**F**) Type-2 zinc finger (Transcriptional Elongation Factor SII, PDB: 1TFI_A) (**G**) Cren7 (PDB: 3KXT_A) (**H**) Sso7 (PDB: 1C8C_A) (**I**) SH3-like fold (Tudor domain-containing protein 3, PDB: 3PNW_O). (**J**) DNA bound zinc ribbon domain (putative integrase [Bacteriophage A118], PDB: 4KIS_A) (**K**) DNA and peptide bound chromodomain (Male specific Lethal-3, PDB: 3OA6_A) (**L**) peptide bound SH3 domain (Tyrosine-protein kinase ABL1, PDB: 1BBZ_A) (**M**) DNA bound Cren7 (PDB: 3KXT_A). In all the panels, the Zn ion is represented by an orange sphere, side chains of zinc-chelating and other functionally-important amino acids are represented as sticks, bound peptides are yellow colored stick, the sugar-phosphate backbone of DNA is orange with nucleotides in green. Color scheme for (**A**–**I**): For type-2 zinc ribbons, the N-terminal β-hairpin is colored purple, the third β-strand is colored gray, the zinc knuckles are colored red and the C-terminal β-hairpin is colored yellow. The equivalent β-strands in type-2, -1A and -1B are colored alike. The Tudor domain is colored in a gradient of blue to red from the N- to the C-terminal. In panels (**J**–**M**): All β-strands are colored in blue, all loops in pink and α-helices in green.

ZnRs are small domains that lack an extensive hydrophobic core beyond the presence of two β-hairpins, being primarily stabilized by chelation of a metal ion^43–46^. The metal ion is chelated by two cysteine ligands from each of the “knuckles” with the consensus motif ‘CPxCG’ situated in the turn of each β-hairpin^45–47^. A comparative structural analysis revealed two major types of the ZnR fold, type-1 and type-2. The type-1 ZnRs have a distinct separation between the N-terminal and C-terminal β-hairpins such that no contiguous β-sheets incorporating the two knuckles or parts thereof are observed. Type-1 ZnRs can be further distinguished into two sub-types based on the presence of an additional β-strand at the C-terminus. Type-1A ZnRs (Fig. 1A) have a structural core made up of only two β-hairpins. Examples of these ZnRs include the Ran-binding family (PDB: 3CH5_B), the pre-SET domain associated with certain SET domain protein methylases (PDB: 2L9Z_A) and cytochrome-c oxidase polypeptide Vb (PDB: 1OCC_F). In Type-1B ZnRs, (Fig. 1B), the C-terminal β-hairpin extends into an additional β-strand that forms a three-stranded β-sheet with the N-terminal β-hairpin. The type-1B ZnRs are typified by the rubredoxins (PDB: 1DX8), the ZnRs of methionyl tRNA synthetases (PDB: 1A8H), class IIA glycyl tRNA synthetases (PDB: 2ZT5)^48^ and threonine synthase (PDB: 1VB3)^49^, and the ubiquitin-binding ZnRs of deubiquitinating peptidases of the UBCH family (PDB: 2GFO).

In type-2 ZnRs the N-terminal β-hairpin is extended into a β-strand that forms hydrogen bonds with the C-terminal β-hairpin resulting in a sheet formed by a three-stranded β-meander (Fig. 1C–F). Type-2 ZnRs tend to be prevalent in nucleic acid-binding proteins (PDB: 1TFI, 1L1O) and in a strand-swapped version in the DNA-binding Ku and MarR family ZnR domains (PDB: 1JEY, 2F2E)^46,50,51^. Despite these structural differences the two classes of ZnRs are likely to be related because they possess a common four-stranded core, conserved geometry of the Zn-chelating residues and can be structurally superimposed.

Interestingly, while developing this classification of ZnRs, we noticed a consistent structural similarity between type-2 ZnRs and SH3 domains **(**Fig. 1G–I**)**. For example, a DALI^52^ search initiated with the ZnRs from the RNA polymerase subunit RBP9 (PDB: 1QYP_A), in addition to retrieving various ZnRs (e.g. Ribonuclease P protein component 4, PDB: 1X0T_A, Z-score = 3.9, RMSD = 1.2 Å, lali = 38; zinc finger protein ZPR1, PDB: 2QKD_A, Z-score = 3.3, RMSD = 3.3 Å, lali = 40), recovered several distinct SH3-like β-barrel domains albeit with lower Z-scores such as the classic SH3 domains (e.g. Myosin VI, PDB: 2VB6_A, Z-score = 2.2, RMSD = 1.8 Å, lali = 33; Rho guanine exchange factor 16, PDB: 1X6B, Z-score = 2.2, RMSD = 5.1 Å, lali = 43), the Tudor domain (e.g. Survival motor neuron protein, PDB: 4A4E_A, Z-score = 2.5, RMSD = 2.9 Å, lali = 41) and the chromo domain (e.g. Mortality factor 4-like protein 1, PDB: 2F5K_F, Z-score = 2.0, RMSD = 2.6 Å, lali = 39). Several SH3-like β-barrel folds were also retrieved in structural searches initiated with the ZnRs of peptide:N-glycanase (PDB: 1X3W_A), ORF131 of *Pyrobaculum* spherical virus (PDB: 2X5C_A), anaerobic ribonucleotide-triphosphate reductase (PDB: 1HK8_A) and lysyl-tRNA synthetase (PDB: 1IRX_A). Further, visual inspection confirmed this relationship, indicating a topological similarity in arrangement of the β-strands in type-2 ZnRs and the β-barrel of certain domains with the SH3 fold **(**Fig. 1E–I**)**.

### Type-2 ZnRs and SH3-fold domains show comparable ligand-binding interfaces

The detection of this relationship between type-2 ZnRs and SH3 fold domains led us to further compare the ligand-binding interfaces of members of the two folds. In type-2 ZnRs the ligand-binding surface is formed by the three-stranded β-sheet typical of these domains^45^. The interacting residues often emanate from strands β3 and β4 and frequently have aromatic or charged side chains (Fig. 1J). For example, such an interface is used by ZnRs in TFIIS, TBP N-terminal domain, Ku, RBP9 and the RNaseP Rpp21 to contact nucleic acids^53–55^. Interestingly, while chromo-like domains bind methylated peptides mainly via the open region of the barrel, representatives bind nucleic-acids using an interface that is spatially comparable to the equivalent interface of the Type-2 ZnRs. (Fig. 1J,K). Classical SH3 domains use the same interface as the nucleic-acid-binding chromo-like domains, with a conserved tryptophan residue, to bind their proline-rich peptide ligand^19,20^ (Fig. 1L). The position of this tryptophan corresponds to that of the nucleic acid-interacting residues in ZnRs. These observations suggest that in addition to the structural similarities, at least one binding interface of the SH3 fold domains is similar that of the type-2 ZnRs. This analysis also led us to the archaeal chromosomal proteins Cren7 (PDB: 3KXT; Fig. 1G) and Sac7d/Sul7 (PDB: 1WD0; Fig. 1H) proteins, which have been classified with the chromo-like SH3 fold domains^3,56^, and have even been proposed as their precursors^40^. In these proteins too, the residues responsible for DNA-binding are mainly contributed by the region of the triple-stranded β-sheet (β3-β4-β5) (Fig. 1M)^41,57,58^ which presents a clear parallel to the type-2 ZnRs.

### The origin of Cren7 and Sul7 proteins from ZnRs and their diversification in archaea

The above observations hinted that Cren7 with structural features resembling both the SH3 fold and ZnR domains might help better understand the connections between the two folds. Consistent with earlier findings^41^, DALI searches initiated with Cren7 protein (PDB: 3KXT_A), recovered multiple SH3-like fold domains such as chromo domains, Tudor domains and myosin S1 fragment SH3 domains^41^. These searches also recovered the Sul7 protein^41,42^ (PDB: 1WVL_A), which is believed to be structurally and functionally related to Cren7 as one of the hits (PDB: 1WVL_A, Z-score: 3.3, RMSD = 3.1 Å lali = 46). Concurrently, the search also retrieved hits to ZnRs, such as those in the sarcosine oxidase delta subunit (PDB: 1VRQ_D, Z-score = 3.0, RMSD = 2.8 Å, lali = 44), PNGase (PDB: 3ESW_A, Z-score = 2.8, RMSD = 2.6 Å, lali = 42), ZPR1 (PDB: 2QKD_A, Z-score = 2.4, RMSD = 3.4 Å, lali = 44), peptide:N-glycanase (PDB: 1 × 3W_A, Z-score = 2.2, RMSD = 3.1 Å, lali = 43) and 50 S ribosomal protein L44E (PDB: 1Q81_4, Z-score = 2.0, RMSD = 3.2 Å, lali = 45). Manual structural superimposition of Cren7 (PDB: 3KXT_A) and ZnRs (eg. sarcosine oxidase delta subunit, PDB: 1VRQ_D) perfectly aligned all secondary structure elements of the Cren7 β-barrel onto the core of the ZnR fold (RMSD = 1.5 Å over 35 pairs of backbone C_α_ atoms), where the turns of β1/β2 and of β4/β5 of Cren7 recapitulate the position of knuckles in ZnRs. Similar results were obtained by automated pairwise structural alignment of Cren7 (PDB: 3KXT_A) and ZnRs (e.g. sarcosine oxidase delta subunit, PDB: 1VRQ_D) using TM-align^59^ and Fr-TM-align^60^ which gave a TM score of 0.52 (normalised by the length of 3KXT_A), indicating a ‘fold level’ similarity between the two.

**Figure 2:**
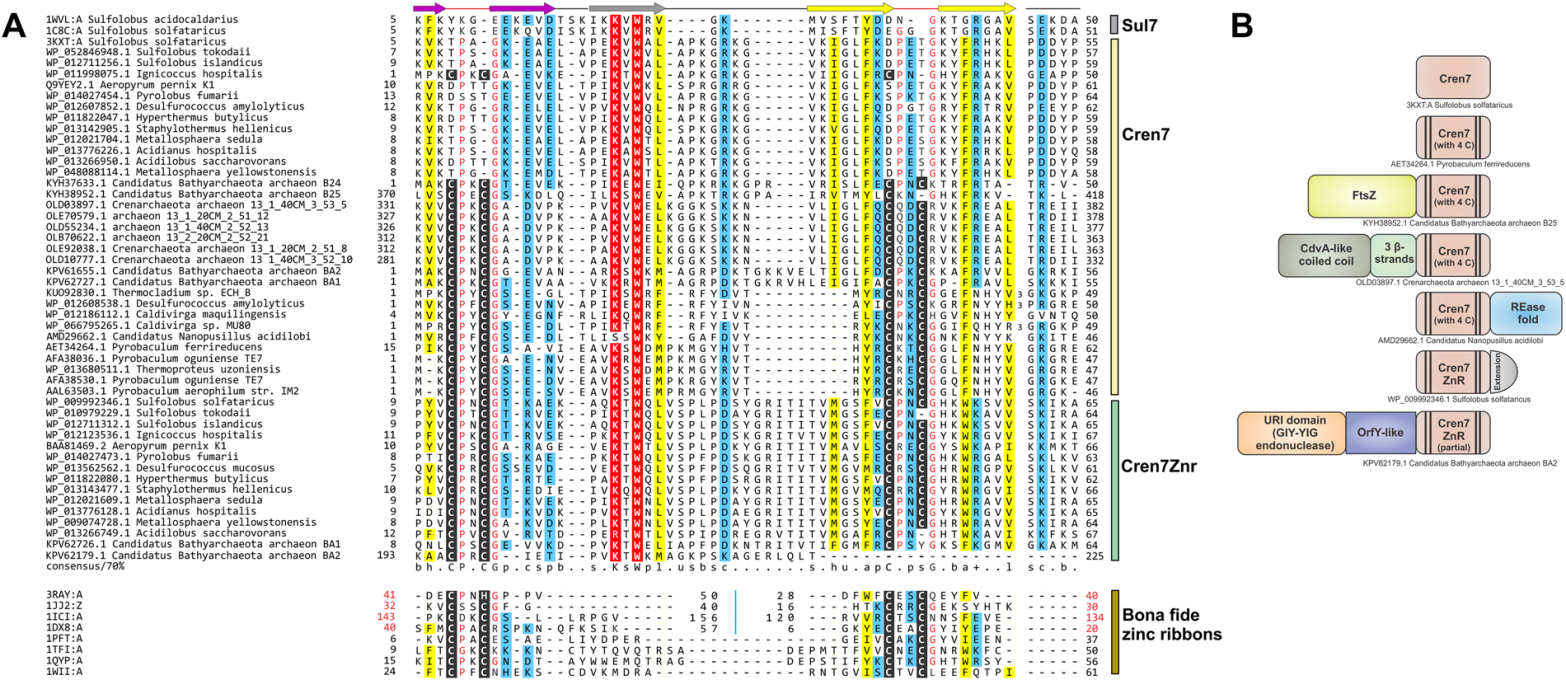
Multiple sequence alignment (MSA) and domain architectures of Cren7 proteins. (**A**) Structure-based MSA of representative sequences of Sul7, Cren7, Cren7Znr and bona fide ZnRs. Accession number or the PDB identifier, and the alignment range are indicated for each sequence. For Sul7, Cren7 and Cren7Znr proteins, the organism names are also mentioned after the accession number/PDB. Zn-chelating residues are boxed in black and small amino acids (Gly, Pro) just after the Zn-binding ligands are colored in red. At positions with a 70% consensus, charged or polar residues are highlighted in blue, and hydrophobic, apolar and aliphatic residues are in yellow. Some insertions are not displayed for clarity, and the number of omitted residues is indicated. Regions of circular permutation in ZnRs are separated by a blue ‘|’ mark and the sequence numbers of the permuted ZnRs are shown in red. The consensus secondary structures are depicted above the alignment. (**B**) Domain architectures of Cren7 and Cren7Znr proteins. Representative sequence identifier with organism name are mention below individual architectures.

To investigate if this structural relationship to ZnRs also extends to sequence similarity, we initiated iterative sequence similarity searches with *S. solfataricus* Cren7 (PDB: 3KXT_A) using the PSI-BLAST^61^ and the JACKHMMER^62^ programs. Interestingly, these searches recovered multiple orthologous sequences from crenarchaeota and bathyarchaeota that contained two to four cysteine residues at positions corresponding to turns between strands β1/β2 and β4/β5 (eg. *Ignicoccus hospitalis*, WP_011998075.1) (Fig. 2A). The positions of these cysteines suggest that when four of them are present they are likely to chelate a Zn ion. Moreover, we found another group of Cren7 homologs from several crenarchaea which contained a Cren7 domain as part of a larger multidomain protein (see below). These Cren7 domains were characterized by the presence of all four expected Zn-chelating cysteines. Additionally, our searches also retrieved a distinct paralogous family of crenarchaeal proteins (Cren7Znr family; e.g. APE_2454.1 from *Aeropyrum pernix*; BAA81469.2) typified by an extended C-terminal region, most of whose members possess intact Zn-chelating cysteines (Fig. 2A). Profile-profile sequence similarity searches^63^ initiated with the Cren7 and Cren7ZnR domains consistently retrieved ZnRs. For example, HHPRED searches with *I. hospitalis* (WP_011998075.1) found matches to many ZnR proteins such as  the lysine biosynthesis protein LysX/ArgZ (PDB: 5K2M_E, E-value = 0.0005), ZitP pilus assembly/motility regulator (PDB: 2NB9_A, E-value = 0.0054) and transcriptional regulator MqsA (PDB: 3O9X_A, E-value = 0.091). Further, even among the Cren7Znr proteins some representatives show loss of one of the Zn-chelating cysteines exactly paralleling the situation observed among the classic Cren7 proteins (Fig. 2A).

Thus, multiple lines of evidence support Cren7-like proteins (PDB: 3KXT_A) being ZnRs, with secondary loss of Zn-chelating residues in some versions such as the *S. solfataricus* Cren7. Although the Sul7 proteins were not retrieved in these sequence searches, they show several features that suggests their derivation from Cren7 proteins. (1) They are so far only found in a limited number of crenarchaeal lineages (*Sulfolobus*, *Acidanus* and *Metallosphaera)* unlike Cren7, which is conserved across crenarchaeota and the bathyarchaeota^64^. (2) They share a common DNA-compaction function in crenarchaeal chromatin^41,42,58^, which is mediated via a similar protein-DNA interface, with positionally- and chemically- equivalent residues involved in DNA contact. (3) Experiments to make the Cren7 protein more “Sul7d-like” by mutating the loop between strands β3/β4 results in an exact superposition of the DNA-binding interface^41,42,58^.

Presence of Cren7/Sul7 proteins only in crenarchaeota and bathyarchaeota, together with their provenance from ZnRs, which we establish above supports the following evolutionary scenario: the ancestral Cren7 proteins likely arose from a DNA-binding ZnR of which several are found in the pan-archaeo-eukaryotic transcription apparatus^46^. Consistent with this, we have found versions of Cren7, which still retain the ancestral Zn-chelating residues. The strengthening of the hydrophobic core due to interactions in the strand regions appear to have facilitated the loss of Zn-chelation in more than one version of the family. This appears to have concomitantly supported the emergence of a more β-barrel-like geometry that converged to a SH3-like state. This was followed by the emergence of Sul7 only in Sulfolobaceae as a specialized DNA-packaging protein from a Cren7-like protein that had already lost its Zn-chelating residues.

Our recovery of novel members of the Cren7-like family suggests that they underwent functional diversification in the archaeal lineages that possess them. Notably, in one group of these proteins, Cren7 is the C-terminal domain of much larger protein (e.g. OLD03897.1) where it is combined with an N-terminal CdvA-like coiled coil domain and a central small 3-stranded domain. In crenarchaea, the CdvA-like coiled-coil domain proteins are components of the cell-division system and form filamentous double-helical complexes with DNA^65^. In bathyarchaea, we found a fusion of the Cren7 domain to an N-terminal FtsZ domain (KYH38952.1), a key component of the cell-division apparatus related to the tubulin-like cytoskeletal proteins. These architectures suggest that representatives of the Cren7 family play a specific role in cell-division, probably in anchoring the DNA. All Cren7Znr proteins contain a conserved C-terminal motif with an absolutely conserved acidic residue beyond the core Cren7 domain, which is likely to adopt an extended conformation. It is possible that this region also helps in specific interactions with cell-division components. We also found an instance of bathyarchaeal Cren7 (KPV62179.1) fused to the URI domain (GIY-YIG endonuclease) and an uncharacterized enzymatic domain of the OrfY-like superfamily^66^, and a nanoarchaeal Cren7 (AMD29662.1) with a C-terminal REase fold nuclease domain (Fig. 2B; Supplementary Material S1, S2).

### Bacterial diversification of chromo-like SH3 fold domains

**Figure 3:**
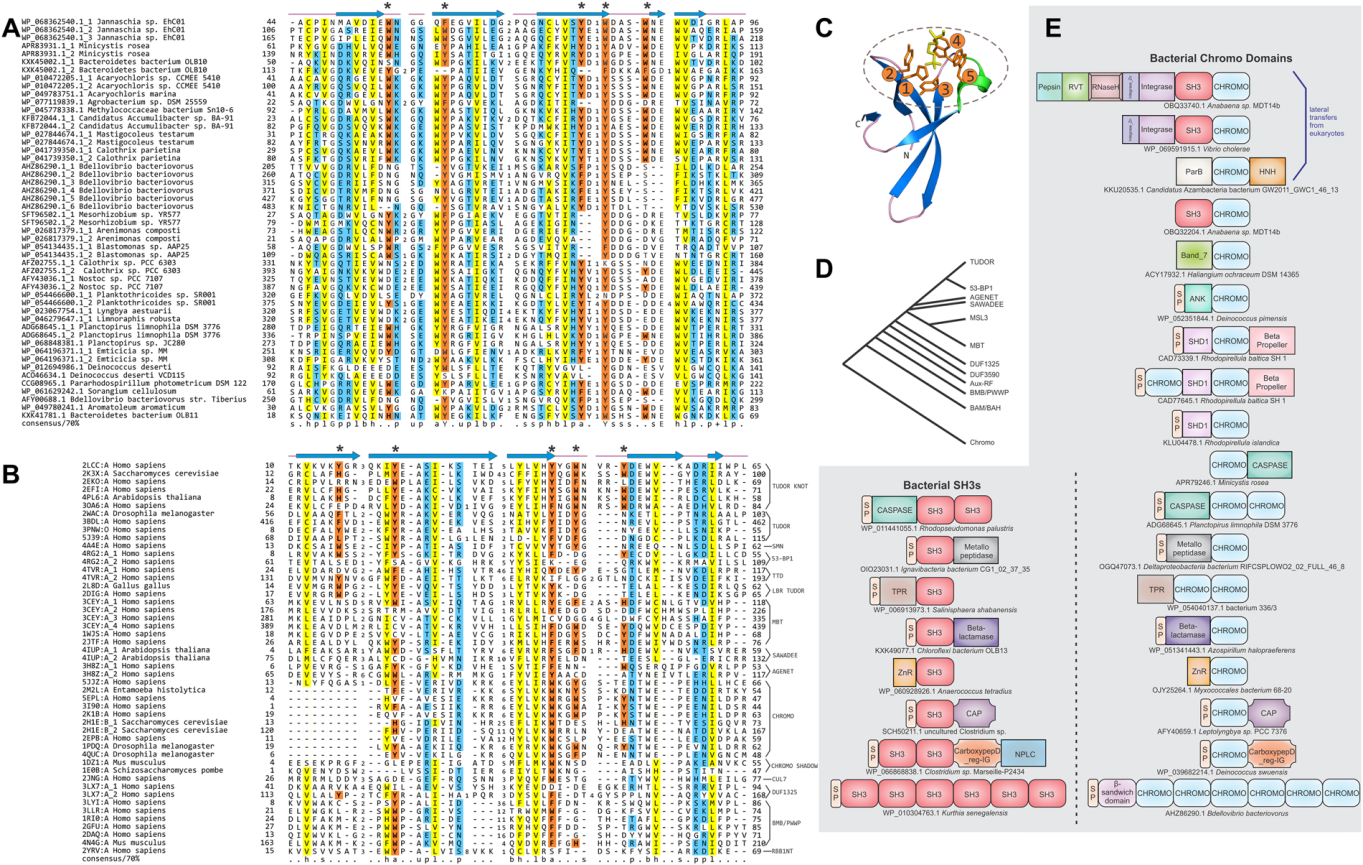
Multiple sequence alignment (MSA), probable phylogeny and domain architectures of chromo-like domains. (**A**) MSA of representative bacterial chromo-like domains. (**B**) Structure-based MSA of representative eukaryotic chromo-like domains. Coloring scheme for panel (A) and (B) follows Fig. 2(A). The amino acids at positions that are likely to be involved in forming the peptide-binding aromatic cage are highlighted in orange and marked with the asterisk above the MSA. (**C**) View of the peptide-binding aromatic cage of *Arabidopsis* Morf Related Gene (MRG) group protein MRG2 chromo domain (PDB: 4PL6). The aromatic cage is marked by a dashed ellipse and the aromatic residue positions from the N- to C- terminus are marked by numbers 1–5. (**D**) Probable phylogenetic relationships between the various chromo-like domains. (**E**) Domain architectures of bacterial chromo domains. Parallel architectures among bacterial chromo and bacterial SH3 domains are grouped at the bottom. SP indicates signal peptide. Besides the architectures shown in the figure, there are various other bacteria proteins with 2 to 14 tandem SH3 domains and 2 to 6 tandem chromo domains. (see Supplementary Material S3 for details).

**Figure 4:**
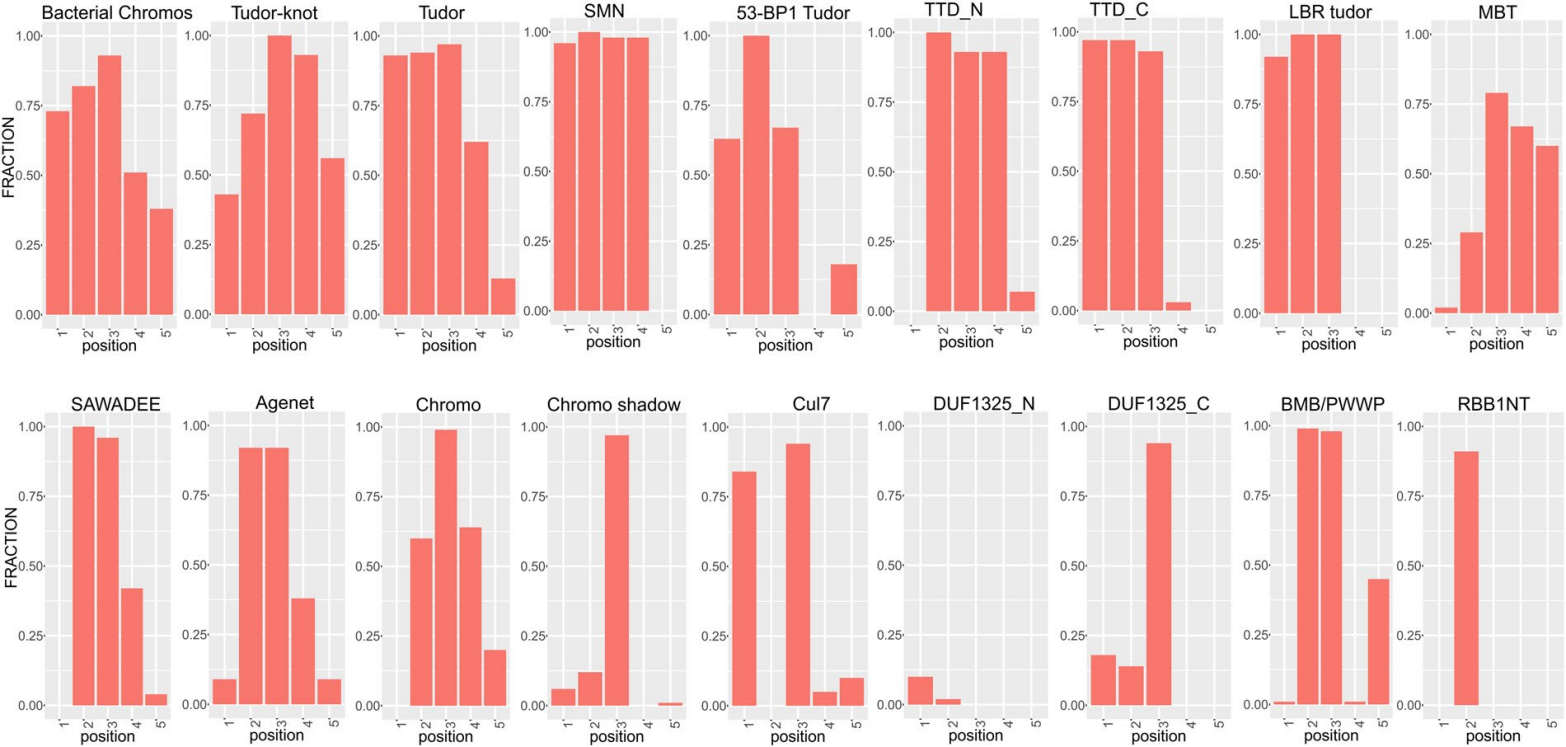
Comparative graphical view of the conservation of aromatic residues at the five positions that make up the peptide-binding aromatic cage in chromo-like domains. Individual graphs are labelled on the top by the family they represent. The X-axis represents positions corresponding to those shown in Fig. 3(C). The Y-axis represents the fraction of aromatic amino acids (Y, W, F, H) at those positions in the respective families.

The above rooting of the provenance of Cren7/Sul7-like proteins within the archaeal radiation of ZnRs and evidence for convergent acquisition of the SH3-like barrel morphology questions the evolutionary relationship of these archaeal chromosomal proteins and the chromo-like superfamily of SH3 fold, given the previous identification of bacterial versions of the latter^38^. To better understand the origins of the bacterial chromo-like domains, we ran iterative profile searches from the versions we had previously detected in bacteria^38^. Our current searches, greatly extended the phyletic spread of bacterial chromo-like domains, retrieving them from several different lineages such as proteobacteria (mainly α and δ), cyanobacteria, Thermus/Deinococcus, bacteroidetes, planctomycetes, and spirochaetes and in rare cases in euryarchaea. Firmicutes and actinobacteria showed a strong under-representation of this domain (see Supplementary Material S3). A multiple sequence alignment of the bacterial chromo-like domains with various eukaryotic versions revealed that the bacterial homologs strongly conserve at least a subset of the aromatic residues corresponding to the aromatic cage involved in ligand-binding (Fig. 3A,B)^38,67,68^. Given that these residues are central to the recognition of methylated ε-amino groups of lysine in the bound peptide in eukaryotic chromo-like domains (Fig. 3C), we posit a similar binding capacity for the bacterial versions. Across eukaryotic chromo-like domains, the Tudor assemblage strongly conserves the residues forming the aromatic cage, whereas the classical chromo domains show some variability in these residues **(**Fig. 4**)**. This suggests that the bacterial versions are closer to the Tudor-like chromo domains and that the Tudor-like versions are likely to be closer to the ancestral mode of binding peptides. The plausible evolutionary relationship shared by the various chromo-like domains is depicted in Fig. 3D.

We observed that the bacterial chromo-like domains, in contrast to their eukaryotic counterparts, are consistently present in secreted proteins across the diverse lineages containing them. Some proteins with bacterial chromo-like domains also display cysteines likely to form disulphide bonds typical of extracellular proteins. Further, systematic analysis of their domain architectures revealed contexts unlike any seen in eukaryotes (Fig. 3E): in addition to being found in tandem repeats (2–6 domains per protein), one of the most common linkages of the secreted bacterial chromo-like domains is with the caspase-like peptidase domain. This architecture is found in plantomycetes, cyanobacteria, bacteriodetes and chloroflexi. Notably, less-frequent but parallel fusions are also observed with other extracellular peptide-bond hydrolase domains namely a zincin-like metallopeptidase and a β-lactamase domain (Fig. 3E). Additionally, these chromo-like domains are also combined in extracellular proteins with several other non-catalytic domains, such as another SH3-fold domain the Slap homology domain 1 (SHD1), WD40-like β-propeller domains, the EF-hand, the Ig-fold carboxypeptidase regulatory domain and TPR repeats. Interestingly, parallel domain architectures with multiple tandem domains and fusions to the caspase, zincin-like metallopeptidase, β-lactamase, WD40 β-propellers, the carboxypeptidase regulatory and TPR repeat domains are seen for bacterial representatives of classical SH3 domains (Fig. 3B)^21,23^, again in contrast to their strictly intracellular location of the eukaryotic counterparts^19^. These bacterial SH3 domain proteins are distributed across a much wider phyletic range of taxa compared to the chromo-like domains. Recent studies have shown that secreted bacterial SH3 domains are likely involved in binding peptides in the peptidoglycan cell wall^21–23^. These parallels suggest that the bacterial chromo-like domains might function similarly to the bacterial SH3 in binding-specific peptides. However, they are likely to possess specificity for those containing methylated lysine-like moieties, in the murein or extracellular matrix peptides in bacteria with Gram-negative cell walls (given their near absence in firmicutes and actinobacteria).

In addition to the above architectures, we also observed two unusual lateral transfers of the eukaryotic chromo domains to bacteria: (1) a Ty-3 family retrotransposon, which is commonly found in fungi is also found in multiple copies in *Anabaena* (for example, Accession no. OBQ33740.1, from *Anabaena* sp. MDT14b). Here the chromo domain is fused C-terminal to the polyprotein containing pepsin, reverse transcriptase, RNase H, integrase and SH3 domains. Given the association of certain fungi with cyanobacteria (e.g. example, in cyanolichens) this probably represents a transfer facilitated by such an association. (2) The other case is found in a single species, where a eukaryotic chromodomain is inserted into a mobile element of a bacterium with a ParB and HNH domains (accession: KKU20535.1, *Azambacteria bacterium* GW2011_GWC1_46_13) (see Supplementary Material S3).

## Conclusions

Small metal-chelating domains, such as ZnRs, likely originated with a relatively simple stabilizing core in the form of the Zn-chelating center. The ancestral ZnRs themselves could have emerged from a pair of small metal-stabilized, bi-cysteine, knuckle-like motifs that existed independently (similar to the minimal versions seen, for example, in Rad50 zinc-hook, PDB: 1L8D and the RNase E zinc-link domain, PDB: 2VMK). More structured ZnRs with β-hairpins developing around these Zn-knuckles at their turns (Fig. 1A–F) likely evolved from such versions and acquired the ability to exist as independent domains. These simplest versions of ZnRs might have resembled the type-1A scaffold as they have separate β-hairpins with the two metal chelating residue pairs (Fig. 1A). This provided a platform for considerable evolutionary variability and structural innovation with augmentation and/or supplanting of the original Zn-center via the emergence of further stabilizing hydrogen-bonding networks and hydrophobic cores in the form of new, more ordered secondary structure elements^9,69–71^. Such developments are seen in the form of the type-1B ZnRs, which developed an additional β-strand and finally the type-2 ZnRs, which appear to be related to the type-1B versions via a circular permutation. Here, the additional β-strand appears to have been further incorporated into the core of the fold to form a contiguous β-sheet (Fig. 1C–F). Indeed, loss of metal-ion chelation in such domains could accompany the development of alternative stabilizing hydrophobic cores, which might then be the progenitor of a distinct protein fold^50,72,73^. This could then be subject to further modifications via duplications and/or circular permutations^45,74–77^.

In this study, we capture the evolutionary stages in one such transformation: the Cren7 domain, previously considered a SH3 fold β-barrel, actually emerged from a metal-binding ZnR domain. We find evidence that such convergence between ZnRs and SH3 fold domains might have happened independently more than once. For example, the segment-swapped Ku-bridge domain and its homolog: the C-terminal all-β domain of the MarR-like transcription factors resemble segment-swapped SH3-like folds (labelled as SH3-like in SCOP and SCOP2; SCOP identifier 140307); however, sequence analysis clearly indicated the provenance of these domains from type-2 ZnRs^50,51^. We also observed that the type-2 ZnR at C-terminus of the nicotinate phosphoribosyltransferase converged to a SH3-like fold with the Zn-chelating sites lost alongside the evolution of compensatory hydrophobic interactions (Supplementary Figure S1). Further, this convergence can also extend to the substrate-binding mode of certain ZnRs and the SH3 fold domains (Fig. 1J–M). This raises the possibility that similar transitions from a ZnR might have spawned SH3-like fold domains on other occasions too but cannot be currently confirmed using sequence-based methods. Notably, both ZnRs and SH3 fold domains are found in proteins highly conserved across life, such as the ribosomal subunits^78^. Hence, it is possible that ancient representatives of the two folds also share an ultimately divergent relationship in a very early period of the evolution of protein universe prior to the last universal common ancestor (LUCA), with the SH3 fold emerging via loss of Zn-chelation from a ZnR via acquisition of stronger hydrophobic interactions.

Our findings help clarify the origins and new functions of key domains in chromosomal proteins. Compaction of genomic DNA into the limited intracellular space is a universal challenge for which multiple solutions have been selected across cellular life. In asgardarchaea (believed by many to be the closest sister group of eukaryotes), eukaryotes and euryarchaea this function is carried out by the α-helical histone fold proteins and in bacteria by the HU/IHF superfamily of proteins^79–82^. However, in crenarchaeaota and at least certain bathyarchaeota, the ancestral histones appear to have been displaced by a heterogeneity of chromatin-compacting proteins, namely Cren7, Sul7d and CC1^83^. Based on structural considerations it was earlier suggested that these archaeal chromosomal proteins might have an evolutionary a relationship to the SH3-fold domains of the chromo-like superfamily, which are a hallmark of eukaryotic chromatin proteins^40^. Here, we present a clear evolutionary scenario for the convergent origin of a SH3-like morphology for Cren7/Sul7d-like proteins from ZnRs. We also present evidence for a functional diversification of these ZnRs in crenarchaeaota and bathyarchaeota with potential roles in cell-division comparable to the CdvA-like proteins with the PRC-barrel domain^65,84^.

On the other hand, we present evidence that eukaryotic chromo-like domains have no close relationship to Cren7-like archaeal chromosomal proteins. While there are several SH3 fold β-barrels, eukaryotic chromo-like domains specifically share a peptide-binding function with the classical SH3 domains. We show that both these superfamilies are present in bacterial extracellular proteins and based on the evidence from the bacterial SH3 domains, we posit that the bacterial chromo-like domains too bind peptides in the peptidoglycan or in the periplasm. Further, sequence analysis suggests the bacterial chromo-like domains likely acquired specificity for methylated side-chains of basic amino acids even in these bacterial versions. Further, they might have even interacted with nucleic acids founds in bacterial extracellular matrices. These observations suggest that the two SH3 fold superfamilies initially diverged and radiated primarily in the context of binding different peptides in the bacterial peptidoglycan. Notably, both these show parallel domain architectures (Fig. 3E) and are often coupled with peptidase domains, which might play a role in the degradation of proteins or peptides found in bacterial extracellular matrices. Thus, we predict that both domains might help anchor enzymes regulating the dynamics of the cell-wall or the extracellular matrices as part of cell-cell interactions in course of colony formation or conflicts with other bacteria.

These observations have important implications for the origin of eukaryotes. Bacterial chromo-like domains are found only in certain bacterial lineages, including alphaproteobacteria, unlike the bacterial SH3 domain which is more widely distributed. However, chromo-like domains are rare or absent in archaea (including asgardarchaeota). In contrast, we can trace at least 3 paralogous families of chromo-like domains in the LUCA^38^. Thus, the chromo-like superfamily was present in the stem eukaryote diversified prior to the common ancestor of all extant eukaryotes. This suggests that the eukaryotes possibly acquired their chromo-like and classical SH3 domains from the α-proteobacterial mitochondrial progenitor. Their presence in the extracellular matrix might have facilitated interactions with “cytoplasmic” proteins and nucleic acids of archaeal component of the ancestral eukaryote upon endosymbiosis. Hence, we posit that this was the likely scenario that favoured their recruitment as intracellular peptide-binding domains in the ancestral eukaryote. In eukaryotes, the chromo-like and classical SH3 domains radiated extensively due to the “opening” of entirely new niches in the form of peptide-substrates from histone tails, positively charged RNA-binding complexes and cytoskeletal proteins respectively. This appears to have gone hand-in-hand with the radiation of a diverse suite of enzymes covalently-modifying histones and other proteins^38^. We show that the bacterial chromo-like domains already possessed the capacity to bind peptides through the aromatic cage in the open mouth of the β-barrel; thus, they were pre-adapted to binding methylated peptides in the eukaryotic chromatin niche.

We believe the Cren7-like and chromo-like domains identified in this study might help further experimental characterization of the biochemical and biological diversity of these domains.

## Methods

The PSI-BLAST and JACKHMMER programs were used for iterative sequence profile searches against the National Center for Biotechnology Information (NCBI) non-redundant (NR) and locally generated databases (e.g. nr.50: NR sequences clustered at 50% sequence identity)^61,85^. Additional sequence similarity searches were performed using the HHpred program^63^ (against: PDB70_12Feb17, SCOP95_v1.75B and PfamA_31.0, using MSA generation method HHblits run for 5 iterations, E-value threshold of 0.001). Multiple sequence alignments were constructed using Kalign^86^ followed by manual corrections based on structural alignments. Sequence similarity-based clustering was performed using the BLASTCLUST program (http://ftp.ncbi.nih.gov/blast/documents/blastclust.html), by adjusting the length (L) and score (S) parameters based on need. Automated structure similarity searches were performed using the DALI server^52^. Structures were compared and superimposed in the molecular visualization program PyMOL by manually defining equivalent regions using the pair fitting wizard. Automated pairwise structure superimposition was performed using TM-align and Fr-TM-align tools^59,60^.

Domain architectures and other contextual information about the protein sequences were generated using both Pfam and a custom set of profiles. This analysis was automated using scripts in PERL. Graphs in Fig. 4 were generated using multiple sequence alignments (MSAs) of representative sequences for each family extracted from a database with NR sequences clustered down to 90% identity. The amino acid conservation at the five positions involved in the formation of aromatic cage was extracted from the text version of the multiple sequence alignments generated by us (bacterial chromo domains) or extracted from the Pfam database (previously known clades). Graphs were built using the ggplot2 package in R^87^.

The tree of relationships between different clades of chromo-like domains was generated thus: given that these domains are small in size and show rapid divergence they are not amenable to analysis by conventional phylogenetic methods. Hence, we established the relationships between them using two methods, namely the e-values reported by the profile-profile comparisons with the HHpred program^63^ and the Z-scores using the DALI program. These searches were respectively run using representative sequences or structures for each of the clades against a library of HMM profiles developed from alignments in the Pfam database or structures in PDB. The e-values and Z-scores were recorded for the hits to all other clades. The clades were then clustered using single linkage clustering based on these values and the consensus clustering is rendered as a tree in Fig. 3D.

## Electronic supplementary material

Supplementary Material.
