# Supplementary material for: Evolutionary convergence and divergence in archaeal chromosomal proteins and Chromo-like domains from bacteria and eukaryotes
Source: Sci Rep. 2018 Apr 18;8:6196. doi: 10.1038/s41598-018-24467-z (PMC5906684; doi:10.1038/s41598-018-24467-z)
Supplement: Supplementary file 1 — Supplementary Material. [file 41598_2018_24467_MOESM1_ESM.pdf]

Supplementary Material
Evolutionary convergence and divergence in archaeal chromosomal proteins and Chromo-like domains from bacteria and eukaryotes

Gurmeet Kaur[a], Lakshminarayan M. Iyer[a], Srikrishna Subramanian[b*] and L. Aravind[a*]

* *Address for correspondence: Srikrishna Subramanian (krishna@imtech.res.in); L. Aravind (aravind@mail.nih.gov)*

a. National Center for Biotechnology Information, National Library of Medicine, National Institutes of Health, Bethesda, MD 20894, USA
b. Bioinformatics Department, CSIR-Institute of Microbial Technology, Sector 39, Chandigarh, ZIP 160036, India

Contents

- Supplementary S1: Cren7: Detailed overview of the sequences and domain architectures found in this study.
- Supplementary S2: Cren7: alignments of the major sequence clusters of Cren7 proteins.
- Supplementary S3: Bacterial chromo domains: Detailed overview of the sequences and domain architectures found in this study.
- Supplementary S4: Bacterial chromo domains: multiple sequence alignment of representative sequences
- Supplementary Figure S1. The C-terminal zinc ribbon of nicotinate phosphoribosyltransferase.

- **Supplementary S1: Cren7: Detailed overview of the sequences and domain architectures found in this study.**

# 96; Cren7									
AET34178.1	Cren7	-	Cren7	84	P186_2802	Pyrobaculum ferrireducens	archaea>crenarchaeota	hypothetical protein P186_2802 [Pyrobaculum ferrireducens]	356643499
ABW01588.1	SIG+Cren7	-	Cren7	77	Cmaq_0752	Caldivirga maquilingensis IC-167	archaea>crenarchaeota	hypothetical protein Cmaq_0752 [Caldivirga maquilingensis IC-167]	157920161
ADN50855.1	Cren7	-	Cren7	77	Vdis_1469	Vulcanisaeta distributa DSM 14429	archaea>crenarchaeota	hypothetical protein Vdis_1469 [Vulcanisaeta distributa DSM 14429]	307550790
KJR71736.1	Cren7	-	Cren7	67	TU36_06230	Vulcanisaeta sp. AZ3	archaea>crenarchaeota	chorismate-binding protein [Vulcanisaeta sp. AZ3]	778553605
WP_013337050.1	Cren7	-	Cren7	67	-	Vulcanisaeta distributa	archaea>crenarchaeota	chorismate-binding protein [Vulcanisaeta distributa]	503102265
WP_013603742.1	Cren7	-	Cren7	67	-	Vulcanisaeta moutnovskia	archaea>crenarchaeota	chorismate-binding protein [Vulcanisaeta moutnovskia]	503369081
WP_014027454.1	Cren7	-	Cren7	67	-	Pyrolobus fumarii	archaea>crenarchaeota	chorismate-binding protein [Pyrolobus fumarii]	503793460
WP_054849611.1	Cren7	-	Cren7	67	-	Vulcanisaeta sp. JCM 14467	archaea>crenarchaeota	chorismate-binding protein [Vulcanisaeta sp. JCM 14467]	939164297
WP_054855043.1	Cren7	-	Cren7	67	-	Vulcanisaeta sp. JCM 16161	archaea>crenarchaeota	chorismate-binding protein [Vulcanisaeta sp. JCM 16161]	939169880
WP_069806810.1	Cren7	-	Cren7	67	-	Vulcanisaeta thermophila	archaea>crenarchaeota	chorismate-binding protein [Vulcanisaeta thermophila]	1071374523
WP_012607852.1	Cren7	-	Cren7	65	-	Desulfurococcus	archaea>crenarchaeota	MULTISPECIES: chorismate-binding protein [Desulfurococcus]	501636686
WP_014738044.1	Cren7	-	Cren7	65	-	Thermogladius cellulolyticus	archaea>crenarchaeota	chorismate-binding protein [Thermogladius cellulolyticus]	504550942
WP_048078978.1	Cren7	-	Cren7	65	-	Desulfurococcus mobilis	archaea>crenarchaeota	chorismate-binding protein [Desulfurococcus mobilis]	851129021
Q9YEY2.1	Cren7	-	Cren7	64	-	Aeropyrum pernix K1	archaea>crenarchaeota	RecName: Full=Chromatin protein Cren7	74578551
WP_022541076.1	Cren7	-	Cren7	64	-	Aeropyrum camini	archaea>crenarchaeota	hypothetical protein [Aeropyrum camini]	549634870
WP_075059579.1	Cren7	-	Cren7	64	-	Thermocladium modestius	archaea>crenarchaeota	chorismate-binding protein [Thermocladium modestius]	1125731309
A3MUI7.1	Cren7	-	Cren7	63	-	Pyrobaculum calidifontis JCM 11548	archaea>crenarchaeota	RecName: Full=Chromatin protein Cren7	205828375
KJR71739.1	Cren7	-	Cren7	63	TU36_06215	Vulcanisaeta sp. AZ3	archaea>crenarchaeota	chorismate-binding protein [Vulcanisaeta sp. AZ3]	778553610
KUO79131.1	Cren7	-	Cren7	63	AT718_02035	Vulcanisaeta sp. JCHS_4	archaea>crenarchaeota	chorismate-binding protein [Vulcanisaeta sp. JCHS_4]	974273560
KUO79626.1	Cren7	-	Cren7	63	AT718_08735	Vulcanisaeta sp. JCHS_4	archaea>crenarchaeota	chorismate-binding protein [Vulcanisaeta sp. JCHS_4]	974274092
KUO83820.1	Cren7	-	Cren7	63	AT716_00635	Vulcanisaeta sp. MG_3	archaea>crenarchaeota	chorismate-binding protein [Vulcanisaeta sp. MG_3]	974290626
WP_013335897.1	Cren7	-	Cren7	63	-	Vulcanisaeta	archaea>crenarchaeota	MULTISPECIES: chorismate-binding protein [Vulcanisaeta]	503101107
WP_013336828.1	Cren7	-	Cren7	63	-	Vulcanisaeta distributa	archaea>crenarchaeota	chorismate-binding protein [Vulcanisaeta distributa]	503102042
WP_013603523.1	Cren7	-	Cren7	63	-	Vulcanisaeta moutnovskia	archaea>crenarchaeota	chorismate-binding protein [Vulcanisaeta moutnovskia]	503368862
WP_013604993.1	Cren7	-	Cren7	63	-	Vulcanisaeta moutnovskia	archaea>crenarchaeota	chorismate-binding protein [Vulcanisaeta moutnovskia]	503370332
WP_054849855.1	Cren7	-	Cren7	63	-	Vulcanisaeta sp. JCM 14467	archaea>crenarchaeota	chorismate-binding protein [Vulcanisaeta sp. JCM 14467]	939164541
WP_054849982.1	Cren7	-	Cren7	63	-	Vulcanisaeta sp. JCM 14467	archaea>crenarchaeota	chorismate-binding protein [Vulcanisaeta sp. JCM 14467]	939164668
WP_054850068.1	Cren7	-	Cren7	63	-	Vulcanisaeta sp. JCM 14467	archaea>crenarchaeota	chorismate-binding protein [Vulcanisaeta sp. JCM 14467]	939164754
WP_054854869.1	Cren7	-	Cren7	63	-	Vulcanisaeta sp. JCM 16161	archaea>crenarchaeota	chorismate-binding protein [Vulcanisaeta sp. JCM 16161]	939169706
WP_054854872.1	Cren7	-	Cren7	63	-	Vulcanisaeta sp. JCM 16161	archaea>crenarchaeota	chorismate-binding protein [Vulcanisaeta sp. JCM 16161]	939169709
WP_054855400.1	Cren7	-	Cren7	63	-	Vulcanisaeta	archaea>crenarchaeota	MULTISPECIES: chorismate-binding protein [Vulcanisaeta]	939170237
WP_054857712.1	Cren7	-	Cren7	63	-	Vulcanisaeta sp. JCM 16159	archaea>crenarchaeota	chorismate-binding protein [Vulcanisaeta sp. JCM 16159]	939172732
WP_069807230.1	Cren7	-	Cren7	63	-	Vulcanisaeta thermophila	archaea>crenarchaeota	chorismate-binding protein [Vulcanisaeta thermophila]	1071374952
WP_069808172.1	Cren7	-	Cren7	63	-	Vulcanisaeta thermophila	archaea>crenarchaeota	chorismate-binding protein [Vulcanisaeta thermophila]	1071375928
ADM28106.1	SIG+Cren7	-	Cren7	62	Igag_1302	Ignisphaera aggregans DSM 17230	archaea>crenarchaeota	conserved hypothetical protein [Ignisphaera aggregans DSM 17230]	304378267
ESQ21997.1	Cren7	-	Cren7	62	CISAcid_03660	uncultured Acidilobus sp. CIS	archaea>crenarchaeota	Chromatin protein Cren7 [uncultured Acidilobus sp. CIS]	557076080
ESQ23056.1	Cren7	-	Cren7	62	MGAcid_08750	uncultured Acidilobus sp. MG	archaea>crenarchaeota	Chromatin protein Cren7 [uncultured Acidilobus sp. MG]	557077178
KUO92565.1	Cren7	-	Cren7	62	AT710_02765	Thermocladium sp. ECH_B	archaea>crenarchaeota	chorismate-binding protein [Thermocladium sp. ECH_B]	974327600
KUO93296.1	Cren7	-	Cren7	62	AT710_00200	Thermocladium sp. ECH_B	archaea>crenarchaeota	chorismate-binding protein [Thermocladium sp. ECH_B]	974334366
WP_011822047.1	Cren7	-	Cren7	62	-	Hyperthermus butylicus	archaea>crenarchaeota	chorismate-binding protein [Hyperthermus butylicus]	500146044
WP_011822285.1	Cren7	-	Cren7	62	-	Hyperthermus butylicus	archaea>crenarchaeota	chorismate-binding protein [Hyperthermus butylicus]	500146282
WP_011838541.1	Cren7	-	Cren7	62	-	Staphylothermus marinus	archaea>crenarchaeota	chorismate-binding protein [Staphylothermus marinus]	500163924
WP_013142905.1	Cren7	-	Cren7	62	-	Staphylothermus hellenicus	archaea>crenarchaeota	chorismate-binding protein [Staphylothermus hellenicus]	502907929
WP_055409113.1	Cren7	-	Cren7	62	-	Pyrodictium delaneyi	archaea>crenarchaeota	chorismate-binding protein [Pyrodictium delaneyi]	942705758
WP_058370641.1	Cren7	-	Cren7	62	-	Pyrodictium occultum	archaea>crenarchaeota	chorismate-binding protein [Pyrodictium occultum]	961369292
WP_075058920.1	Cren7	-	Cren7	62	-	Thermocladium modestius	archaea>crenarchaeota	chorismate-binding protein [Thermocladium modestius]	1125730650
AFZ70267.1	Cren7	-	Cren7	61	Calag_0502	Caldisphaera lagunensis DSM 15908	archaea>crenarchaeota	Chromatin protein Cren7 [Caldisphaera lagunensis DSM 15908]	429133255
AMD30944.1	Cren7	-	Cren7	61	SE86_06290	Acidilobus sp. 7A	archaea>crenarchaeota	chorismate-binding protein [Acidilobus sp. 7A]	985631937
EHP68483.1	Cren7	-	Cren7	61	MetMK1DRAFT_00029190	Metallosphaera yellowstonensis MK1	archaea>crenarchaeota	Chromatin protein Cren7 [Metallosphaera yellowstonensis MK1]	373523563
P0C836.2	Cren7	-	Cren7	61	-	Sulfolobus tokodaii str. 7	archaea>crenarchaeota	RecName: Full=Chromatin protein Cren7	363548400
WP_013129173.1	Cren7	-	Cren7	61	-	Thermosphaera aggregans	archaea>crenarchaeota	chorismate-binding protein [Thermosphaera aggregans]	502894197
WP_013266950.1	Cren7	-	Cren7	61	-	Acidilobus saccharovorans	archaea>crenarchaeota	chorismate-binding protein [Acidilobus saccharovorans]	503031974
WP_013562368.1	Cren7	-	Cren7	61	-	Desulfurococcus mucosus	archaea>crenarchaeota	chorismate-binding protein [Desulfurococcus mucosus]	503327707
WP_048062914.1	Cren7	-	Cren7	61	-	Caldivirga maquilingensis	archaea>crenarchaeota	chorismate-binding protein [Caldivirga maquilingensis]	850996080
WP_066797687.1	Cren7	-	Cren7	61	-	Caldivirga sp. MU80	archaea>crenarchaeota	chorismate-binding protein [Caldivirga sp. MU80]	1055047719
AFH42781.1	Cren7	-	Cren7	60	FFONT_0793	Fervidicoccus fontis Kam940	archaea>crenarchaeota	chromatin protein Cren7 [Fervidicoccus fontis Kam940]	383795698
KUO81798.1	Cren7	-	Cren7	60	AT714_06530	Vulcanisaeta sp. OSP_8	archaea>crenarchaeota	chorismate-binding protein [Vulcanisaeta sp. OSP_8]	974282282
KUO82340.1	Cren7	-	Cren7	60	AT718_04470	Vulcanisaeta sp. JCHS_4	archaea>crenarchaeota	chorismate-binding protein [Vulcanisaeta sp. JCHS_4]	974282880
KUO94550.1	Cren7	-	Cren7	60	AT717_06140	Vulcanisaeta sp. CIS_19	archaea>crenarchaeota	chorismate-binding protein, partial [Vulcanisaeta sp. CIS_19]	974341295
WP_009992425.1	Cren7	-	Cren7	60	-	Sulfolobus solfataricus	archaea>crenarchaeota	chorismate-binding protein [Sulfolobus solfataricus]	497678241
WP_012711256.1	Cren7	-	Cren7	60	-	Sulfolobus islandicus	archaea>crenarchaeota	chorismate-binding protein [Sulfolobus islandicus]	502107930
WP_013337544.1	Cren7	-	Cren7	60	-	Vulcanisaeta distributa	archaea>crenarchaeota	chorismate-binding protein [Vulcanisaeta distributa]	503102762
WP_048056861.1	Cren7	-	Cren7	60	-	Vulcanisaeta moutnovskia	archaea>crenarchaeota	chorismate-binding protein [Vulcanisaeta moutnovskia]	850924522
WP_048816690.1	Cren7	-	Cren7	60	-	Caldisphaera lagunensis	archaea>crenarchaeota	chorismate-binding protein [Caldisphaera lagunensis]	889430631
WP_054850299.1	Cren7	-	Cren7	60	-	Vulcanisaeta sp. JCM 14467	archaea>crenarchaeota	chorismate-binding protein [Vulcanisaeta sp. JCM 14467]	939164985
WP_054854845.1	Cren7	-	Cren7	60	-	Vulcanisaeta sp. JCM 16161	archaea>crenarchaeota	chorismate-binding protein [Vulcanisaeta sp. JCM 16161]	939169682
WP_069281685.1	SIG+Cren7	-	Cren7	60	-	Sulfolobus sp. A20	archaea>crenarchaeota	chorismate-binding protein [Sulfolobus sp. A20]	1062514114
WP_069807541.1	Cren7	-	Cren7	60	-	Vulcanisaeta thermophila	archaea>crenarchaeota	chorismate-binding protein [Vulcanisaeta thermophila]	1071375268
A4WIK5.1	Cren7	-	Cren7	59	-	Pyrobaculum arsenaticum DSM 13514	archaea>crenarchaeota	RecName: Full=Chromatin protein Cren7	205828365
AKT34306.1	Cren7	-	Cren7	59	PYWP30_00659	Pyrobaculum sp. WP30	archaea>crenarchaeota	Chromatin protein Cren7 [Pyrobaculum sp. WP30]	908351719
B1YC05.1	Cren7	-	Cren7	59	-	Pyrobaculum neutrophilum V24Sta	archaea>crenarchaeota	RecName: Full=Chromatin protein Cren7	205828497
EWG07100.1	Cren7	-	Cren7	59	ASUL_06253	Sulfolobales archaeon AZ1	archaea>crenarchaeota	hypothetical protein ASUL_06253 [Sulfolobales archaeon AZ1]	583847401
KJR72536.1	Cren7	-	Cren7	59	TU36_01445	Vulcanisaeta sp. AZ3	archaea>crenarchaeota	chorismate-binding protein [Vulcanisaeta sp. AZ3]	778554983
KUO80138.1	Cren7	-	Cren7	59	AT707_03880	Pyrobaculum sp. JCHS_4	archaea>crenarchaeota	chorismate-binding protein [Pyrobaculum sp. JCHS_4]	974274651
Q8ZX17.1	Cren7	-	Cren7	59	-	Pyrobaculum aerophilum str. IM2	archaea>crenarchaeota	RecName: Full=Chromatin protein Cren7	74564364
WP_011278147.1	Cren7	-	Cren7	59	-	Sulfolobus acidocaldarius	archaea>crenarchaeota	chorismate-binding protein [Sulfolobus acidocaldarius]	499597413
WP_011763327.1	Cren7	-	Cren7	59	-	Pyrobaculum islandicum	archaea>crenarchaeota	chorismate-binding protein [Pyrobaculum islandicum]	500087314
WP_012021704.1	Cren7	-	Cren7	59	-	Metallosphaera sedula	archaea>crenarchaeota	chorismate-binding protein [Metallosphaera sedula]	500928566
WP_013737071.1	Cren7	-	Cren7	59	-	Metallosphaera cuprina	archaea>crenarchaeota	chorismate-binding protein [Metallosphaera cuprina]	503502410
WP_013776226.1	Cren7	-	Cren7	59	-	Acidianus hospitalis	archaea>crenarchaeota	chorismate-binding protein [Acidianus hospitalis]	503542150
WP_014126920.1	Cren7	-	Cren7	59	-	Thermoproteus tenax	archaea>crenarchaeota	chorismate-binding protein [Thermoproteus tenax]	503892926
WP_048088114.1	Cren7	-	Cren7	59	-	Metallosphaera yellowstonensis	archaea>crenarchaeota	chorismate-binding protein [Metallosphaera yellowstonensis]	851153362
WP_048099311.1	SIG+Cren7	-	Cren7	59	-	Candidatus Acidianus copahuensis	archaea>crenarchaeota	chorismate-binding protein [Candidatus Acidianus copahuensis]	851185348
WP_054837783.1	Cren7	-	Cren7	59	-	Sulfolobus metallicus	archaea>crenarchaeota	chorismate-binding protein [Sulfolobus metallicus]	939151330
WP_054845567.1	Cren7	-	Cren7	59	-	Sulfolobus sp. JCM 16833	archaea>crenarchaeota	chorismate-binding protein [Sulfolobus sp. JCM 16833]	939159665
KJR73860.1	Cren7	-	Cren7	58	TU35_03430	Thermoproteus sp. AZ2	archaea>crenarchaeota	chorismate-binding protein [Thermoproteus sp. AZ2]	778567493
KUO85030.1	Cren7	-	Cren7	58	AT711_01510	Thermoproteus sp. CIS_19	archaea>crenarchaeota	chorismate-binding protein [Thermoproteus sp. CIS_19]	974297700
WP_013679529.1	Cren7	-	Cren7	58	-	Thermoproteus uzoniensis	archaea>crenarchaeota	chorismate-binding protein [Thermoproteus uzoniensis]	503444868
WP_052846948.1	Cren7	-	Cren7	58	-	Sulfolobus tokodaii	archaea>crenarchaeota	chorismate-binding protein [Sulfolobus tokodaii]	919521076
3KXT_A	Cren7	-	Cren7	56	-	Sulfolobus solfataricus	archaea>crenarchaeota	Chain A, Crystal Structure Of Sulfolobus Cren7-Dsdna Complex	297787667
WP_015231698.1	Cren7	-	Cren7	56	-	Caldisphaera lagunensis	archaea>crenarchaeota	hypothetical protein [Caldisphaera lagunensis]	505044596
4R55_A	Cren7	-	Cren7	55	-	Sulfolobus solfataricus P2	archaea>crenarchaeota	Chain A, The Crystal Structure Of A Cren7 Mutant Protein Gr And Dsdna Complex	910750645
WP_011998075.1	Cren7	-	Cren7	55	-	Ignicoccus hospitalis	archaea>crenarchaeota	chorismate-binding protein [Ignicoccus hospitalis]	500798642
WP_075049584.1	Cren7	-	Cren7	54	-	Ignicoccus islandicus	archaea>crenarchaeota	chorismate-binding protein [Ignicoccus islandicus]	1125708117
ADY01022.1	Cren7	-	Cren7	53	VMUT_0812	Vulcanisaeta moutnovskia 768-28	archaea>crenarchaeota	hypothetical protein VMUT_0812 [Vulcanisaeta moutnovskia 768-28]	323707535
KYH37633.1	CpXC	-	CpXC	51	AYL29_004170	Candidatus Bathyarchaeota archaeon B24	archaea	hypothetical protein AYL29_004170 [Candidatus Bathyarchaeota archaeon B24]	1007509441
# 1; Bathyarchaeal Cren7 with N-terminal tubulin; Cren7 is sequentially similar to classical Cren7									
KYH38952.1	Tubulin	-	Tubulin	420	AYL30_002000	Candidatus Bathyarchaeota archaeon B25	archaea	putative cell division protein [Candidatus Bathyarchaeota archaeon B25]	1007510856
# 9; Cren7 with N-terminal CdvA coiled-coil and middle 3-stranded beta-rich region									
OLD03897.1	Atrophin-1	-	Atrophin-1	382	AUJ07_05410	Crenarchaeota archaeon 13_1_40CM_3_53_5	archaea>crenarchaeota	hypothetical protein AUJ07_05410 [Crenarchaeota archaeon 13_1_40CM_3_53_5]	1125365454
OLE70579.1	CCDC158	-	CCDC158	378	AUF78_05925	archaeon 13_1_20CM_2_51_12	archaea	hypothetical protein AUF78_05925 [archaeon 13_1_20CM_2_51_12]	1125560451
OLD55234.1	-	-	-	377	AUI46_05505	archaeon 13_1_40CM_2_52_13	archaea	hypothetical protein AUI46_05505 [archaeon 13_1_40CM_2_52_13]	1125424927
OLE70912.1	-	-	-	377	AUF78_04475	archaeon 13_1_20CM_2_51_12	archaea	hypothetical protein AUF78_04475 [archaeon 13_1_20CM_2_51_12]	1125560928
OLE68285.1	CCDC158+GGN	-	CCDC158+GGN	365	AUF78_17050	archaeon 13_1_20CM_2_51_12	archaea	hypothetical protein AUF78_17050 [archaeon 13_1_20CM_2_51_12]	1125557313
OLB70622.1	-	-	-	363	AUI06_06110	archaeon 13_2_20CM_2_52_21	archaea	hypothetical protein AUI06_06110 [archaeon 13_2_20CM_2_52_21]	1125221443
OLE92038.1	Herpes_BLLF1	-	Herpes_BLLF1	363	AUF79_00800	Crenarchaeota archaeon 13_1_20CM_2_51_8	archaea>crenarchaeota	hypothetical protein AUF79_00800 [Crenarchaeota archaeon 13_1_20CM_2_51_8]	1125586675
OLE91878.1	-	-	-	361	AUF79_01805	Crenarchaeota archaeon 13_1_20CM_2_51_8	archaea>crenarchaeota	hypothetical protein AUF79_01805 [Crenarchaeota archaeon 13_1_20CM_2_51_8]	1125586418
OLD10777.1	-	-	-	332	AUI93_06395	Crenarchaeota archaeon 13_1_40CM_3_52_10	archaea>crenarchaeota	hypothetical protein AUI93_06395 [Crenarchaeota archaeon 13_1_40CM_3_52_10]	1125372930
# 2; Bathyarchaeal Cren7 with a longer region in between 2 zinc knuckles ; but sequence features are closer to classical Cren7									
KPV61655.1	-	-	-	56	AOA66_1625	Candidatus Bathyarchaeota archaeon BA2	archaea	hypothetical protein AOA66_1625 [Candidatus Bathyarchaeota archaeon BA2]	939571725
KPV62727.1	-	-	-	55	AOA65_1665	Candidatus Bathyarchaeota archaeon BA1	archaea	hypothetical protein AOA65_1665 [Candidatus Bathyarchaeota archaeon BA1]	939572832
# 13; Cren7 with 4 cysteines									
KUO92830.1	-	-	-	77	AT710_02125	Thermocladium sp. ECH_B	archaea>crenarchaeota	hypothetical protein AT710_02125 [Thermocladium sp. ECH_B]	974333874
AET34264.1	-	-	-	75	P186_2888	Pyrobaculum ferrireducens	archaea>crenarchaeota	hypothetical protein P186_2888 [Pyrobaculum ferrireducens]	356643585
WP_012608538.1	-	-	-	68	-	Desulfurococcus amylolyticus	archaea>crenarchaeota	hypothetical protein [Desulfurococcus amylolyticus]	501637966
WP_014767718.1	-	-	-	68	-	Desulfurococcus amylolyticus	archaea>crenarchaeota	hypothetical protein [Desulfurococcus amylolyticus]	504580616
WP_012186112.1	-	-	-	66	-	Caldivirga maquilingensis	archaea>crenarchaeota	hypothetical protein [Caldivirga maquilingensis]	501137594
WP_066795265.1	tRNA-synt_1f	-	tRNA-synt_1f	63	-	Caldivirga sp. MU80	archaea>crenarchaeota	hypothetical protein [Caldivirga sp. MU80]	1055045297
ABO08140.1	-	-	-	60	Pcal_0714	Pyrobaculum calidifontis JCM 11548	archaea>crenarchaeota	conserved hypothetical protein [Pyrobaculum calidifontis JCM 11548]	126249049
AFA38036.1	-	-	-	60	Pogu_0009	Pyrobaculum oguniense TE7	archaea>crenarchaeota	hypothetical protein Pogu_0009 [Pyrobaculum oguniense TE7]	375158424
WP_013680511.1	-	-	-	60	-	Thermoproteus uzoniensis	archaea>crenarchaeota	hypothetical protein [Thermoproteus uzoniensis]	503445850
AAL62651.1	-	-	-	58	PAE0240	Pyrobaculum aerophilum str. IM2	archaea>crenarchaeota	conserved within P. aerophilum [Pyrobaculum aerophilum str. IM2]	18159210
AFA38724.1	-	-	-	58	Pogu_0697	Pyrobaculum oguniense TE7	archaea>crenarchaeota	hypothetical protein Pogu_0697 [Pyrobaculum oguniense TE7]	375159112
ABP51071.1	-	-	-	56	Pars_1515	Pyrobaculum arsenaticum DSM 13514	archaea>crenarchaeota	conserved hypothetical protein [Pyrobaculum arsenaticum DSM 13514]	145283489
AAL63503.1	-	-	-	49	PAE1469	Pyrobaculum aerophilum str. IM2	archaea>crenarchaeota	conserved within P. aerophilum [Pyrobaculum aerophilum str. IM2]	18160128
# 1; Cren7 with unknown domain at C-terminal									
AFA38530.1	-	-	-	191	Pogu_0503	Pyrobaculum oguniense TE7	archaea>crenarchaeota	hypothetical protein Pogu_0503 [Pyrobaculum oguniense TE7]	375158918
# 1; Nanoarchaeal Cren7 with C-terminal REase fold domain									
AMD29662.1	Zn_Tnp_IS1595	-	Zn_Tnp_IS1595	201	Nps_00900	Candidatus Nanopusillus acidilobi	archaea>nanoarchaeota	hypothetical protein Nps_00900 [Candidatus Nanopusillus acidilobi]	985630599
# 42; Cren7ZnR									
KPV64287.1	Spc7	-	Spc7	135	AOA66_0247	Candidatus Bathyarchaeota archaeon BA2	archaea	hypothetical protein AOA66_0247 [Candidatus Bathyarchaeota archaeon BA2]	939574455
WP_012123536.1	SIG	Nanos	-	118	-	Ignicoccus hospitalis	archaea>crenarchaeota	hypothetical protein [Ignicoccus hospitalis]	501072520
WP_048099394.1	CpXC	-	CpXC	114	-	Candidatus Acidianus copahuensis	archaea>crenarchaeota	hypothetical protein [Candidatus Acidianus copahuensis]	851185623
WP_054837835.1	SIG	-	-	110	-	Sulfolobus metallicus	archaea>crenarchaeota	hypothetical protein [Sulfolobus metallicus]	939151382
WP_054845496.1	SIG+CpXC	-	CpXC	110	-	Sulfolobus sp. JCM 16833	archaea>crenarchaeota	hypothetical protein [Sulfolobus sp. JCM 16833]	939159594
AMD30850.1	-	-	-	109	SE86_05640	Acidilobus sp. 7A	archaea>crenarchaeota	chromatin protein Cren7 [Acidilobus sp. 7A]	985631843
WP_009992346.1	CpXC	-	CpXC	109	-	Sulfolobus solfataricus	archaea>crenarchaeota	hypothetical protein [Sulfolobus solfataricus]	497678162
WP_012711312.1	SIG+zf-ZPR1	-	zf-ZPR1	109	-	Sulfolobus islandicus	archaea>crenarchaeota	hypothetical protein [Sulfolobus islandicus]	502108019
WP_014512614.1	zf-ZPR1	-	zf-ZPR1	109	-	Sulfolobus islandicus	archaea>crenarchaeota	hypothetical protein [Sulfolobus islandicus]	504325512
WP_069281853.1	-	-	-	109	-	Sulfolobus sp. A20	archaea>crenarchaeota	chromatin protein Cren7 [Sulfolobus sp. A20]	1062514282
WP_075049443.1	SIG+CpXC	-	CpXC	109	-	Ignicoccus islandicus	archaea>crenarchaeota	chromatin protein Cren7 [Ignicoccus islandicus]	1125707976
AFH42126.1	SIG	-	-	108	FFONT_0134	Fervidicoccus fontis Kam940	archaea>crenarchaeota	hypothetical protein FFONT_0134 [Fervidicoccus fontis Kam940]	383795043
WP_012021609.1	CpXC	-	CpXC	107	-	Metallosphaera sedula	archaea>crenarchaeota	hypothetical protein [Metallosphaera sedula]	500928367
WP_013776128.1	CpXC	-	CpXC	107	-	Acidianus hospitalis	archaea>crenarchaeota	hypothetical protein [Acidianus hospitalis]	503542052
ADM27988.1	-	-	-	106	Igag_1182	Ignisphaera aggregans DSM 17230	archaea>crenarchaeota	conserved hypothetical protein [Ignisphaera aggregans DSM 17230]	304378149
EWG07001.1	SIG+tRNA-synt_1f	-	tRNA-synt_1f	106	ASUL_05758	Sulfolobales archaeon AZ1	archaea>crenarchaeota	hypothetical protein ASUL_05758 [Sulfolobales archaeon AZ1]	583847302
WP_009074728.1	-	-	-	106	-	Metallosphaera yellowstonensis	archaea>crenarchaeota	hypothetical protein [Metallosphaera yellowstonensis]	496365738
WP_010979229.1	SIG	-	-	106	-	Sulfolobus tokodaii	archaea>crenarchaeota	hypothetical protein [Sulfolobus tokodaii]	499287939
WP_011839555.1	SIG	-	-	106	-	Staphylothermus marinus	archaea>crenarchaeota	hypothetical protein [Staphylothermus marinus]	500164939
WP_013129045.1	SIG	ZNR	-	106	-	Thermosphaera aggregans	archaea>crenarchaeota	hypothetical protein [Thermosphaera aggregans]	502894069
WP_013143477.1	SIG	-	-	106	-	Staphylothermus hellenicus	archaea>crenarchaeota	hypothetical protein [Staphylothermus hellenicus]	502908501
WP_013266749.1	-	-	-	106	-	Acidilobus saccharovorans	archaea>crenarchaeota	hypothetical protein [Acidilobus saccharovorans]	503031773

WP_013737166.1	CpXC	-	CpXC	106	-	Metallosphaera cuprina	archaea>crenarchaeota	hypothetical protein [Metallosphaera cuprina]	503502505
AHC51640.1	tRNA-synt_1f	-	tRNA-synt_1f	105	SUSAZ_06595	Sulfolobus acidocaldarius SUSAZ	archaea>crenarchaeota	Chromatin protein Cren7 [Sulfolobus acidocaldarius SUSAZ]	566047755
WP_011278214.1	tRNA-synt_1f	-	tRNA-synt_1f	105	-	Sulfolobus acidocaldarius	archaea>crenarchaeota	hypothetical protein [Sulfolobus acidocaldarius]	499597480
WP_055409861.1	SIG+CpXC	-	CpXC	105	-	Pyrodictium delaneyi	archaea>crenarchaeota	hypothetical protein [Pyrodictium delaneyi]	942706506
ESQ20288.1	-	-	-	103	CISAcid_17640	uncultured Acidilobus sp. CIS	archaea>crenarchaeota	hypothetical protein CISAcid_17640 [uncultured Acidilobus sp. CIS]	557074306
ESQ25549.1	SIG	-	-	103	JCHSAcid_04860	uncultured Acidilobus sp. JCHS	archaea>crenarchaeota	hypothetical protein JCHSAcid_04860 [uncultured Acidilobus sp. JCHS]	557079740
ESQ22624.1	-	-	-	102	MGAcid_12280	uncultured Acidilobus sp. MG	archaea>crenarchaeota	hypothetical protein MGAcid_12280 [uncultured Acidilobus sp. MG]	557076731
ESQ26964.1	-	-	-	102	OSP8Acid_00570	uncultured Acidilobus sp. OSP8	archaea>crenarchaeota	hypothetical protein OSP8Acid_00570 [uncultured Acidilobus sp. OSP8]	557081184
WP_058371372.1	SIG+CpXC	-	CpXC	102	-	Pyrodictium occultum	archaea>crenarchaeota	hypothetical protein [Pyrodictium occultum]	961370023
WP_012609105.1	-	-	-	101	-	Desulfurococcus kamchatkensis	archaea>crenarchaeota	hypothetical protein [Desulfurococcus kamchatkensis]	501639024
WP_014766879.1	zinc_ribbon_13	-	zinc_ribbon_13	101	-	Desulfurococcus fermentans	archaea>crenarchaeota	hypothetical protein [Desulfurococcus fermentans]	504579777
WP_015231931.1	OrfB_Zn_ribbon	ZNR	OrfB_Zn_ribbon	101	-	Caldisphaera lagunensis	archaea>crenarchaeota	hypothetical protein [Caldisphaera lagunensis]	505044829
WP_042666841.1	zinc_ribbon_13	-	zinc_ribbon_13	101	-	Desulfurococcus amylolyticus	archaea>crenarchaeota	chromatin protein Cren7 [Desulfurococcus amylolyticus]	756978781
WP_011822080.1	SIG+CpXC	Nanos	CpXC	100	-	Hyperthermus butylicus	archaea>crenarchaeota	hypothetical protein [Hyperthermus butylicus]	500146077
WP_013562562.1	SIG	-	-	100	-	Desulfurococcus	archaea>crenarchaeota	MULTISPECIES: hypothetical protein [Desulfurococcus]	503327901
WP_022542275.1	-	-	-	99	-	Aeropyrum camini	archaea>crenarchaeota	hypothetical protein [Aeropyrum camini]	549637142
BAA81469.2	-	-	-	98	APE_2454.1	Aeropyrum pernix K1	archaea>crenarchaeota	hypothetical protein APE_2454.1 [Aeropyrum pernix K1]	116063198
WP_014027473.1	SIG+CpXC	Nanos	CpXC	97	-	Pyrolobus fumarii	archaea>crenarchaeota	hypothetical protein [Pyrolobus fumarii]	503793479
WP_014736716.1	-	ZNR	-	97	-	Thermogladius cellulolyticus	archaea>crenarchaeota	hypothetical protein [Thermogladius cellulolyticus]	504549614
KPV62726.1	-	-	-	71	AOA65_1664	Candidatus Bathyarchaeota archaeon BA1	archaea	hypothetical protein AOA65_1664 [Candidatus Bathyarchaeota archaeon BA1]	939572831
# 1 Bathyarcheal Cren7 with N-terminal URI domain GIY-YIG endonuclease and middle OrfY-like domain ; Cren7 is partial and sequentially closer to Cren7ZnR									
KPV62179.1	-	-	-	225	uvrC	Candidatus Bathyarchaeota archaeon BA2	archaea	UvrABC system protein C [Candidatus Bathyarchaeota archaeon BA2]	939572257

Back to Contents

- **Supplementary S2: Cren7: alignments of the major sequence clusters of Cren7 proteins.**

```
# Cren7
FINAL            -------------EEEEE---EEEEEEEEEEEEE------E--EEEEEEE-----E-E-EEEEE-----
AET34178.1       ---AEEILN-R-EYEVEWGGRVYRLRPVKAWILQPPG-KPG--VVVALFRLPD-GR-T-VRRVIMRLPP
ABW01588.1       AVNVQQYLN-K-EYEVECDGQMVRLKPVKAWVLQPKG-RKG--VVIGLFKCPN-GK-T-LRKAIGKIE-
ADN50855.1       MVTLEDLAK-R-EYEVEGRK----LRPTKVWKVQPKG-RKG--FVMALFKTPD-GK-T-VRKVIAKVDE
KJR71736.1       MVSLEDLAK-K-EYEIEYEGSKYKLKPTKVWKVQPKG-RKG--FVMGLFRLPT-GK-V-ARKVLAKLDE
WP_013337050.1   MVKLEDLAK-K-EYEIEYEGSKYKLKPTKVWKVQPKG-RKG--FVMALFKLPT-GK-V-VRKVVAKVDE
WP_013603742.1   MVKLEDLAK-K-EYEVEYEGSKYKLKPTKVWKVQPKG-RKG--FVMALFRLPT-GK-V-VRKVVAKVDE
WP_014027454.1   TKPVRVRDSST-GEEVE-------LVPIKVWVLAPRG-RKG--VKIGLFKSPKTGK-Y-FRAKVPDDYP
WP_054849611.1   MVKLEDLAK-K-DYEIEYEGSKYKLKPTKVWKVQPKG-RKG--FVMALFRLPT-GK-V-VRKVVAKVDE
WP_054855043.1   MVKLEDLAK-K-EYEIEYEGSKYKLKPTKVWKVQPKG-RKG--FVMALFRLPT-GK-V-VRKVVAKVDE
WP_069806810.1   MVTLEDLAK-R-EYEIEVEGKKYRLKPTKVWKVQPQG-RKG--FVTGLFKTPD-GK-T-VRKVIAKLDE
WP_012607852.1   TEKVKVKTP-G-GRELE-------LVPVKVWQLNPRG-RKG--VKIGLFQDPGTGR-Y-FRTRVPEEYP
WP_014738044.1   KNAVKVRTP-S-GKELE-------LIPRKVWQLTPSG-RKG--VKVGLFQDPETGK-F-FRQKVPDDYP
WP_048078978.1   TEKVKVKTP-S-GRELE-------LVPVKVWQLNPRG-RKG--VKVGLFQDPESGR-Y-FRTKVPENYP
Q9YEY2.1         LPPVKVRDPTT-GKEVE-------LTPIKVWKLSPRG-RRG--VKIGLFKSPETGK-Y-FRAKVPDDYP
WP_022541076.1   LPPVKVRDPTT-GKEVE-------LTPIKVWKLSPRG-RRG--VKIGLFKSQETGK-Y-FRAKVPDDYP
WP_075059579.1   -VNTQEYMGGK-EYEVEYEGHKYKLKPLKVWVLQPRGGRKG--IVIGLFQLPN-GK-K-IRKAIGKIE-
A3MUI7.1         ---AEEILN-K-EYEVVYEGKRFLLKPAKAWVLQPPG-KPG--VIVALFKLPN-GK-T-VRKVIARLPP
KJR71739.1       MVSLEDLAK-R-EYEVEGRK----LKPTKVWKVQPKG-RKG--FVMGLFRLPT-GK-V-ARKVLAKLDE
KUO79131.1       MVSLEDLAK-K-EYEVEGYK----LKPTKVWKVQPKG-RKG--FVMALFKTPD-GK-T-VRKVIAKVDE
KUO79626.1       MVKLEDLAK-K-EYEVEGHK----LKPTKVWKVQPKG-RKG--FVMALFKTPD-GK-T-VRKVIAKVDE
KUO83820.1       MVSLEDLAK-R-EYEVEGRK----LKPTKVWKVQPKG-RKG--FVMALFKTPD-GK-T-VRKVIAKVDE
WP_013335897.1   MVTLEDLAK-R-EYEIEGKK----LKPTKVWKVQPKG-RKG--FVMALFKTPD-GK-T-VRKVIAKVDE
WP_013336828.1   MVTLEDLAK-R-EYEVEGRK----LRPTKVWKVQPKG-RKG--FVMALFKTPD-GK-T-VRKVIAKVDE
WP_013603523.1   MVSLEDLAR-K-EYEVEGRR----LKPSKVWKVQPKG-RKG--FVMALFKTPD-GK-T-VRKVIAKVDE
WP_013604993.1   MVTLEDLAR-K-EYEVEGRK----LKPTKVWKVQPKG-RKG--FVMALFKTPD-GK-T-VRKVIAKVDE
WP_054849855.1   MVSLEDLAK-R-EYEVEGRK----LRPTKVWKVQPKG-RKG--FVMALFKTPD-GK-T-VRKVIAKVDE
WP_054849982.1   MVTLEDLAK-K-EYEVEGHR----LKPTKVWKVQPKG-RKG--FVMALFKTPD-GK-T-VRKVIAKVDE
WP_054850068.1   MVSLEDLAK-R-EYEVEGRK----LRPTKVWKVQPKG-RKG--FVMALFKTPD-GK-T-VRKVIAKVDE
WP_054854869.1   MVSLEDLAK-R-EYDVEGRR----LKPTKVWKVQPKG-RKG--FVMALFKTPD-GK-T-VRKVIAKVDE
WP_054854872.1   MVTLEDLAK-R-EYDVEGRR----LKPTKVWKVQPKG-RKG--FVMALFKTPD-GK-T-VRKVIAKVDE
WP_054855400.1   MVTLEDLAK-K-EYEVEGKK----LKPTKVWKVQPKG-RKG--FVMALFKTPD-GK-T-VRKVIAKVDE
WP_054857712.1   MVTLEDLAK-K-EYEVEGRR----LRPTKVWKVQPKG-RKG--FVMALFKTPD-GK-T-VRKVIAKVDE
WP_069807230.1   MVTLEDLAK-R-EYEVEGRK----LKPTKVWKVQPKG-RKG--FVMALFKTPE-GK-T-VRKVIAKVDE
WP_069808172.1   MVTLEDLAR-R-EYEVEGRK----LKPTKVWKVQPKG-RKG--FVMALFKTPE-GK-T-VRKVIAKVDE
ADM28106.1       TKPVRVKTP-D-GAEKE-------LVPKKVWGLAPKG-RKG--VKIGLFQDPATGK-Y-FRGKVPDDYP
ESQ21997.1       LPPVKVKDPTT-GKEVE-------LAPIKVWTLAPKT-RKG--VKIGLFKSPETGK-F-FRAKVPDDYP
ESQ23056.1       LPPVKVKDPTT-GKEVE-------LTPIKVWTLAPKT-RKG--VKIGLFKSPETGK-F-FRAKVPDDYP
KUO92565.1       PVNLQDYQK-K-EYDVECDGRITKLKPIKVWTLAPKG-RKG--VVIGLFKCPN-GK-T-IRKSIGKIEP
KUO93296.1       -VNTQEYMG-K-EYEVEYEGHKYKLKPLKVWVLQPRG-RKG--IVIGLFQLPN-GK-K-IRKAIGKIE-
WP_011822047.1   EKPVKVRDPTT-GKEVE-------LVPIKVWQLAPKG-RKG--VKIGLFKSPETGK-Y-FRAKVPDDYP
WP_011822285.1   EKPVKVRDPTT-GKEVE-------LVPIKVWQLAPRG-RKG--VKIGLFKSPETGK-Y-FRAKVPDDYP
WP_011838541.1   KDAVKVKTL-S-GKEVE-------LVPKKVWQLSPKG-RKG--VKVGLFQDPETGK-Y-FRAKVPDDYP
WP_013142905.1   TKSVKVRTP-S-GKEVE-------LVPKKVWQLSPKG-RKG--VKVGLFQDPETGK-Y-FRAKVPDDYP
WP_055409113.1   EKAVKVRDPTT-GKEVE-------LVPIKVWQLAPRG-RKG--VKIGLFKSPETGK-Y-FRAKVPDDYP
WP_058370641.1   EKAVRVRDPTT-GKEAE-------LVPIKVWQLAPRG-RKG--VKIGLFKSPETGK-Y-FRAKVPDDYP
WP_075058920.1   PVNLQDYQK-K-EYDVECDGRTTKLKPIKVWTLAPKG-RKG--VVIGLFKCPN-GK-T-IRKSIGKIEP
AFZ70267.1       FPPVKVKDPSS-GKEVE-------LSPIKVWTLAPKS-RKG--VKIGLFKSPETGK-F-FRAKVPENYP
AMD30944.1       LPAVRVKDPTT-GKDVE-------LAPIKVWTLAPKT-RKG--VKIGLFKSPETGK-F-FRAKVPDDYP
EHP68483.1       KKAVKVKTP-G-GKEMD-------LTPEKAWTLAPKG-RKG--VKIGLFKDPETGK-Y-FRHKLPDDYA
P0C836.2         EKKVKVKTP-S-GKEAE-------LAPEKVWVLAPKG-RKG--VKIGLFKDPETGK-Y-FRHKLPDDYP
WP_013129173.1   -KPVKVKTA-T-GKELE-------LIPKKVWQLNPRG-KKG--VKVGLFQDPETGT-F-FRAKVPEDYP
WP_013266950.1   LPAVKVKDPTT-GKEVE-------LSPIKVWTLAPKT-RKG--VKIGLFKSPESGK-F-FRAKVPDDYP
WP_013562368.1   TEKVKVKTP-S-GRELE-------LVPVKVWQLNPRG-RKG--VKVGLFQDPESGR-Y-FRTKVPENYP
WP_048062914.1   AVNVQQYLN-K-EYEVECDGQMVRLKPVKAWVLQPKG-RKG--VVIGLFKCPN-GK-T-LRKAIGKIE-
WP_066797687.1   AVNVQQYLG-K-EYEVECDGQMVKLKPVKAWVLQPKG-RKG--VVIGLFKCPN-GK-T-IRKAIGKIE-
AFH42781.1       KRPVKVKDRIT-GKEYE-------LVPIKVWKLSPNK-KRG--IKMGLFQNPE-TKIY-FRARVPDDYP
KUO81798.1       MSEFSSYME-K-EYEVECDGQIVKLKPVKVWMLAPKG-RRG--VIIGLFKCPS-GK-V-VRKAIGKAE-
KUO82340.1       MSEFSSYME-R-EYEVECDGQIVKLKPVKVWMLAPKG-RRG--VIIGLFKCPS-GK-V-VRKAIGKAE-
KUO94550.1       MVKLEDLAK-K-EYEVEGHK----LKPTKVWKVQPKG-RKG--FVMALFKTPD-GK-T-VRKVIAKVDE
WP_009992425.1   KKPVKVKTP-A-GKEAE-------LVPEKVWALAPKG-RKG--VKIGLFKDPETGK-Y-FRHKLPDDYP
WP_012711256.1   KKAVKVKTP-A-GKEAE-------LVPEKVWALAPKG-RKG--VKIGLFKDPETGK-Y-FRHKLPDDYP
WP_013337544.1   MAELSSYME-K-EYEVECDGQIVKLKPVKVWMLAPKG-RRG--VIIGLFKCPG-GK-V-VRKAIGKAE-
WP_048056861.1   MAELSSYME-K-EYEVECDGQSMKLKPVKVWMLAPKG-RRG--VIIGLFKCPG-GK-V-VRKAIGKAE-
WP_048816690.1   FPPVKVKDPSS-GKEVE-------LSPIKVWTLAPKS-RKG--VKIGLFKSPETGK-F-FRAKVPENYP
WP_054850299.1   MAELSSYME-K-EYEIDCDGQIVKLKPVKVWMLAPKG-RRG--VIIGLFKCPN-NK-V-VRKALGRAE-
WP_054854845.1   MAELSSYME-K-EYEVECDGQVVKLKPVKVWMLAPKG-RRG--VIIGLFKCPG-GK-V-VRKAIGKAE-
WP_069281685.1   KKSVKVKTP-N-GKEIE-------LVPEKAWALAPKG-RKG--VKIGLFKDPETGK-Y-FRHKLPDDYP
WP_069807541.1   MAELSSYLE-K-EYDVECDGQVVKLKPVKVWMLAPKG-RRG--VIIGLFRCPS-GK-T-VRKAIGRAE-
A4WIK5.1         ---AEEILN-R-EYEVEYGGKRYILRPIKAWVLQPPG-KPG--VVVALFRLPD-GK-T-VRKVVMKLPP
AKT34306.1       ---AEEILN-R-EYEVEYAGRVYWLKPVKAWVLQPPG-KPG--VVVALFKLPD-GK-T-VRKVVMRLPP
B1YCQ5.1         ---AEEILN-R-EYEVEYGGKRYWLRPSKAWVLQPPG-KPG--VVIALFKLPD-GR-T-VRKAIMRLPP
EWG07100.1       KKAVKVKTP-A-GKEME-------LSPEKVWTLAPKG-RKG--VKIGLFKDPETGK-Y-FRHKLPDDYP
KJR72536.1       MEELSSHME-R-EYEVDCDGQIMKLKPIRVWVLAPKG-RRG--VIIGLFKCPS-GK-A-VRKAIGK--E
KUO80138.1       ---AEEILN-S-IYEVEYAGRVYWLKPVKAWVLQPPG-KPG--VVVALFKLPD-GK-T-VRKVVMRLPP
Q8ZX17.1         ---AEEILN-R-EYEVEYEGRKYFLRPVKAWVLQPPG-KPG--VVVALFKLPN-GK-S-IRKVIMRLPP
WP_011278147.1   KKRVRVRTP-G-GKELE-------LTPEKTWVLAPKG-RKG--VKIGLFKDPESGK-Y-FRHKLPDDYP
WP_011763327.1   ---MEEVLD-R-EYEVEYGGRKYRLKPVKAWVLQPPG-KPG--VVIALFKLPD-GK-T-IRKVIMKLPP
WP_012021704.1   KKAVKIKTP-G-GKEAE-------LAPEKAWTLAPKG-RKG--VKIGLFKDPESGK-Y-FRHKLPDDYP
WP_013737071.1   KKQVKIKTP-G-GKEAE-------LAPEKAWTLAPKG-RKG--VKIGLFKDPESGK-Y-FRHKLPDDYP
WP_013776226.1   KKAIKVKTP-S-GKEVE-------LAPEKAWSLAPKG-RKG--VKIGLFKDPETGK-Y-FRRKLPDDYQ
WP_014126920.1   ---MSDILD-K-EYEVVVEGRRYMLKPEKAWVLQPPG-KPG--VVVALFRTPD-GK-V-VRRVIARVPP
WP_048088114.1   KKAVKVKTP-G-GKEMD-------LTPEKAWTLAPKG-RKG--VKIGLFKDPETGK-Y-FRHKLPDDYA
WP_048099311.1   KKTVKVKTP-S-GKEAE-------LSPEKAWSLAPKG-RKG--VKIGLFKDPDTGK-Y-FRHKLPDDYQ
WP_054837783.1   SKKVKVRTP-A-GKEAE-------LIPEKTWTLAPRG-RKG--VKMGLFKDPESGR-Y-FRRKIPDDYP
WP_054845567.1   SKKVKVRTP-G-GKEAE-------LIPEKTWTLAPKG-RKG--VKIGLFKDPESGK-Y-FRHKLPDDYP
KJR73860.1       ---MAELLD-Q-EYEVEVGGRRYKLKPEKVWVLQPKG-KPG--IVIALFRTPD-GK-T-LRKVIAKLPP
KUO85030.1       ---MSELLD-R-EFEVEVGGKKYLLKPEKVWVLQPPG-KPG--VVIASFKTPD-GR-K-VRKVVARLPP
WP_013679529.1   ---MSDLLD-R-EFVVEVGGEKYRLKPEKIWVLQPPG-KPG--VVIALFKTPD-GK-K-VRKVVARLPP
WP_052846948.1   EKKVKVKTP-S-GKEAE-------LAPEKVWVLAPKG-RKG--VKIGLFKDPETGK-Y-FRHKLPDDYP
3KXT_A           MKPVKVKTP-A-GKEAE-------LVPEKVWALAPKG-RKG--VKIGLFKDPETGK-Y-FRHKLPDDYP
WP_015231698.1   LPPVKVKDPTT-GKEVT-------LTPIKVWTLAPKK-GKG--VKIGLFKSPETGK-Y-FRAK----VE
4R55_A           KKPVKVKTP-A-GKEAE-------LVPEKVWAL---G-R----VKIGLFKDPETGK-Y-FRHKLPDDYP
WP_011998075.1   -------CP-KCGAEVK--------EPIKTWVLAPKG-RKG--VIIGLFRCPN-GH-Y-FRAKVGEAPP
WP_075049584.1   -------CP-KCGAEVN--------EPIKTWQLAPKG-RKG--VIIGLFRCPN-GH-Y-FRAKVGEAPP
ADY01022.1       -------ME-K-EYEVECDGQSMKLKPVKVWMLAPKG-RRG--VIIGLFKCPG-GK-V-VRKAIGKAE-
KYH37633.1       -------CP-KCGTEVE--------KPIKEWEIQPKR-KKGPRVRISLFECPNCKT-R-FRTAT-RVA-
consensus/70%    ...lcsbs..p..bElE.......LpP.KVW.L.PKG.RKG..l.luLFKsPp.GK...hR+.lscs..

# Cren7ZnR
FINAL            --------------------------EEEEEEE---------EEEEEE-EEEE------EEEEEEEEEEEEE----EEEEE-----EEEE-----------------------------------------------------------
WP_012123536.1   MARKSKKKKDPF-V-CPKCGTRVSEPKKTWQLVSPLPDSKG-RITVTVMGSFECPNCGYKWRGVVSK--IKIGND-EVEVEARGKKKEIKDEVKR---K----------SEVQE----AR--EGEVIELDLDEIEK------EE
WP_048099394.1   MARKKQKEID---I-CPNCGTKVDKPIKTWNLVSPLPDSQG-RITITVMGSFVCPNCGYKWRGVVSK--IRAGGS-TVEVEGKKGVKK----------FGDEGKGDEGKKEDKK----KD--EGEIIELDLSDLDE------EE
WP_054837835.1   MARKKQKDST---T-CPNCGEENVKPTKTWQLVSPLPDSKG-RITITVMGTFDCPKCNHHWRGVVSK--IKAGGS-SVEVEGKKGVKK----------I----------GDEEEIDEKRE--EGEVIELDLSDIDE------DE
WP_054845496.1   MARKKQKDST---T-CPNCGETEVKPTKTWQLVSPLPDAKG-RITITVMGTFDCPKCGHHWRGVVSK--IKAGGS-SVEIEGKKGVKK----------I----------GEEEEAEEKKE--EGEVIELDLSDIDE------DE
AMD30850.1       MPRKSKDKSNDPFT-CPVCGVRVSAPERTWTLVSPIPDREG-RVTITIMGAFRCPN-GHTWKAVIKK--FKSGEEAQGE-------------------V----------EQPAE----KE--EGEVITLDISDIQNFN----DE
WP_009992346.1   MPKKKQIDPY---V-CPNCGTKAEKAQKTWQLVSPLPDSYG-RITITVMGSFVCPNCGHKWKSVVSK--IKAGGS-SVEIEGKKGVKK----------IESK-------DSEEK----AN--EGEVIELDLSDLDE------DE
WP_012711312.1   MPKKKQFDPY---V-CPNCGTKVEKAQKTWQLVSPLPDSYG-RITITVMGSFVCPNCGHKWKSVVSK--IKAGGS-SVEIEGKKGVKK----------IQSK-------DSEEK----AN--EGEVIELDLSDLDE------DE
WP_014512614.1   MPKKKQFDPY---V-CPNCGTKVEKAQKTWQLVSPLPDSYG-RITITVMGSFVCPNCGHKWKSVVSK--IKAGGS-SVEIEGKKGVKK----------IQSK-------DSEEK----AN--EAEVIELDLSDLDE------DE
WP_069281853.1   MPKKKQSDPY---V-CPNCGNRAEKPDKTWQLISPLPDSYG-RITITVMGSFTCSNCGNKWKAVVSK--IKAGGS-SVEIEGKKGVKK----------I----------ESKDSSQK-EE--EGEVIELDLSDLDE------DE
WP_075049443.1   MAKKKRDAT----I-CPKCGTKV-EPIKTWQLVSPMPDSKG-RITITIMGSFQCPNCGHKWRGVVSK--IKIGGG-EIEVKGKK-------------I----------KEPEPV---QE--EPEVIELDLDELDVS----DE
AFH42126.1       MVKKEKKPN----V-CPRCGTEVPNPTKTWNFVSPLPDDKG-RITITVMGSYKCPNCGYSWKAAISK--LKVGGG-EVEIDAGGKEKK----------I----------AGEAP----KQ--KPEEIELDLEDIIN------ED
WP_012021609.1   MPKKKQSEPD---V-CPNCGTKVEKPIKTWNLVSPLPDAYG-RITITVMGSYECPNCGHRWRAVVSK--IRAGGS-EVEVEGKKGVKK----------FG---------GEKKE----QRTDEGEIIELDLGDLDE------EE
WP_013776128.1   MPKRKQKEID---I-CPNCGTKVDKPVKTWNLVSPLPDAEG-RITITVMGSYVCPNCGYKWRGVVSK--IKAGGS-SVEIQGKNGTVKK---------IGD---------EKPEK----RD--EGEIIELDLSDLDE------EE
ADM27988.1       MARKQQEYN----I-CPRCGTKA-SPTRTWQLIAPLPDSQG-RITITIMGAYECPNCGYKWRGVVSK--MKVGSD-SIEIESGKKGIKL----------S----------SDEKSTS--SR--QGNIIELDLDEVLA------EE
EWG07001.1       MPKKQKDSN----V-CPNCGTVVDKPVKTWNLVSPLPDAYG-RITITVMGSYVCPNCGHRWRAVVSK--IKAGGD-SVEIEGKKGVKK----------FGEE-------KGKKE----ED--RGEIIELDISDLDE------EE
WP_009074728.1   MPKKQKEPD----V-CPNCGAKVDKPLKTWNLVSPLPDAYG-RITITVMGSYECPNCGHRWRAVVSK--IRAGGT-GVEVEGKKGVKK----------FG---------EQKAEQR--EE--EGEIIELDLGDIDE------EE
WP_010979229.1   MPKKKQTDPY---V-CPTCGTRVEKPIKTWQLVSPLPDAYG-RITITVMGSFECPN-GHKWKAVVSK--IRAGGS-SVEVEGKKGVKK----------IGEE-------KQEEE----EN--RGEVIELDLSDLDE------EE
WP_011839555.1   MPRKSSKQNKL--V-CPRCGSEDVEIVKQWQLVSPLPDKYG-RITITIMGVMQCRQCGYRWRGVISK--IKVGGS-GVEIEGKKGKKI----------V----------GEKEE----PR--RVKEIILDLEDLDL------EE
WP_013129045.1   MLNSSKKESSL--K-CPRCGSNNIEVVKSWQLVAPIPDAQG-RITVTMMGVVRCGNCGYKWKTTISK--IKVGGK-SVEIEGGGSKKV----------L----------EDESE----ER--EVKEIVLDISDILN------DK
WP_013143477.1   MPRKSSKQNKL--V-CPRCGSEDIEIVKQWQLVSPLPDKYG-RITITIMGVMQCRRCGYRWRGVISK--IKVGGS-GVEIEGKKGKKI----------V----------GEKEK----PR--RVKEIVLDLEDLDL------EE
WP_013266749.1   MPRKSKGKSGDPFT-CPVCGVRVTDPERTWTLVSPIPDREG-RVTITVMGAFRCPN-GHTWKAVIKK--FKSGEE-EGA-------------------GV---------PQTTE----KE--EGETITLDLSDIQNFNDEPPEE
WP_013737166.1   MPKKQRESD----V-CPNCGTKVDKPIKTWNLVSPLPDSYG-RITITVMGSYECPNCGHRWRAVVSK--IRAGGS-EVEVEGKKGVKK----------FGES-------KNQDK----QD--EGEIIELDLGDLDE------EE
AHC51640.1       MVRKKAVEAD---V-CPNCGKKAEKPVKTWQLVSPLPDAYG-RITITVMGSYECGNCGHKWKAVVSK--IKAGGS-SVEIEGKKGKKT----------I----------GEEKED---KR--EGEVIELDISDLDE------EE
WP_011278214.1   MVRKKAIEAD---V-CPNCGKKAEKPVKTWQLVSPLPDAYG-RITITVMGSYECGNCGHKWKAVVSK--IKAGGS-SVEIEGKKGKKT----------I----------GEEKED---KR--EGEVIELDISDLDE------EE
WP_055409861.1   MPRRKRDPY----V-CPQCGTRT-EPSKTWQLVSPFPDAKG-RITITVMGSFVCPECGHRWRSVVSK--IKVGGN-EVEVESGKGAKA----------AIKS-------DKEKE----ER--RGEIIEIDIDDIDE------ED
ESQ20288.1       MPRGGRRQADPF-T-CPVCGARVTEPERTWTVVSPIPDSKG-RVTITIMGSFRCPN-GHTWKAVIKK--FKSGGE-EGE-------------------G----------EGEGE----RE--PGEVITLDLSDIQGL-----DE
ESQ25549.1       MPRGSRRQADPF-T-CPVCGARVTEPERTWTVVSPIPDSKG-RVTITIMGSFRCPN-GHTWKAVIKK--FKSGGE-EGE-------------------G----------EGEGE----RE--PGEVITLDLSDIQGL-----DE
ESQ22624.1       MPRGGRRQADPF-T-CPVCGARVTEPERTWTVVSPIPDSKG-RVTITIMGSFRCPN-GHTWKAVIKK--FKSGGE-EEG------------------------------EGGGE----RE--PGEVITLDLSEIQGF-----DE
ESQ26964.1       MPRGGRRQADPF-T-CPVCGARVTEPERTWTVVSPIPDSKG-RVTITIMGSFRCPN-GHTWKAVIKK--FKSGGE-EGE------------------------------EGGGE----RE--PGEVITLDLSDIQDF-----DE
WP_058371372.1   MARKKRDPY----V-CPKCGTRT-EPVKTWQLVSPFPDSKG-RVTITVMGSFVCPNCGYRWRAVVSK--IKVGGS-EVELESGKGAKA----------SI---------TGEKE----ER--RGEVIEIDIDDID-------E
WP_012609105.1   MSKQPQL------K-CPKCDSNEVDVERTWQLTSPLPDTQG-RITITIMGVVKCRRCGYKWRGVVSK--LKVGGG-KVEIEGEKMSKV----------I----------EGGEE----PR--PPKEIILDIDDIMN------EE
WP_014766879.1   MSKQPQL------K-CPKCGSNEVDVEKTWQLTSPLPDTQG-RITITIMGVVKCRRCGYKWRGVVSK--LKVGGR-TVEIEGEKTGKV----------I----------ESGEE----PR--PPKEIVLDIEDIMN------EE
WP_015231931.1   MPRKKVEDPF---T-CPNCGTKVKEPDKTWTVVSPIPDKYG-RVSITIMGSFKCPNCGHSWKAVIKK--MKSEGG-EGE------------------------------QLPAN----NE--PGETIEIDLSDLDKV-----DE
WP_042666841.1   MSKQPQL------K-CPKCGSNEVDVERTWQLTSPLPDTQG-RITITIMGVVKCRRCGYKWRGVVSK--LKVGGG-KVEIEGEKMGKV----------I----------EGGEE----PR--PPKEIVLDIDDIMN------EE
WP_011822080.1   MPRKRDPY-----V-CPRCGTRT-EPAKTWQLVSPFPDSRG-RITITVMGSFVCPNCGHRWRAVVSK--IKVGGD-QVEVETGSGAKA----------RLAG-------EKEKE----ER--RGEVIEIDID----------E
WP_013562562.1   MSSRQV-------K-CPRCGSSEVDVEKTWQLVSPFPDSQG-RITVTVMGVMRCRSCGHRWRGPVSK--LKVGGR-SLEVEGEGGRKV----------F----------ESGEE----AR--PPKEIVIDLDDVLE------EE
WP_022542275.1   MARRKKQKDPY--I-CPSCGARAGEVEKTWTLVSPLPDRYG-RITITVMAVLTCRECGYSWKAPIQK--MKTGGD-EEA-------------------A----------ARGEEPP--RR--PGQVIELDLDEILK------GE
BAA81469.2       MARRKKQRDPY--V-CPSCGARAGEVEKTWTLVSPLPDKYG-RITITVMAVLSCRECGYSWKAPIKK--MKTGGD-EEA-------------------A----------SRGEEPP--KR--PGQVIELDLDEILG------EE
WP_014027473.1   MPRRQRDPT----I-CPRCGSKA-EPKKTWQLVSPFPDSKG-RITITIMASFECPNCGHKWRGALSK--LKVGGD-EMEIGGKKVKH-----------E----------QAAEE----KR--EGPVFEIDID----------E
WP_014736716.1   MSKQ-L-------T-CPRCGSTDVVVEKTWQLVSPLPDSAG-RITITVMGMVKCNRCGHRWRATISK--LKVGGS-SVEIEGGK--------------R----------FEEKE----ER--KPKEIVLDIDEILR------EE
KPV62726.1       MPKRSIKQN----L-CPSCGEVVKDPYKTWELIAPFPDKNT-RITVTIFGMFRCPSYGKSFKGMVGK--AKMGAE-GVE-------------------------------------------------------------I
consensus/70%    Ms++pp..s....s.CPpCGscs.cs.KTWpLVSPlPDsbG.RITITlMGsapCssCGa+W+uVlSK..lKsGGs.plElcu.........................p..pc....pc...uc.IpLDlsDl........-E

# Cren7 with N-terminal CdvA coiled-coil and middle 3-stranded beta-rich region
FINAL            ----HHHHHHHHHHH--HHHHHHHHHHHHHHHHHHHHHHHHHHHHHHHHHHHHHHHHHHHH---HHHHHHHHHHEE-----HHHHHHHHHHHHHHHHHHHHHHHHHHHHHHHHHHHH------------------------------------------------------------------------------------------------------------------------------------------------------------------------------------------------------
---------------------------EEE--------EEEEEEE--HHHHHHHHHHHHH--
OLD03897.1       MGLNDLTLEKLIGEINGYKEKMRAIHEDYGRASQELQDRINVLGYNLNEHLKETKDQIASTPQRLLTLETKYVTGEVNDGEYRQQREEFKGLLERNLHSIEEIRAMIATLSHIEARPLTPSEFKQAAAPR-TGPSADWNRPSAATESTPLLTPPQQN-PTVTA-TPQTALRFAP---------EPTSPTPSIPAYIAAPSGQQPTPTSIPNPQSANAPETEPTPQDRVVGPIFPMSP----------------
APVAAAPTDLGLIIDSPASEPIEASIDEATQTIPTVDIVETTGADEALMVKVVDDIPSVDASSVVVTDDSSALQPTIGTTDASAGIPSVASDLATVSLTPPTQFYKVVCPKCGADVPKPVKVWELKGGKSKKNVLIGLFQCQDCRVKFREALTREII
OLE70579.1       MVLNDLTLEKLISEINGYKEKMRTIYEEYGRASQDLQDRINVLGYTLNEHLKETKDQIGSTPQRLLNLETKYVTGEVSEQEYRGQREEFKGLLQRNLHSIEEIKDMIEVLTHIETRPLAPADFKQANPPA-PRTLIEEWSRNAVPQQAP--ATAQQT-PTPTP-TSPS-VKFAASSIVSERPWAPPTTPVAPQP--------SSNISTWTNPQTNNAPTSAP-----VSAPASVFTPPVQEKAQPVSAVATDPIAATPVTPLTQ-------ASTP---TVEDSFTTAPAQEYVEDV-AD-ALVVKVVDDMPILDASSAIATTE-NEIQPTIPGTETVPGIPS-
GPDMAQPSLQLPSQFYKVVCPKCGADVPKAAKVWELKGGKSKKNVLIGLFQCQDCRVKFREALTRDII
OLD55234.1       MGLNDLTIEKLITEINGYKEKMRTIHEEYGRASQDLQDRINVLGYTLNEHLKSTKDAMATTPQRLLNLEMKFVTGDVNESEYRAQRAEFKGLLENNLKSIEEIKQMIDTLGHIETKPLAPGEFKQTYPEN-KPATVSWVGIGALASSKSW-TPSQPQ-PLATP-AQAT-L--APSNVVEE--HAEPA-PVPALP--------QSNGTGSPIPETTNAP-SAP-----LNALAASTAP----------------VSAPVASSDI-------AASS---TLDESFNLAPSQDVAEDV-AD-ALVVKVVDDIPTLDASAALATTETSQVQPAIPSAEAVPGIQG-
SPDLSSVGLAPPSQFYKVVCPKCGADVPKPAKVWELKGGKSKKNVLIGLFQCQDCRVKFREALTREIL
OLE70912.1       MGLNDLTIEKLITEINGYKEKMRTIHEEYGRASQDLQDRINVLGYTLNEHLKSTKDAMATTPQRLLNLEMKFVTGDVNESEYRAQRAEFKGLLENNLKSIEEIKQMIDTLGHIETKPLAPGEFKQTYPEN-KPATVSWVGIGALASSKSW-TPSQPQ-PLATP-AQAT-L--APSNVVEE--HAEPA-PVPALP--------QSNGTGSPIPETTNAP-SAP-----LNALAAPTAP----------------VSAPVASSDI-------ASSS---TLDESFNLAPSQDVAEDV-AD-ALVVKVVDDIPTLDASAALATTETSQVQPAIPSAEAVPGIQG-
SPDLSSVGLAPPSQFYKVVCPKCGADVPKPAKVWELKGGKSKKNVLIGLFQCQDCRVKFREALTREIL
OLE68285.1       MVLNDLTLEKLVSEINGYKEKMRNIYEEYGRASQDLQDRINVLGYTLNEHLKETKDQIGSTPQRLLNLETKYVTGEVSEQEYRGQREEFKGLLQRNLHSIEEIKDMIEVLTHIETRPLASADFKQANPPA-PRTLIEEWARNAVPQQAP--TSTQQA-PVSTP-TSPS-IKFAASSIVSERPWAPPVTPAASQ-------------------TSYAPVTAP-----ISAPTPVTAPVQEQALPVSQITADPI-VAAPVAPLTQ-------ASTA---TVDDSFTATPAQEYVEDI-AD-ALVVKVVDDMPIVDASSAIATTE-SEIQPSIPGTETVPGIPS-
GPDMSQSSLQLPSQFYKVVCPKCGADVPKAAKVWELKGGKSKKNVLIGLFQCQDCRVKFREALTRDII
OLB70622.1       MGLNDLTIEKLITEINGYKEKMRTIHEEYGHASQDLQDRINVLGYTLNEHLKATKDQMATTPQRLLNLEMKFVTGDVNESEYRAQRAEFKGLLENNLKSIEEIKQMIDTLSHIETRPLVPQEFKQTYPENKPPASVSWVGIGALTAAKPW-TPTQPQ-PLATPITSPAQATLAP-NVVEE--HVEPASPAPALP--------QSNGTGSSTPETTNAPSTSP-----LNALAASSAP----------------LSTPVASADT-------ASST---TLDESLATGPAQDFAEDV-AD-ALVVKVVDDTPTLDASAAVTTTETSEVQPAIPGTEAIPGIQG-
SPDLSSVGVAPPSQFYKVVCPKCGADVPKPVKVWELKGGKSKKNVLIGLFQCQDCRVKFREALTREIL
OLE92038.1       MGLNDLTIEKLITEINGYKEKMRTIHEEYGRASQDLQDRINVLGYTLNEHLKSTKDAMATTPQRLLNLEMKFVTGDVNESEYRAQRAEFKGLLENNLKSIEEIKQMIDTLGHIETKPLAPGEFKQTYPENKPPASISWVGIGSLASAKPW-TPTQPQ-PLASP-TTPAQATLAPSNVVEE--HVEPTSPAPTLP--------ASNGTGSPTPETTNAPSTTP-----LNALAASTAP----------------VSAPVESSDI-------ASSS---TLDESFNLAPSQDVAEDI-AD-ALVVKVVDDIPTLDASAQVATTETSQVQPAIAGTEAIPGIQG-
SPDLSSVGLAPPSQFYKVVCPKCGADVPKPAKVWELKGGKSKKNVLIGLFQCQDCRVKFREALTREIL
OLE91878.1       MGLNDLTIEKLITEINGYKEKMRTIHEEYGRASSDLQDRINVLGYTLNEHLKQTKDQMATTPQRLLNLEMKFVTGDVNESEYRTQRTEFKGLLENNLKSIEEIKLMIDTLSHIETKPLAPGEFKQTYPEN-KPATVSWVGIGAIAQAKPS-LPIQPQWPAAQS-PAQA-VLTTPSNVVEA--NPEPTSPAPAIP--------QSNGTGSPTPETTNAP-SAP-----LNALAASTAP----------------VSAPIASSDI-------ASSS---ALDESFNVAPSQDVAEDV-AD-ALVVKVVDDIPTLDASAQIATTETSQVQPAIPGTEAVPGIQG-
SPDLSSVGLAPPSQFYKVVCPKCGADVPKPAKVWELKGGKSKKNVLIGLFQCQDCRVKFREALTREIL
```

```
OLD10777.1        -----------------------------------------------MNEHLKETKDQIGSTPQRLLNLETKYVTGEVSEQEYRGQREEFKGLLQRNLHSIEEIKDMIEVLTHIETRPLAPADFKQANPPA-PRTLIEEWSRNAVPQQAP--ATAQQT-PTPTP-TSPS-VKFAASSIVSERPWAPPTTPVAPQP---------SSNISTWTNPQTNNAPTSAP-----VSAPASVFTPPVQEKAQPVSAVATDPIAATPVTPLTQ-------ASTP---TVEDSFTTAPAQEYVEDV-AD-ALVVKVVDDMPILDASSAIATTE-NEIQPTIPGTETVPGIPS-
GPDMAQPSLQLPSQFYKVVCPKCGADVPKAAKVWELKGGKSKKNVLIGLFQCQDCRVKFREALTRDII
consensus/70%     MGLNDLTlEKLIoEINGYKEKMRTIHEEYGRASQDLQDRINVLGYTLNEHLKpTKDQhAoTPQRLLNLE.KaVTG-VNEuEYRuQR.EFKGLLEpNL+SIEEIKpMI-
TLuHIET+PLAPuEFKQshP.s.sssolsWhubuAlspupP..TPsQ.p.PhuTP.Tssu.l..AP.slVpE..asEPs.PsPulP.........pSNsTu.ssPbToNAP.oAP.....lsA.AusoAP................lusPVAssDb.......ASos...TlDESFssAPuQDhhEDV.AD.ALVVKVVDDhPoLDASuAlATTE.SblQPuIPGTEAVPGI.u.uPDLSoVuLsPPSQFYKVVCPKCGADVPKPAKVWELKGGKSKKNVLIGLFQCQDCRVKFREALTREIl

# Cren7 with 4 cysteines
FINAL            --------------------------EEE----------EEEEEEE------EEEEEEE------EEEEEEEEE----------------------
ALIGN            --------------------------EEEEE--------EEEEEEEE-----EEEEE---------EEEEEE----------------------
HMM                  ----------------EEEEE----EEEEE---------EEEHEEE------EEEEEEEE-----EEEEEEEEE---------------------
FREQ             ---------------------------------------EEEEEE------EEEEEE--------EEEEEEEE----------------------
PSSM             ---------------------------------------EEEEEEE------EE-EEEE---------EEEEEE----------------------
WP_012186112.1   ----------MGSMV-KCPYCGYEGNFRLIKQWRFRF--YIVKELECPKCHGVFNHYYGVNTQSNKVSEFVIRVKPK-SR---------------
WP_014767718.1   -------------MV-KCPFCGSEVNVAPIKEWRFRF--YIVNAYICPSCKGRFNYYHGVSSRGKE-SSFTIRVKPR-PKRIAAEG----------
WP_012608538.1   -------------MV-KCPFCGSEVNVAPIKEWRFRF--YIVNAYICPSCKGRFNYYHGVSPRGRE-SSFTIRVKPR-PKRVVAEG----------
WP_066795265.1   -------------MP-RCPYCGSE-DLTPIKTWRFRF--YEVTRYKCNKCGGIFQHYRNTTGRGK--PEFYIRIKPK-PSSTR------------
KUO92830.1       -------------MP-KCPYCGSE-GLTPIKSWRFRF--YDVTMYRCNRCGGEFNHYVNTTGKGK--PEYYVRIKPRLKRGVPSATSGSGHESVKD
AFA38724.1       -------------M--KCPYCGSE-KVEPVKSWEMPKMGYKVTHYRCKNCGGLFNHYAGKG------KEFVLRVGPR-RRG--------------
ABP51071.1       -------------M--KCPYCGSE-KVEPVKSWEMPKMGYKVTHYRCKNCGGLFNHYAGKG------KEFVLRVGAK-T----------------
AFA38036.1       -------------M--KCPYCGAE-NVEAVKRWEMPKMGYRVTHYRCKHCGGLFNHYVGKG------REFVLRVRPR-RIQGG------------
AET34264.1       MFDRVKSLCVKKLMPIKCPYCGSA-VIEAVKSWDMPKMGYHVTHYRCKTCGGLFNHYVGRG------REFVLRVGLR-KKTSA------------
AAL62651.1       -------------M--KCPYCGSE-NVEAVKSWEMPKMGFNVTHYRCKSCSGLFNHYVGRG------KEFVLRVGLR-RRG--------------
WP_013680511.1   -------------M--KCPYCGSE-NVEAVKSWDMPKMGYRVTHYRCRECGGLFNHYVGRG------REFTLRVGPR-KKASS------------
ABO08140.1       -------------M--KCPYCGSE-DVEAVKSWEMPKMGYRVTHYRCRRCGGLFNHYVGRG------REFVLRVGPR-RQVSQ------------
AAL63503.1       -------------M--KCPYCGSE-SVEAVKSWEMPRMGYRVTHYRCKNCGGLFNHYVGRG------KSSC-----------------------
consensus/70%    .............M..KCPYCGSE.slpslKoWch.b..YcVT+Y+CppCGGbFNHYhGpu......pEFhlRl.s+.pb................
```

[Back to Contents]

- **Supplementary S3: Bacterial chromo domains: Detailed overview of the sequences and domain architectures found in this study.**

# 90; CHROMO+CHROMO									
APR83931.1	Tudor-knot	CHROMO	Tudor-knot	193	A7982_09280	bacteria>proteobacteria>deltaproteobacteria	Minicystis rosea	Hypothetical protein A7982_09280 [Minicystis rosea]	1127362528
KXK45002.1	SP+Tudor-knot	CHROMO	Tudor-knot	164	UZ10_BCD003002415	bacteria>bacteroidetes	Bacteroidetes bacterium OLB10	RNA binding activity-knot of a chromodomain protein [Bacteroidetes bacterium OLB10]	1000283280
WP_054134435.1	-	TUDOR+TUDOR	-	161	-	bacteria>proteobacteria>alphaproteobacteria	Blastomonas sp. AAP25	hypothetical protein [Blastomonas sp. AAP25]	930030636
AKT38076.1	SP+Tudor-knot	CHROMO	Tudor-knot	159	CMC5_022180	bacteria>proteobacteria>deltaproteobacteria	Chondromyces crocatus	uncharacterized protein CMC5_022180 [Chondromyces crocatus]	908355501
WP_050430358.1	SP+Tudor-knot	CHROMO	Tudor-knot	159	-	bacteria>proteobacteria>deltaproteobacteria	Chondromyces crocatus	hypothetical protein [Chondromyces crocatus]	913450949
AGP33797.1	Tudor-knot	CHROMO	Tudor-knot	155	SCE1572_04365	bacteria>proteobacteria>deltaproteobacteria	Sorangium cellulosum So0157-2	hypothetical protein SCE1572_04365 [Sorangium cellulosum So0157-2]	521000794
CAN90800.1	Tudor-knot	CHROMO	Tudor-knot	155	sce0643	bacteria>proteobacteria>deltaproteobacteria	Sorangium cellulosum So ce56	hypothetical protein predicted by Glimmer/Critica [Sorangium cellulosum So ce56]	161159495
KYF52364.1	Tudor-knot	CHROMO	Tudor-knot	155	BE08_08860	bacteria>proteobacteria>deltaproteobacteria	Sorangium cellulosum	hypothetical protein BE08_08860 [Sorangium cellulosum]	1005168602
KYF84538.1	Tudor-knot	CHROMO	Tudor-knot	155	BE17_15850	bacteria>proteobacteria>deltaproteobacteria	Sorangium cellulosum	hypothetical protein BE17_15850 [Sorangium cellulosum]	1005215989
WP_012233278.1	Tudor-knot	CHROMO	Tudor-knot	155	-	bacteria>proteobacteria>deltaproteobacteria	Sorangium cellulosum	hypothetical protein [Sorangium cellulosum]	501190260
WP_020732868.1	Tudor-knot	CHROMO	Tudor-knot	155	-	bacteria>proteobacteria>deltaproteobacteria	Sorangium cellulosum	hypothetical protein [Sorangium cellulosum]	522819976
WP_061608144.1	Tudor-knot	CHROMO	Tudor-knot	155	-	bacteria>proteobacteria>deltaproteobacteria	Sorangium cellulosum	hypothetical protein [Sorangium cellulosum]	1005323104
WP_061619282.1	Tudor-knot	CHROMO	Tudor-knot	155	-	bacteria>proteobacteria>deltaproteobacteria	Sorangium cellulosum	hypothetical protein [Sorangium cellulosum]	1005335514
WP_061621897.1	Tudor-knot	CHROMO	Tudor-knot	155	-	bacteria>proteobacteria>deltaproteobacteria	Sorangium cellulosum	hypothetical protein [Sorangium cellulosum]	1005341005
EYF03907.1	Tudor-knot	CHROMO	Tudor-knot	154	CAP_5008	bacteria>proteobacteria>deltaproteobacteria	Chondromyces apiculatus DSM 436	Hypothetical protein CAP_5008 [Chondromyces apiculatus DSM 436]	599565795
WP_044245100.1	Tudor-knot	CHROMO	Tudor-knot	152	-	bacteria>proteobacteria>deltaproteobacteria	Chondromyces apiculatus	hypothetical protein [Chondromyces apiculatus]	763387965
WP_010472205.1	Tudor-knot	CHROMO	Tudor-knot	151	-	bacteria>cyanobacteria	Acaryochloris sp. CCMEE 5410	hypothetical protein [Acaryochloris sp. CCMEE 5410]	498158049
WP_049783751.1	Tudor-knot	CHROMO	Tudor-knot	151	-	bacteria>cyanobacteria	Acaryochloris marina	hypothetical protein [Acaryochloris marina]	908639731
WP_056105007.1	SP+DUF4537	TUDOR	DUF4537	151	-	bacteria>proteobacteria>alphaproteobacteria	Brevundimonas sp. Leaf363	hypothetical protein [Brevundimonas sp. Leaf363]	947440509
KPP82052.1	-	TUDOR+TUDOR	-	146	HLUCCA04_04820	bacteria>proteobacteria>alphaproteobacteria	Oceanicaulis sp. HLUCCA04	hypothetical protein HLUCCA04_04820 [Oceanicaulis sp. HLUCCA04]	938246608
ABW28876.1	Tudor-knot	CHROMO	Tudor-knot	144	AM1_3891	bacteria>cyanobacteria	Acaryochloris marina MBIC11017	conserved hypothetical protein [Acaryochloris marina MBIC11017]	158307259
SCX05627.1	SP+Agenet	TUDOR+TUDOR	Agenet	143	DSM25558_0797	bacteria>proteobacteria>alphaproteobacteria	Agrobacterium sp. DSM 25558	Agenet domain protein [Agrobacterium sp. DSM 25558]	1135564266
WP_045778338.1	Tudor-knot	CHROMO	Tudor-knot	143	-	bacteria>proteobacteria>gammaproteobacteria	Methylococcaceae bacterium Sn10-6	hypothetical protein [Methylococcaceae bacterium Sn10-6]	788040606
WP_051041233.1	SP+DUF4537	TUDOR+TUDOR	DUF4537	140	-	bacteria>proteobacteria>alphaproteobacteria	Blastomonas sp. AAP53	hypothetical protein [Blastomonas sp. AAP53]	916306187
EKD50851.1	SP+Tudor-knot+Tudor-knot	CHROMO	Tudor-knot+Tudor-knot	135	ACD_62C00415G0001	bacteria	uncultured bacterium	hypothetical protein ACD_62C00415G0001 [uncultured bacterium]	406910950
KFB72044.1	Tudor-knot+Tudor-knot	CHROMO	Tudor-knot+Tudor-knot	134	AW09_002769	bacteria>proteobacteria>betaproteobacteria	Candidatus Accumulibacter sp. BA-91	RNA binding activity-knot of a chromodomain protein [Candidatus Accumulibacter sp. BA-91]	668677806
WP_027844674.1	SP+Tudor-knot	CHROMO	Tudor-knot	134	-	bacteria>cyanobacteria	Mastigocoleus testarum	RNA-binding protein [Mastigocoleus testarum]	654351497
KQS54052.1	DUF4537	TUDOR	DUF4537	133	ASG17_13995	bacteria>proteobacteria>alphaproteobacteria	Brevundimonas sp. Leaf363	hypothetical protein ASG17_13995 [Brevundimonas sp. Leaf363]	945194479
WP_041739350.1	Tudor-knot	CHROMO	Tudor-knot	131	-	bacteria>cyanobacteria	Calothrix parietina	RNA-binding protein [Calothrix parietina]	754100980
ACC82964.1	SP+Tudor-knot	CHROMO	Tudor-knot	130	Npun_R4605	bacteria>cyanobacteria	Nostoc punctiforme PCC 73102	conserved hypothetical protein [Nostoc punctiforme PCC 73102]	186467163
WP_012410924.1	SP+Tudor-knot	CHROMO	Tudor-knot	130	-	bacteria>cyanobacteria	Nostoc punctiforme	hypothetical protein [Nostoc punctiforme]	501379358
OJW84507.1	Tudor-knot	CHROMO	Tudor-knot	129	BGO69_10970	bacteria>bacteroidetes	Bacteroidetes bacterium 46-16	hypothetical protein BGO69_10970 [Bacteroidetes bacterium 46-16]	1113422302
SFT96502.1	Agenet	TUDOR+CHROMO	Agenet	129	SAMN05518861_108153	bacteria>proteobacteria>alphaproteobacteria	Mesorhizobium sp. YR577	Agenet domain-containing protein [Mesorhizobium sp. YR577]	1099826738
WP_006511499.1	SP	CHROMO	-	129	-	bacteria>cyanobacteria	Xenococcus sp. PCC 7305	RNA-binding activity-knot of a chromodomain protein [Xenococcus sp. PCC 7305]	493558034
WP_052129381.1	SP+TUDOR	TUDOR+TUDOR	TUDOR	129	-	bacteria>proteobacteria>alphaproteobacteria	Sphingomonas sp. 35-24ZXX	hypothetical protein [Sphingomonas sp. 35-24ZXX]	917522964
WP_069067881.1	SP+Tudor-knot	CHROMO	Tudor-knot	129	-	bacteria>cyanobacteria	Nostoc sp. KVJ20	RNA-binding protein [Nostoc sp. KVJ20]	1060038569
WP_073641370.1	SP+Agenet	CHROMO	Agenet	129	-	bacteria>cyanobacteria	Nostoc calcicola	RNA-binding protein [Nostoc calcicola]	1121333471
AFZ35946.1	-	CHROMO	-	128	Sta7437_2407	bacteria>cyanobacteria	Stanieria cyanosphaera PCC 7437	hypothetical protein Sta7437_2407 [Stanieria cyanosphaera PCC 7437]	428270005
AOF99867.1	SMN	TUDOR+TUDOR	SMN	128	BSY18_323	bacteria>proteobacteria>alphaproteobacteria	Blastomonas sp. RAC04	hypothetical protein BSY18_323 [Blastomonas sp. RAC04]	1059506999
ESZ85545.1	SP	TUDOR+TUDOR	-	128	Q27BB25_18685	bacteria>proteobacteria>alphaproteobacteria	Blastomonas sp. CACIA14H2	hypothetical protein Q27BB25_18685 [Blastomonas sp. CACIA14H2]	563280945
KPF75756.1	SMN	TUDOR+TUDOR	SMN	128	IP68_07900	bacteria>proteobacteria>alphaproteobacteria	Blastomonas sp. AAP25	hypothetical protein IP68_07900 [Blastomonas sp. AAP25]	929693303
WP_015193614.1	-	CHROMO	-	128	-	bacteria>cyanobacteria	Stanieria cyanosphaera	hypothetical protein [Stanieria cyanosphaera]	505006512
WP_026817379.1	DUF4537	TUDOR+TUDOR	DUF4537	128	-	bacteria>proteobacteria>gammaproteobacteria	Arenimonas composti	hypothetical protein [Arenimonas composti]	652421938
WP_069051647.1	SMN	TUDOR+TUDOR	SMN	128	-	bacteria>proteobacteria>alphaproteobacteria	Blastomonas sp. RAC04	hypothetical protein [Blastomonas sp. RAC04]	1059971089
WP_067649684.1	Agenet	TUDOR+TUDOR	Agenet	126	-	bacteria>proteobacteria>alphaproteobacteria	Agrobacterium sp. R89-1	hypothetical protein [Agrobacterium sp. R89-1]	1056136832
WP_068185330.1	DUF4537	TUDOR+TUDOR	DUF4537	126	-	bacteria>proteobacteria>alphaproteobacteria	Rhizobiales bacterium CCH3-A5	hypothetical protein [Rhizobiales bacterium CCH3-A5]	1056762211
ACS60123.1	SP+Agenet	TUDOR+TUDOR	Agenet	124	Rleg_7114	bacteria>proteobacteria>alphaproteobacteria	Rhizobium leguminosarum bv. trifolii WSM1325	hypothetical protein Rleg_7114 (plasmid) [Rhizobium leguminosarum bv. trifolii WSM1325]	240862458
AHF87811.1	Agenet	TUDOR+TUDOR	Agenet	124	RLEG3_05985	bacteria>proteobacteria>alphaproteobacteria	Rhizobium leguminosarum bv. trifolii WSM1689	hypothetical protein RLEG3_05985 (plasmid) [Rhizobium leguminosarum bv. trifolii WSM1689]	573470931
SEH24350.1	Agenet	TUDOR+TUDOR	Agenet	124	SAMN03159407_2197	bacteria>proteobacteria>alphaproteobacteria	Rhizobium sp. NFR12	Agenet domain-containing protein [Rhizobium sp. NFR12]	1093287234
WP_003548409.1	SP+Agenet	TUDOR+TUDOR	Agenet	124	-	bacteria>proteobacteria>alphaproteobacteria	Rhizobium leguminosarum	hypothetical protein [Rhizobium leguminosarum]	489643969
WP_003553546.1	SP+Agenet	TUDOR+TUDOR	Agenet	124	-	bacteria>proteobacteria>alphaproteobacteria	Rhizobium leguminosarum	hypothetical protein [Rhizobium leguminosarum]	489649130
WP_007627274.1	SP+Agenet	TUDOR	Agenet	124	-	bacteria>proteobacteria>alphaproteobacteria	Rhizobium sp. CCGE 510	hypothetical protein [Rhizobium sp. CCGE 510]	494901229
WP_017991904.1	SP+Agenet	TUDOR+TUDOR	Agenet	124	-	bacteria>proteobacteria>alphaproteobacteria	Rhizobium leguminosarum	hypothetical protein [Rhizobium leguminosarum]	516617061
WP_017996816.1	SP+Agenet	TUDOR+CHROMO	Agenet	124	-	bacteria>proteobacteria>alphaproteobacteria	Rhizobium leguminosarum	hypothetical protein [Rhizobium leguminosarum]	516622029
WP_025397713.1	Agenet	TUDOR+TUDOR	Agenet	124	-	bacteria>proteobacteria>alphaproteobacteria	Rhizobium leguminosarum	hypothetical protein [Rhizobium leguminosarum]	644976959
WP_028743818.1	Agenet	TUDOR+TUDOR	Agenet	124	-	bacteria>proteobacteria>alphaproteobacteria	Rhizobium leguminosarum	hypothetical protein [Rhizobium leguminosarum]	655335629
WP_036351920.1	-	TUDOR+TUDOR	-	124	-	bacteria>proteobacteria>alphaproteobacteria	Microvirga sp. BSC39	hypothetical protein [Microvirga sp. BSC39]	738400228
WP_045020063.1	Agenet	TUDOR+TUDOR	Agenet	124	-	bacteria>proteobacteria>alphaproteobacteria	Agrobacterium arsenijevicii	hypothetical protein [Agrobacterium arsenijevicii]	769878315
WP_056339912.1	SP+Agenet	TUDOR+CHROMO	Agenet	124	-	bacteria>proteobacteria>alphaproteobacteria	Rhizobium sp. Root482	hypothetical protein [Rhizobium sp. Root482]	947677333
WP_062453349.1	Agenet	CHROMO	Agenet	124	-	bacteria>proteobacteria>alphaproteobacteria	Rhizobium sp. Leaf306	hypothetical protein [Rhizobium sp. Leaf306]	1011568078
WP_062579845.1	Agenet	TUDOR+TUDOR	Agenet	124	-	bacteria>proteobacteria>alphaproteobacteria	Rhizobium sp. Leaf321	hypothetical protein [Rhizobium sp. Leaf321]	1011756385
WP_062940488.1	SP+Agenet	TUDOR+TUDOR	Agenet	124	-	bacteria>proteobacteria>alphaproteobacteria	Rhizobium leguminosarum	hypothetical protein [Rhizobium leguminosarum]	1016098305
WP_064655152.1	SP+Agenet	TUDOR+TUDOR	Agenet	124	-	bacteria>proteobacteria>alphaproteobacteria	Rhizobium sp. WYCCWR10014	hypothetical protein [Rhizobium sp. WYCCWR10014]	1035869849
WP_077101993.1	Agenet	TUDOR+TUDOR	Agenet	124	-	bacteria>proteobacteria>alphaproteobacteria	Agrobacterium sp. DSM 25558	hypothetical protein [Agrobacterium sp. DSM 25558]	1143111705
WP_077119839.1	Agenet	TUDOR+TUDOR	Agenet	124	-	bacteria>proteobacteria>alphaproteobacteria	Agrobacterium sp. DSM 25559	hypothetical protein [Agrobacterium sp. DSM 25559]	1143138059
BAU66057.1	-	CHROMO	-	123	STA3757_34580	bacteria>cyanobacteria	Stanieria sp. NIES-3757	hypothetical protein STA3757_34580 [Stanieria sp. NIES-3757]	1003386233
OJY94922.1	DUF4537	TUDOR+TUDOR	DUF4537	123	BGP25_07115	bacteria>proteobacteria>gammaproteobacteria	Xanthomonadales bacterium 63-13	hypothetical protein BGP25_07115 [Xanthomonadales bacterium 63-13]	1113636127
SFN65226.1	DUF4537	TUDOR+TUDOR	DUF4537	123	SAMN05216289_1424	bacteria>proteobacteria>gammaproteobacteria	Dokdonella immobilis	Agenet domain-containing protein [Dokdonella immobilis]	1097982025
WP_035529614.1	-	TUDOR+TUDOR	-	123	-	bacteria>proteobacteria>alphaproteobacteria	Hoeflea sp. BAL378	hypothetical protein [Hoeflea sp. BAL378]	737556943
WP_036510638.1	DUF4537	TUDOR+TUDOR	DUF4537	123	-	bacteria>proteobacteria>alphaproteobacteria	Oceanicaulis sp. HL-87	hypothetical protein [Oceanicaulis sp. HL-87]	738600169
WP_071831297.1	Agenet	TUDOR+CHROMO	Agenet	123	-	bacteria>proteobacteria>alphaproteobacteria	Rhizobium	MULTISPECIES: hypothetical protein [Rhizobium]	1105247124
APH73035.1	DUF4265	TUDOR+TUDOR	DUF4265	122	BSQ44_17920	bacteria>proteobacteria>alphaproteobacteria	Mesorhizobium sp. B7	hypothetical protein BSQ44_17920 [Mesorhizobium sp. B7]	1111317021
CAK02999.1	-	CHROMO	-	122	pRL110057	bacteria>proteobacteria>alphaproteobacteria	Rhizobium leguminosarum bv. viciae 3841	hypothetical protein pRL110057 (plasmid) [Rhizobium leguminosarum bv. viciae 3841]	115259905
ODT48373.1	DUF4537	TUDOR+TUDOR	DUF4537	122	ABS74_13760	bacteria>proteobacteria>alphaproteobacteria	Pelagibacterium sp. SCN 63-126	hypothetical protein ABS74_13760 [Pelagibacterium sp. SCN 63-126]	1064044678
WP_072606506.1	DUF4265	TUDOR+TUDOR	DUF4265	122	-	bacteria>proteobacteria>alphaproteobacteria	Mesorhizobium sp. B7	hypothetical protein [Mesorhizobium sp. B7]	1119687674
BAE52056.1	Agenet	CHROMO	Agenet	121	amb3252	bacteria>proteobacteria>alphaproteobacteria	Magnetospirillum magneticum AMB-1	hypothetical protein amb3252 [Magnetospirillum magneticum AMB-1]	82947192
OIP36437.1	SP	TUDOR+TUDOR	-	121	AUK47_15095	bacteria>proteobacteria>deltaproteobacteria	Deltaproteobacteria bacterium CG2_30_63_29	hypothetical protein AUK47_15095 [Deltaproteobacteria bacterium CG2_30_63_29]	1101184449
WP_011385617.1	Agenet	CHROMO	Agenet	121	-	bacteria>proteobacteria>alphaproteobacteria	Magnetospirillum magneticum	hypothetical protein [Magnetospirillum magneticum]	499704883
WP_068489443.1	Tudor-knot+Agenet	CHROMO	Tudor-knot+Agenet	121	-	bacteria>proteobacteria>alphaproteobacteria	Magnetospirillum marisnigri	hypothetical protein [Magnetospirillum marisnigri]	1057088509
AOO81166.1	DUF4537	TUDOR	DUF4537	120	BHK69_12455	bacteria>proteobacteria>alphaproteobacteria	Bosea vaviloviae	hypothetical protein BHK69_12455 [Bosea vaviloviae]	1069441779
SFJ66088.1	DUF4537	TUDOR	DUF4537	120	SAMN05216304_11158	bacteria>proteobacteria>alphaproteobacteria	Bosea sp. OK403	hypothetical protein SAMN05216304_11158 [Bosea sp. OK403]	1099676243
WP_056707486.1	-	TUDOR+TUDOR	-	120	-	bacteria>proteobacteria>alphaproteobacteria	Bosea sp. Leaf344	hypothetical protein [Bosea sp. Leaf344]	948048458
WP_069690380.1	DUF4537	TUDOR	DUF4537	120	-	bacteria>proteobacteria>alphaproteobacteria	Bosea vaviloviae	hypothetical protein [Bosea vaviloviae]	1069638007
AFZ01055.1	Tudor-knot	CHROMO	Tudor-knot	119	Cal6303_2026	bacteria>cyanobacteria	Calothrix sp. PCC 6303	hypothetical protein Cal6303_2026 [Calothrix sp. PCC 6303]	428235265
WP_056820449.1	Agenet	TUDOR+TUDOR	Agenet	118	-	bacteria>proteobacteria>alphaproteobacteria	Rhizobium sp. Root708	hypothetical protein [Rhizobium sp. Root708]	948162267
WP_068959266.1	Agenet	TUDOR+CHROMO	Agenet	116	-	bacteria>proteobacteria>alphaproteobacteria	Pararhizobium polonicum	hypothetical protein [Pararhizobium polonicum]	1057684514
WP_066274389.1	-	TUDOR+TUDOR	-	115	-	bacteria>proteobacteria>alphaproteobacteria	Blastomonas sp. CCH1-A6	hypothetical protein [Blastomonas sp. CCH1-A6]	1054475043
EKD41860.1	SP	TUDOR+TUDOR	-	108	ACD_73C00486G0002	bacteria	uncultured bacterium	hypothetical protein ACD_73C00486G0002, partial [uncultured bacterium]	406898151
ODU53995.1	-	TUDOR+TUDOR	-	107	ABT09_00600	bacteria	bacterium SCN 57-13	hypothetical protein ABT09_00600 [bacterium SCN 57-13]	1064155955
OJU62241.1	-	TUDOR+TUDOR	-	107	BGO01_06965	bacteria>armatimonadetes	Armatimonadetes bacterium 55-13	hypothetical protein BGO01_06965 [Armatimonadetes bacterium 55-13]	1113191945
# 2; CHROMO+CHROMO+CHROMO									
WP_068362540.1	-	CHROMO+CHROMO	-	220	-	bacteria>proteobacteria>alphaproteobacteria	Jannaschia sp. EhC01	hypothetical protein [Jannaschia sp. EhC01]	1056949161
OAN72858.1	-	CHROMO+CHROMO	-	200	A8B78_19315	bacteria>proteobacteria>alphaproteobacteria	Jannaschia sp. EhC01	hypothetical protein A8B78_19315 [Jannaschia sp. EhC01]	1032100325
# 40; CHROMO+CHROMO									
AFZ02755.1	Tudor-knot+Tudor-knot	CHROMO	Tudor-knot+Tudor-knot	445	Cal6303_3838	bacteria>cyanobacteria	Calothrix sp. PCC 6303	hypothetical protein Cal6303_3838 [Calothrix sp. PCC 6303]	428236965
WP_015199380.1	Tudor-knot+Tudor-knot	CHROMO	Tudor-knot+Tudor-knot	445	-	bacteria>cyanobacteria	Calothrix parietina	hypothetical protein, partial [Calothrix parietina]	505012278
EKE98902.1	Tudor-knot	CHROMO	Tudor-knot	443	FDUTEX481_03534	bacteria>cyanobacteria	Tolypothrix sp. PCC 7601	hypothetical protein FDUTEX481_03534 [Tolypothrix sp. PCC 7601]	407267399
AFY43036.1	Tudor-knot+Tudor-knot	CHROMO	Tudor-knot+Tudor-knot	439	Nos7107_2428	bacteria>cyanobacteria	Nostoc sp. PCC 7107	hypothetical protein Nos7107_2428 [Nostoc sp. PCC 7107]	427360314
WP_015113251.1	Tudor-knot+Tudor-knot	CHROMO	Tudor-knot+Tudor-knot	439	-	bacteria>cyanobacteria	Nostoc sp. PCC 7107	hypothetical protein [Nostoc sp. PCC 7107]	504926149
WP_017713524.1	Tudor-knot+Tudor-knot	CHROMO	Tudor-knot+Tudor-knot	439	-	bacteria>cyanobacteria	Prochlorothrix hollandica	hypothetical protein [Prochlorothrix hollandica]	516316830
OCQ95691.1	Tudor-knot	CHROMO	Tudor-knot	438	BCD64_09680	bacteria>cyanobacteria	Nostoc sp. MBR 210	hypothetical protein BCD64_09680 [Nostoc sp. MBR 210]	1049317479
WP_062291351.1	Tudor-knot+Tudor-knot	CHROMO	Tudor-knot+Tudor-knot	438	-	bacteria>cyanobacteria	Nostoc piscinale	hypothetical protein [Nostoc piscinale]	1011378586
ACC80130.1	Tudor-knot+Tudor-knot	CHROMO	Tudor-knot+Tudor-knot	436	Npun_R1423	bacteria>cyanobacteria	Nostoc punctiforme PCC 73102	TUDOR [Nostoc punctiforme PCC 73102]	186464329
WP_012408151.1	Tudor-knot+Tudor-knot	CHROMO	Tudor-knot+Tudor-knot	436	-	bacteria>cyanobacteria	Nostoc punctiforme	hypothetical protein, partial [Nostoc punctiforme]	501376585
WP_054466600.1	Agenet	CHROMO	Agenet	436	-	bacteria>cyanobacteria	Planktothricoides sp. SR001	hypothetical protein [Planktothricoides sp. SR001]	935602156
AFY31860.1	Tudor-knot+Tudor-knot	CHROMO	Tudor-knot+Tudor-knot	435	Cal7507_1393	bacteria>cyanobacteria	Calothrix sp. PCC 7507	hypothetical protein Cal7507_1393 [Calothrix sp. PCC 7507]	427349136
CUR11548.1	Tudor-knot+Tudor-knot	CHROMO	Tudor-knot+Tudor-knot	435	PL11201220008	bacteria>cyanobacteria	Planktothrix sp. PCC 11201	TUDOR [Planktothrix sp. PCC 11201]	1108755581
SKB11890.1	Tudor-knot+Tudor-knot	CHROMO	Tudor-knot+Tudor-knot	435	PL11201_220008	bacteria>cyanobacteria	Planktothrix sp. PCC 11201	TUDOR [Planktothrix sp. PCC 11201]	1152218607
WP_015127680.1	Tudor-knot+Tudor-knot	CHROMO	Tudor-knot+Tudor-knot	435	-	bacteria>cyanobacteria	Calothrix sp. PCC 7507	hypothetical protein [Calothrix sp. PCC 7507]	504940578
WP_072718577.1	Tudor-knot+Tudor-knot	CHROMO	Tudor-knot+Tudor-knot	435	-	bacteria>cyanobacteria	Planktothrix tepida	hypothetical protein [Planktothrix tepida]	1119826200
ABA22074.1	Tudor-knot	CHROMO	Tudor-knot	434	Ava_2459	bacteria>cyanobacteria	Anabaena variabilis ATCC 29413	Tudor [Anabaena variabilis ATCC 29413]	75702398
AFZ60423.1	MBT+Tudor-knot	CHROMO	MBT+Tudor-knot	434	Anacy_5085	bacteria>cyanobacteria	Anabaena cylindrica PCC 7122	Agenet domain-containing protein [Anabaena cylindrica PCC 7122]	428681657
BAB76292.1	Tudor-knot	CHROMO	Tudor-knot	434	all4593	bacteria>cyanobacteria	Nostoc sp. PCC 7120	all4593 [Nostoc sp. PCC 7120]	17133730
OBQ05004.1	Tudor-knot	CHROMO	Tudor-knot	434	AN489_20525	bacteria>cyanobacteria	Anabaena sp. 39858	hypothetical protein AN489_20525 [Anabaena sp. 39858]	1039159574
WP_010998724.1	Tudor-knot	CHROMO	Tudor-knot	434	-	bacteria>cyanobacteria	Nostoc sp. PCC 7120	hypothetical protein, partial [Nostoc sp. PCC 7120]	499307949
WP_015217039.1	MBT+Tudor-knot	CHROMO	MBT+Tudor-knot	434	-	bacteria>cyanobacteria	Anabaena cylindrica	hypothetical protein, partial [Anabaena cylindrica]	505029937
WP_019488990.1	Tudor-knot	CHROMO	Tudor-knot	434	-	bacteria>cyanobacteria	Calothrix sp. PCC 7103	Tudor protein, partial [Calothrix sp. PCC 7103]	518318783
WP_045873047.1	Tudor-knot	CHROMO	Tudor-knot	434	-	bacteria>cyanobacteria	Tolypothrix sp. PCC 7601	Tudor protein [Tolypothrix sp. PCC 7601]	797212106
WP_069069251.1	Tudor-knot+Tudor-knot	CHROMO	Tudor-knot+Tudor-knot	434	-	bacteria>cyanobacteria	Nostoc sp. KVJ20	hypothetical protein [Nostoc sp. KVJ20]	1060040568
WP_072619455.1	Tudor-knot	CHROMO	Tudor-knot	434	-	bacteria>cyanobacteria	Spirulina major	hypothetical protein [Spirulina major]	1119701363
WP_073618010.1	Tudor-knot+Tudor-knot	CHROMO	Tudor-knot+Tudor-knot	434	-	bacteria>cyanobacteria	Calothrix sp. HK-06	hypothetical protein [Calothrix sp. HK-06]	1121306502
WP_073639202.1	Tudor-knot+Tudor-knot	CHROMO	Tudor-knot+Tudor-knot	434	-	bacteria>cyanobacteria	Nostoc calcicola	hypothetical protein [Nostoc calcicola]	1121331262
AKG20696.1	Tudor-knot+Tudor-knot	CHROMO	Tudor-knot+Tudor-knot	432	IJ00_04695	bacteria>cyanobacteria	Calothrix sp. 336/3	Tudor protein [Calothrix sp. 336/3]	817926208
WP_035150549.1	Tudor-knot+Tudor-knot	CHROMO	Tudor-knot+Tudor-knot	432	-	bacteria>cyanobacteria	Calothrix sp. 336/3	Tudor protein [Calothrix sp. 336/3]	737164107
AFY83281.1	Tudor-knot+Tudor-knot	CHROMO	Tudor-knot+Tudor-knot	431	Oscil6304_3721	bacteria>cyanobacteria	Oscillatoria acuminata PCC 6304	RNA binding activity-knot of a chromodomain protein [Oscillatoria acuminata PCC 6304]	428002438
WP_017747698.1	Agenet+Tudor-knot	CHROMO	Agenet+Tudor-knot	429	-	bacteria>cyanobacteria	Scytonema hofmannii	Tudor protein, partial [Scytonema hofmannii]	516357665
BAT53591.1	Tudor-knot+Tudor-knot	CHROMO+CHROMO	Tudor-knot+Tudor-knot	427	NOS3756_25520	bacteria>cyanobacteria	Nostoc sp. NIES-3756	hypothetical protein NOS3756_25520 [Nostoc sp. NIES-3756]	953510903
WP_038077790.1	Agenet+Tudor-knot	CHROMO	Agenet+Tudor-knot	427	-	bacteria>cyanobacteria	Tolypothrix bouteillei	Tudor protein [Tolypothrix bouteillei]	740236636
WP_067768867.1	Tudor-knot+Tudor-knot	CHROMO+CHROMO	Tudor-knot+Tudor-knot	427	-	bacteria>cyanobacteria	Nostoc sp. NIES-3756	hypothetical protein [Nostoc sp. NIES-3756]	1056314049
WP_044197418.1	Tudor-knot+Tudor-knot	CHROMO	Tudor-knot+Tudor-knot	425	-	bacteria>cyanobacteria	Oscillatoria acuminata	Tudor protein [Oscillatoria acuminata]	763339097
WP_009787970.1	Tudor-knot	CHROMO	Tudor-knot	378	-	bacteria>cyanobacteria	Lyngbya sp. PCC 8106	Tudor [Lyngbya sp. PCC 8106]	497473772
WP_023067754.1	Tudor-knot	CHROMO	Tudor-knot	378	-	bacteria>cyanobacteria	Lyngbya aestuarii	RNA-binding activity-knot of a chromodomain protein [Lyngbya aestuarii]	553733104
WP_046279647.1	Tudor-knot	CHROMO	Tudor-knot	378	-	bacteria>cyanobacteria	Limnoraphis robusta	Tudor [Limnoraphis robusta]	808797251
AAT41893.1	Tudor-knot	CHROMO	Tudor-knot	365	-	bacteria>cyanobacteria	Fremyella diplosiphon Fd33	hypothetical protein, partial [Fremyella diplosiphon Fd33]	48374186
# 20; CASPASE+CHROMO+CHROMO									
EKD80991.1	SP+Agenet	CASPASE+CHROMO	Agenet	408	ACD_39C02055G0002	bacteria	uncultured bacterium	hypothetical protein ACD_39C02055G0002 [uncultured bacterium]	406950831
OGK06963.1	Tudor-knot	CASPASE+CHROMO	Tudor-knot	393	A2W80_19330	bacteria	Candidatus Riflebacteria bacterium GWC2_50_8	hypothetical protein A2W80_19330 [Candidatus Riflebacteria bacterium GWC2_50_8]	1083992505
ADG68645.1	-	CASPASE+CHROMO	-	388	Plim_2823	bacteria>planctomycetes	Planctopirus limnophila DSM 3776	hypothetical protein Plim_2823 [Planctopirus limnophila DSM 3776]	296015406
WP_013111076.1	-	CASPASE+CHROMO	-	388	-	bacteria>planctomycetes	Planctopirus limnophila	hypothetical protein [Planctopirus limnophila]	502876100
WP_068848381.1	-	CASPASE+CHROMO	-	381	-	bacteria>planctomycetes	Planctopirus sp. JC280	hypothetical protein [Planctopirus sp. JC280]	1057554678
OAI53464.1	-	CASPASE+CHROMO	-	368	AYO47_00875	bacteria>planctomycetes	Planctomyces sp. SCGC AG-212-M04	hypothetical protein AYO47_00875 [Planctomyces sp. SCGC AG-212-M04]	1028423886
WP_028527108.1	Tudor-knot	CASPASE+CHROMO	Tudor-knot	367	-	bacteria>bacteroidetes	Runella limosa	hypothetical protein [Runella limosa]	655078984
AEI49153.1	-	CASPASE+CHROMO	-	365	Runsl_2756	bacteria>bacteroidetes	Runella slithyformis DSM 19594	hypothetical protein Runsl_2756 [Runella slithyformis DSM 19594]	336306051
WP_013928462.1	-	CASPASE+CHROMO	-	365	-	bacteria>bacteroidetes	Runella slithyformis	hypothetical protein [Runella slithyformis]	503694386
AFK04103.1	SAWADEE	CASPASE+CHROMO	SAWADEE	361	Emtol_2970	bacteria>bacteroidetes	Emticicia oligotrophica DSM 17448	hypothetical protein Emtol_2970 [Emticicia oligotrophica DSM 17448]	387856006
WP_015029797.1	SAWADEE	CASPASE+CHROMO	SAWADEE	361	-	bacteria>bacteroidetes	Emticicia oligotrophica	hypothetical protein [Emticicia oligotrophica]	504842695
WP_064196371.1	Tudor-knot	CASPASE+CHROMO	Tudor-knot	361	-	bacteria>bacteroidetes	Emticicia sp. MM	hypothetical protein [Emticicia sp. MM]	1032340001
WP_029246835.1	Peptidase_C13+Tudor-knot+Tudor-knot	CASPASE+CHROMO	Peptidase_C13+Tudor-knot+Tudor-knot	359	-	bacteria>planctomycetes	Schlesneria paludicola	hypothetical protein [Schlesneria paludicola]	656314444
AFZ17430.1	Agenet	CASPASE+CHROMO	Agenet	353	Mic7113_1554	bacteria>cyanobacteria	Microcoleus sp. PCC 7113	hypothetical protein Mic7113_1554 [Microcoleus sp. PCC 7113]	428251471
WP_015181586.1	Agenet	CASPASE+CHROMO	Agenet	353	-	bacteria>cyanobacteria	Microcoleus sp. PCC 7113	hypothetical protein [Microcoleus sp. PCC 7113]	504994484
WP_015209299.1	Agenet	CASPASE+CHROMO	Agenet	353	-	bacteria>cyanobacteria	Cylindrospermum stagnale	hypothetical protein [Cylindrospermum stagnale]	505022197
OCQ93872.1	Peptidase_C14+Agenet+Tudor-knot	CASPASE+CHROMO	Peptidase_C14+Agenet+Tudor-knot	352	BCR12_09940	bacteria>cyanobacteria	Limnothrix sp. P13C2	hypothetical protein BCR12_09940 [Limnothrix sp. P13C2]	1049315642
OKY71338.1	Peptidase_C14+Agenet+Agenet	CASPASE+CHROMO	Peptidase_C14+Agenet+Agenet	352	BJG00_11880	bacteria>cyanobacteria	Limnothrix sp. CACIAM 69d	hypothetical protein BJG00_11880 [Limnothrix sp. CACIAM 69d]	1124125944
KPV50666.1	Tudor-knot+Tudor-knot	CASPASE+CHROMO	Tudor-knot+Tudor-knot	351	SE17_25570	bacteria>chloroflexi	Kouleothrix aurantiaca	hypothetical protein SE17_25570 [Kouleothrix aurantiaca]	939555088
WP_069967412.1	Agenet+Tudor-knot	CASPASE+CHROMO	Agenet+Tudor-knot	350	-	bacteria>cyanobacteria	Desertifilum sp. IPPAS B-1220	hypothetical protein [Desertifilum sp. IPPAS B-1220]	1072777549
# 13; CHROMO									
WP_041227136.1	-	-	-	313	-	bacteria>deinococcus-thermus>deinococci	Deinococcus deserti	hypothetical protein [Deinococcus deserti]	752555904
WP_012694986.1	-	-	-	302	-	bacteria>deinococcus-thermus>deinococci	Deinococcus deserti	hypothetical protein [Deinococcus deserti]	502017864
ACO45954.1	-	-	-	296	Deide_10620	bacteria>deinococcus-thermus>deinococci	Deinococcus deserti VCD115	Hypothetical protein, precursor [Deinococcus deserti VCD115]	226317958
WP_034406875.1	-	-	-	286	-	bacteria>deinococcus-thermus>deinococci	Deinococcus	MULTISPECIES: hypothetical protein [Deinococcus]	736382464

ACO46634.1	-	-	-	268	Deide_16620	bacteria>deinococcus-thermus>deinococci	Deinococcus deserti VCD115	Hypothetical protein, precursor [Deinococcus deserti VCD115]	226318638
WP_041227204.1	-	-	-	229	-	bacteria>deinococcus-thermus>deinococci	Deinococcus deserti	hypothetical protein [Deinococcus deserti]	752555972
AHX26555.1	-	-	-	210	Deide_1p01055	bacteria>deinococcus-thermus>deinococci	Deinococcus deserti VCD115	hypothetical protein, precursor (plasmid) [Deinococcus deserti VCD115]	613467780
WP_041227723.1	-	-	-	197	-	bacteria>deinococcus-thermus>deinococci	Deinococcus deserti	hypothetical protein [Deinococcus deserti]	752556491
WP_039682170.1	-	-	-	143	-	bacteria>deinococcus-thermus>deinococci	Deinococcus swuensis	hypothetical protein [Deinococcus swuensis]	746724846
WP_019008147.1	-	RNaseH	-	142	-	bacteria>deinococcus-thermus>deinococci	Deinococcus aquatilis	hypothetical protein [Deinococcus aquatilis]	517837939
WP_056304101.1	SP	-	-	142	-	bacteria>deinococcus-thermus>deinococci	Deinococcus sp. Leaf326	hypothetical protein [Deinococcus sp. Leaf326]	947641180
WP_064015324.1	SP	-	-	142	-	bacteria>deinococcus-thermus>deinococci	Deinococcus puniceus	hypothetical protein [Deinococcus puniceus]	1028846360
WP_029484637.1	-	-	-	133	-	bacteria>deinococcus-thermus>deinococci	Deinococcus marmoris	hypothetical protein [Deinococcus marmoris]	657682889
# 13; CHROMO									
BAI71243.1	Agenet	CHROMO	Agenet	234	AZL_006050	bacteria>proteobacteria>alphaproteobacteria	Azospirillum sp. B510	hypothetical protein AZL_006050 [Azospirillum sp. B510]	288909754
WP_012973229.1	Agenet	CHROMO	Agenet	234	-	bacteria>proteobacteria>alphaproteobacteria	Azospirillum lipoferum	hypothetical protein [Azospirillum lipoferum]	502738245
ANC90766.1	Agenet	CHROMO	Agenet	231	A6A40_01965	bacteria>proteobacteria>alphaproteobacteria	Azospirillum humicireducens	hypothetical protein A6A40_01965 [Azospirillum humicireducens]	1026294427
CBS85858.1	Agenet	CHROMO	Agenet	231	AZOLI_0485	bacteria>proteobacteria>alphaproteobacteria	Azospirillum lipoferum 4B	exported protein of unknown function [Azospirillum lipoferum 4B]	357423015
WP_014246909.1	Agenet	CHROMO	Agenet	231	-	bacteria>proteobacteria>alphaproteobacteria	Azospirillum lipoferum	hypothetical protein [Azospirillum lipoferum]	504012915
WP_063633875.1	Agenet	CHROMO	Agenet	231	-	bacteria>proteobacteria>alphaproteobacteria	Azospirillum humicireducens	hypothetical protein [Azospirillum humicireducens]	1027698137
ALG69790.1	Agenet	CHROMO	Agenet	230	AL072_01355	bacteria>proteobacteria>alphaproteobacteria	Azospirillum thiophilum	hypothetical protein AL072_01355 [Azospirillum thiophilum]	933519471
WP_045581825.1	Agenet	CHROMO	Agenet	230	-	bacteria>proteobacteria>alphaproteobacteria	Azospirillum thiophilum	hypothetical protein [Azospirillum thiophilum]	780530955
WP_042694076.1	Agenet	TUDOR	Agenet	229	-	bacteria>proteobacteria>alphaproteobacteria	Azospirillum sp. B506	hypothetical protein [Azospirillum sp. B506]	757139831
CCG08965.1	SP+Agenet	CHROMO	Agenet	224	RSPPHO_02339	bacteria>proteobacteria>alphaproteobacteria	Pararhodospirillum photometricum DSM 122	Putative uncharacterized protein [Pararhodospirillum photometricum DSM 122]	378403849
WP_068491020.1	-	CHROMO	-	218	-	bacteria>proteobacteria>alphaproteobacteria	Magnetospirillum marisnigri	hypothetical protein [Magnetospirillum marisnigri]	1057090087
WP_041795432.1	SP+Agenet	CHROMO	Agenet	217	-	bacteria>proteobacteria>alphaproteobacteria	Pararhodospirillum photometricum	hypothetical protein [Pararhodospirillum photometricum]	754176812
KJS42670.1	Agenet	CHROMO	Agenet	213	VR70_03920	bacteria>proteobacteria>alphaproteobacteria	Rhodospirillaceae bacterium BRH_c57	hypothetical protein VR70_03920 [Rhodospirillaceae bacterium BRH_c57]	780841173
# 10; CHROMO									
AGP41876.1	Tudor-knot	CHROMO	Tudor-knot	157	SCE1572_49730	bacteria>proteobacteria>deltaproteobacteria	Sorangium cellulosum So0157-2	hypothetical protein SCE1572_49730 [Sorangium cellulosum So0157-2]	521008873
KYG08725.1	Tudor-knot	CHROMO	Tudor-knot	157	BE21_21980	bacteria>proteobacteria>deltaproteobacteria	Sorangium cellulosum	hypothetical protein BE21_21980 [Sorangium cellulosum]	1005250411
WP_020741776.1	Tudor-knot	CHROMO	Tudor-knot	157	-	bacteria>proteobacteria>deltaproteobacteria	Sorangium cellulosum	hypothetical protein [Sorangium cellulosum]	522829119
WP_061629242.1	-	CHROMO	-	157	-	bacteria>proteobacteria>deltaproteobacteria	Sorangium cellulosum	hypothetical protein [Sorangium cellulosum]	1005348350
CAN98738.1	-	CHROMO	-	145	sce8568	bacteria>proteobacteria>deltaproteobacteria	Sorangium cellulosum So ce56	hypothetical protein predicted by Glimmer/Critica [Sorangium cellulosum So ce56]	161167433
KYF56388.1	-	CHROMO	-	145	BE08_29725	bacteria>proteobacteria>deltaproteobacteria	Sorangium cellulosum	hypothetical protein BE08_29725 [Sorangium cellulosum]	1005176358
WP_012241177.1	-	CHROMO	-	145	-	bacteria>proteobacteria>deltaproteobacteria	Sorangium cellulosum	hypothetical protein [Sorangium cellulosum]	501198159
WP_061612875.1	-	CHROMO	-	145	-	bacteria>proteobacteria>deltaproteobacteria	Sorangium cellulosum	hypothetical protein [Sorangium cellulosum]	1005327835
KYF80105.1	Tudor-knot	CHROMO	Tudor-knot	144	BE17_41855	bacteria>proteobacteria>deltaproteobacteria	Sorangium cellulosum	hypothetical protein BE17_41855 [Sorangium cellulosum]	1005209533
OJY16907.1	Agenet	CHROMO	Agenet	127	BGO98_12040	bacteria>proteobacteria>deltaproteobacteria	Myxococcales bacterium 68-20	hypothetical protein BGO98_12040 [Myxococcales bacterium 68-20]	1113556887
# 2; beta-sandwich_domain+CHROMO+CHROMO+CHROMO+CHROMO+CHROMO+CHROMO									
AHZ86290.1	-	-	-	530	EP01_15305	bacteria>proteobacteria>deltaproteobacteria	Bdellovibrio bacteriovorus	hypothetical protein EP01_15305 [Bdellovibrio bacteriovorus]	635303551
AFY00688.1	-	-	-	520	Bdt_0988	bacteria>proteobacteria>deltaproteobacteria	Bdellovibrio bacteriovorus str. Tiberius	hypothetical protein Bdt_0988 [Bdellovibrio bacteriovorus str. Tiberius]	425859652
# 1; beta-sandwich_domain+CHROMO+CHROMO+CHROMO+CHROMO+CHROMO									
CAE78975.1	-	-	-	514	Bd1045	bacteria>proteobacteria>deltaproteobacteria	Bdellovibrio bacteriovorus HD100	hypothetical protein predicted by Glimmer/Critica [Bdellovibrio bacteriovorus HD100]	39575134
AFY00687.1	-	-	-	487	Bdt_0987	bacteria>proteobacteria>deltaproteobacteria	Bdellovibrio bacteriovorus str. Tiberius	hypothetical protein Bdt_0987 [Bdellovibrio bacteriovorus str. Tiberius]	425859651
AHZ86289.1	-	-	-	481	EP01_15300	bacteria>proteobacteria>deltaproteobacteria	Bdellovibrio bacteriovorus	hypothetical protein EP01_15300 [Bdellovibrio bacteriovorus]	635303550
CAE78974.1	-	-	-	481	Bd1044	bacteria>proteobacteria>deltaproteobacteria	Bdellovibrio bacteriovorus HD100	hypothetical protein predicted by Glimmer/Critica [Bdellovibrio bacteriovorus HD100]	39575133
# 5; CHROMO									
WP_052375710.1	-	-	-	350	-	bacteria>proteobacteria>deltaproteobacteria	Chondromyces apiculatus	hypothetical protein [Chondromyces apiculatus]	918244708
OJY29754.1	-	TUDOR	-	308	BGO98_48945	bacteria>proteobacteria>deltaproteobacteria	Myxococcales bacterium 68-20	hypothetical protein BGO98_48945 [Myxococcales bacterium 68-20]	1113569912
AKV01081.1	-	-	-	278	AKJ09_07744	bacteria>proteobacteria>deltaproteobacteria	Labilithrix luteola	hypothetical protein AKJ09_07744 [Labilithrix luteola]	913618548
AKF10558.1	-	-	-	277	DB32_007707	bacteria>proteobacteria>deltaproteobacteria	Sandaracinus amylolyticus	hypothetical protein DB32_007707 [Sandaracinus amylolyticus]	816961052
WP_053237514.1	-	-	-	277	-	bacteria>proteobacteria>deltaproteobacteria	Sandaracinus amylolyticus	hypothetical protein [Sandaracinus amylolyticus]	921302364
# 5; CHROMO									
ADY52278.1	Agenet	CHROMO	Agenet	236	Pedsa_1721	bacteria>bacteroidetes	Pseudopedobacter saltans DSM 12145	Agenet domain-containing protein [Pseudopedobacter saltans DSM 12145]	324973294
WP_013632769.1	Agenet	CHROMO	Agenet	236	-	bacteria>bacteroidetes	Pseudopedobacter saltans	hypothetical protein [Pseudopedobacter saltans]	503398108
WP_039053802.1	Tudor-knot	TUDOR	Tudor-knot	206	-	bacteria>bacteroidetes	Sphingobacterium sp. T2	hypothetical protein [Sphingobacterium sp. T2]	744500490
ADQ17817.1	Tudor-knot	CHROMO	Tudor-knot	197	Lbys_2121	bacteria>bacteroidetes	Leadbetterella byssophila DSM 17132	Agenet domain-containing protein [Leadbetterella byssophila DSM 17132]	311907376
WP_013408863.1	Tudor-knot	CHROMO	Tudor-knot	197	-	bacteria>bacteroidetes	Leadbetterella byssophila	agenet domain-containing protein [Leadbetterella byssophila]	503174202
# 4; SHD1+CHROMO+BetaPropeller									
CAD73339.1	SHD1+Tudor-knot	CHROMO+BetaPropeller	SHD1+Tudor-knot	742	RB3675	bacteria>planctomycetes	Rhodopirellula baltica SH 1	hypothetical protein-signal peptide and transmembrane prediction [Rhodopirellula baltica SH 1]	32443898
WP_011119489.1	SHD1+Tudor-knot	CHROMO+BetaPropeller	SHD1+Tudor-knot	742	-	bacteria>planctomycetes	Rhodopirellula baltica	hypothetical protein [Rhodopirellula baltica]	499432025
WP_007326763.1	SHD1+Tudor-knot	TUDOR+BetaPropeller	SHD1+Tudor-knot	685	-	bacteria>planctomycetes	Rhodopirellula baltica	hypothetical protein [Rhodopirellula baltica]	494541643
WP_007331135.1	SHD1+Tudor-knot	TUDOR+BetaPropeller	SHD1+Tudor-knot	685	-	bacteria>planctomycetes	Rhodopirellula baltica	hypothetical protein [Rhodopirellula baltica]	494550165
# 4; TUDOR+CAP									
AFY40659.1	CAP	TUDOR+SCP	CAP	321	Lepto7376_4565	bacteria>cyanobacteria	Leptolyngbya sp. PCC 7376	SCP-like extracellular [Leptolyngbya sp. PCC 7376]	427357936
ACA98977.1	CAP	TUDOR+SCP	CAP	320	SYNPCC7002_A0974	bacteria>cyanobacteria	Synechococcus sp. PCC 7002	Pathogenesis-related protein PR-1 precursor [Synechococcus sp. PCC 7002]	169885264
AFY38285.1	CAP	TUDOR+SCP	CAP	320	Lepto7376_1975	bacteria>cyanobacteria	Leptolyngbya sp. PCC 7376	SCP-like extracellular [Leptolyngbya sp. PCC 7376]	427355562
WP_015134051.1	CAP	TUDOR+SCP	CAP	320	-	bacteria>cyanobacteria	Leptolyngbya sp. PCC 7376	hypothetical protein [Leptolyngbya sp. PCC 7376]	504946949
# 4; CHROMO									
WP_024867690.1	Agenet	CHROMO	Agenet	221	-	bacteria>proteobacteria>gammaproteobacteria	Pseudoxanthomonas suwonensis	hypothetical protein [Pseudoxanthomonas suwonensis]	640335743
WP_037060916.1	Agenet	CHROMO	Agenet	221	-	bacteria>proteobacteria>gammaproteobacteria	Pseudoxanthomonas suwonensis	hypothetical protein [Pseudoxanthomonas suwonensis]	739197452
WP_037083140.1	Agenet	CHROMO	Agenet	221	-	bacteria>proteobacteria>gammaproteobacteria	Pseudoxanthomonas sp. J35	hypothetical protein [Pseudoxanthomonas sp. J35]	739219759
WP_049780241.1	SP+Agenet	CHROMO	Agenet	196	-	bacteria>proteobacteria>betaproteobacteria	Aromatoleum aromaticum	hypothetical protein [Aromatoleum aromaticum]	908636219
# 3; ParB+CHROMO+Tox-HNH-DHNNK ; ##### NOT TRUE BACTERIAL #### BACKTRANSFERS FROM EUKARYOTES									
KKU20535.1	DUF262	CHROMO+Tox-HNH-DHNNK	DUF262	606	UX33_C0046G0001	bacteria	Candidatus Azambacteria bacterium GW2011_GWC1_46_13	hypothetical protein UX33_C0046G0001 [Candidatus Azambacteria bacterium GW2011_GWC1_46_13]	818779757
KKU34745.1	DUF262	CHROMO+Tox-HNH-DHNNK	DUF262	606	UX48_C0026G0009	bacteria	Candidatus Azambacteria bacterium GW2011_GWB1_46_27	hypothetical protein UX48_C0026G0009 [Candidatus Azambacteria bacterium GW2011_GWB1_46_27]	818794562
KKU40035.1	DUF262	CHROMO+Tox-HNH-DHNNK	DUF262	606	UX55_C0017G0007	bacteria	Candidatus Azambacteria bacterium GW2011_GWE2_46_45	hypothetical protein UX55_C0017G0007 [Candidatus Azambacteria bacterium GW2011_GWE2_46_45]	818800159
# 3; CHROMO+CarboxypepD-reg-IG									
WP_039682214.1	CarboxypepD_reg	CarboxypepD-reg-IG	CarboxypepD_reg	460	-	bacteria>deinococcus-thermus>deinococci	Deinococcus swuensis	hypothetical protein [Deinococcus swuensis]	746724890
WP_011530581.1	CarboxypepD_reg	CarboxypepD-reg-IG	CarboxypepD_reg	459	-	bacteria>deinococcus-thermus>deinococci	Deinococcus geothermalis	hypothetical protein [Deinococcus geothermalis]	499849847
WP_062159260.1	CarboxypepD_reg	CarboxypepD-reg-IG	CarboxypepD_reg	440	-	bacteria>deinococcus-thermus>deinococci	Deinococcus actinosclerus	hypothetical protein [Deinococcus actinosclerus]	1011240224
# 3; CHROMO+CHROMO									
WP_027462316.1	-	-	-	358	-	bacteria>deinococcus-thermus>deinococci	Deinococcus ficus	hypothetical protein [Deinococcus ficus]	653262516
WP_012695319.1	-	-	-	349	-	bacteria>deinococcus-thermus>deinococci	Deinococcus deserti	hypothetical protein [Deinococcus deserti]	502018198
WP_022802876.1	-	-	-	347	-	bacteria>deinococcus-thermus>deinococci	Deinococcus sp. 2009	hypothetical protein [Deinococcus sp. 2009]	551077781
# 3; CHROMO+CHROMO+CHROMO+CHROMO									
EKE06493.1	SP+DUF4537	TUDOR	DUF4537	287	ACD_18C00347G0018	bacteria	uncultured bacterium	hypothetical protein ACD_18C00347G0018 [uncultured bacterium]	406985762
OGH83645.1	SP+DUF4537	TUDOR	DUF4537	285	A2488_01100	bacteria	Candidatus Magasanikbacteria bacterium RIFOXYC12_FULL_32_21b	hypothetical protein A2488_01100 [Candidatus Magasanikbacteria bacterium RIFOXYC12_FULL_32_21b]	1083697069
OGH91488.1	SP+DUF4537	TUDOR	DUF4537	285	A2507_00785	bacteria	Candidatus Magasanikbacteria bacterium RIFOXYD12_FULL_33_17	hypothetical protein A2507_00785 [Candidatus Magasanikbacteria bacterium RIFOXYD12_FULL_33_17]	1083705646
# 3; CHROMO+CHROMO									
OGH86600.1	-	-	-	187	A2493_02910	bacteria	Candidatus Magasanikbacteria bacterium RIFOXYC12_FULL_33_11	hypothetical protein A2493_02910 [Candidatus Magasanikbacteria bacterium RIFOXYC12_FULL_33_11]	1083700327
EKE07154.1	-	-	-	181	ACD_18C00175G0017	bacteria	uncultured bacterium	hypothetical protein ACD_18C00175G0017 [uncultured bacterium]	406986595
OGH90504.1	-	-	-	181	A2507_03845	bacteria	Candidatus Magasanikbacteria bacterium RIFOXYD12_FULL_33_17	hypothetical protein A2507_03845 [Candidatus Magasanikbacteria bacterium RIFOXYD12_FULL_33_17]	1083704562
# 3; CHROMO									
AKU95838.1	-	-	-	174	AKJ09_02502	bacteria>proteobacteria>deltaproteobacteria	Labilithrix luteola	hypothetical protein AKJ09_02502 [Labilithrix luteola]	913613306
AKT38575.1	-	TUDOR	-	121	CMC5_027220	bacteria>proteobacteria>deltaproteobacteria	Chondromyces crocatus	uncharacterized protein CMC5_027220 [Chondromyces crocatus]	908356000
WP_050430782.1	-	TUDOR	-	121	-	bacteria>proteobacteria>deltaproteobacteria	Chondromyces crocatus	hypothetical protein [Chondromyces crocatus]	913451373
# 3; CHROMO									
ABL79342.1	-	-	-	104	Noca_4756	bacteria>actinobacteria	Nocardioides sp. JS614	hypothetical protein Noca_4756 (plasmid) [Nocardioides sp. JS614]	119525972
ACL42584.1	-	-	-	103	Achl_4633	bacteria>actinobacteria	Pseudarthrobacter chlorophenolicus A6	conserved hypothetical protein (plasmid) [Pseudarthrobacter chlorophenolicus A6]	219862244
BAS18678.1	-	-	-	101	AHiyo8_pI69820	bacteria>actinobacteria	Arthrobacter sp. Hiyo8	hypothetical protein AHiyo8_pI69820 (plasmid) [Arthrobacter sp. Hiyo8]	910749280
# 3; CHROMO									
WP_068491906.1	Agenet	CHROMO	Agenet	103	-	bacteria>proteobacteria>alphaproteobacteria	Magnetospirillum marisnigri	hypothetical protein [Magnetospirillum marisnigri]	1057090973
WP_041041092.1	Agenet	CHROMO	Agenet	101	-	bacteria>proteobacteria>alphaproteobacteria	Magnetospirillum magnetotacticum	hypothetical protein [Magnetospirillum magnetotacticum]	751572227
BAE52057.1	Agenet	CHROMO	Agenet	90	amb3253	bacteria>proteobacteria>alphaproteobacteria	Magnetospirillum magneticum AMB-1	hypothetical protein amb3253 [Magnetospirillum magneticum AMB-1]	82947193
# 3; CHROMO									
SHG29410.1	-	-	-	63	SAMN05443248_1044	bacteria>proteobacteria>alphaproteobacteria	Bradyrhizobium erythrophlei	hypothetical protein SAMN05443248_1044 [Bradyrhizobium erythrophlei]	1109599130
SHH05219.1	-	-	-	63	SAMN05444169_5468	bacteria>proteobacteria>alphaproteobacteria	Bradyrhizobium erythrophlei	hypothetical protein SAMN05444169_5468 [Bradyrhizobium erythrophlei]	1109611862
SHH74951.1	-	-	-	61	SAMN05443248_5984	bacteria>proteobacteria>alphaproteobacteria	Bradyrhizobium erythrophlei	hypothetical protein SAMN05443248_5984 [Bradyrhizobium erythrophlei]	1109603798
# 2; Pepsin+RVT_1+RNaseH+Integrase_Zn+rve+SH3+CHROMO ; ##### NOT TRUE BACTERIAL #### BACKTRANSFERS FROM EUKARYOTES									
OBQ33740.1	RVT_1+rve+Chromo	CHROMO	RVT_1+rve+Chromo	1248	AN485_17910	bacteria>cyanobacteria	Anabaena sp. MDT14b	hypothetical protein AN485_17910, partial [Anabaena sp. MDT14b]	1039191705
OBQ33349.1	RVT_1+rve+Chromo	CHROMO	RVT_1+rve+Chromo	1026	AN485_18965	bacteria>cyanobacteria	Anabaena sp. MDT14b	hypothetical protein AN485_18965 [Anabaena sp. MDT14b]	1039191143
# 2; Integrase_Zn+rve+SH3+CHROMO ; ##### NOT TRUE BACTERIAL #### BACKTRANSFERS FROM EUKARYOTES									
OBQ32496.1	rve+Chromo	CHROMO	rve+Chromo	536	AN485_21335	bacteria>cyanobacteria	Anabaena sp. MDT14b	hypothetical protein AN485_21335 [Anabaena sp. MDT14b]	1039189734
WP_069591915.1	rve+Chromo	CHROMO	rve+Chromo	385	-	bacteria>proteobacteria>gammaproteobacteria	Vibrio cholerae	hypothetical protein [Vibrio cholerae]	1068538606
# 4; SH3+CHROMO									
OBQ32204.1	Chromo	CHROMO	Chromo	203	AN485_22150	bacteria>cyanobacteria	Anabaena sp. MDT14b	hypothetical protein AN485_22150 [Anabaena sp. MDT14b]	1039189178
OBQ31939.1	Chromo	CHROMO	Chromo	172	AN485_22820	bacteria>cyanobacteria	Anabaena sp. MDT14b	hypothetical protein AN485_22820 [Anabaena sp. MDT14b]	1039188625
OBQ27869.1	Chromo	CHROMO	Chromo	166	AN485_24265	bacteria>cyanobacteria	Anabaena sp. MDT14b	hypothetical protein AN485_24265, partial [Anabaena sp. MDT14b]	1039183736
OBQ31967.1	Chromo	CHROMO	Chromo	165	AN485_22735	bacteria>cyanobacteria	Anabaena sp. MDT14b	hypothetical protein AN485_22735 [Anabaena sp. MDT14b]	1039188692
# 2; CHROMO									
ADB16447.1	Agenet	CHROMO	Agenet	549	Psta_1772	bacteria>planctomycetes	Pirellula staleyi DSM 6068	hypothetical protein Psta_1772 [Pirellula staleyi DSM 6068]	283438005
WP_012910710.1	Agenet	CHROMO	Agenet	549	-	bacteria>planctomycetes	Pirellula staleyi	hypothetical protein [Pirellula staleyi]	502674958
# 2; CHROMO									
WP_051246357.1	Tudor-knot	CHROMO	Tudor-knot	456	-	bacteria>proteobacteria>gammaproteobacteria	Thermomonas fusca	hypothetical protein [Thermomonas fusca]	916639266
ODU50900.1	Tudor-knot	CHROMO	Tudor-knot	451	ABS98_07735	bacteria>proteobacteria>gammaproteobacteria	Xanthomonadaceae bacterium SCN 69-48	hypothetical protein ABS98_07735 [Xanthomonadaceae bacterium SCN 69-48]	1064152527
# 2; CHROMO+CHROMO+CHROMO+streptavidin-like_beta-barrel									
AOS44725.1	Agenet	CHROMO+CHROMO	Agenet	437	Verru16b_01792	bacteria>verrucomicrobia	Lacunisphaera limnophila	Agenet domain protein [Lacunisphaera limnophila]	1070533431
WP_069961950.1	Agenet	CHROMO+CHROMO	Agenet	437	-	bacteria>verrucomicrobia	Lacunisphaera limnophila	hypothetical protein [Lacunisphaera limnophila]	1072772081
# 2; CHROMO+CHROMO+CHROMO+CHROMO									
ANE52395.1	SP	TUDOR+CHROMO	-	412	SY85_19820	bacteria>bacteroidetes	Flavisolibacter sp. LCS9	hypothetical protein SY85_19820 [Flavisolibacter sp. LCS9]	1028469475
WP_066406810.1	SP	TUDOR+CHROMO	-	412	-	bacteria>bacteroidetes	Flavisolibacter sp. LCS9	hypothetical protein [Flavisolibacter sp. LCS9]	1054639275
# 2; CHROMO+CHROMO									
AKF02984.1	SP	TUDOR	-	393	DB32_000132	bacteria>proteobacteria>deltaproteobacteria	Sandaracinus amylolyticus	hypothetical protein DB32_000132 [Sandaracinus amylolyticus]	816953478
WP_053230466.1	SP	TUDOR	-	393	-	bacteria>proteobacteria>deltaproteobacteria	Sandaracinus amylolyticus	hypothetical protein [Sandaracinus amylolyticus]	921295081
# 2; CHROMO+CHROMO									
KGE70990.1	Tudor-knot	CHROMO	Tudor-knot	328	DC28_13770	bacteria>spirochaetes	Spirochaeta lutea	hypothetical protein DC28_13770 [Spirochaeta lutea]	689723624
WP_037549678.1	Tudor-knot	CHROMO	Tudor-knot	325	-	bacteria>spirochaetes	Spirochaeta lutea	hypothetical protein [Spirochaeta lutea]	739695259
# 2; TPR+CHROMO+CHROMO									
WP_054040137.1	TPR_19+SP+Tudor-knot	CHROMO+CHROMO	TPR_19+Tudor-knot	323	-	bacteria	bacterium 336/3	hypothetical protein [bacterium 336/3]	929016206
WP_054040138.1	-	CHROMO	-	235	-	bacteria	bacterium 336/3	hypothetical protein [bacterium 336/3]	929016207
# 2; CHROMO+CHROMO									
ACO45037.1	-	CHROMO	-	320	Deide_02280	bacteria>deinococcus-thermus>deinococci	Deinococcus deserti VCD115	Conserved hypothetical protein, precursor [Deinococcus deserti VCD115]	226317041
WP_012692160.1	-	CHROMO	-	320	-	bacteria>deinococcus-thermus>deinococci	Deinococcus deserti	hypothetical protein [Deinococcus deserti]	502012201
# 2; Band_7+CHROMO									
ACY17932.1	-	-	-	279	Hoch_5449	bacteria>proteobacteria>deltaproteobacteria	Haliangium ochraceum DSM 14365	hypothetical protein Hoch_5449 [Haliangium ochraceum DSM 14365]	262081963
WP_012830524.1	-	-	-	279	-	bacteria>proteobacteria>deltaproteobacteria	Haliangium ochraceum	hypothetical protein [Haliangium ochraceum]	502592944
# 2; CHROMO+CHROMO									
WP_022802883.1	MBT	CHROMO	MBT	275	-	bacteria>deinococcus-thermus>deinococci	Deinococcus sp. 2009	hypothetical protein [Deinococcus sp. 2009]	551077795
WP_027462321.1	MBT	CHROMO	MBT	275	-	bacteria>deinococcus-thermus>deinococci	Deinococcus ficus	hypothetical protein [Deinococcus ficus]	653262546
# 2; CHROMO+CHROMO									
WP_007617925.1	SP	TUDOR+CHROMO	-	263	-	bacteria>proteobacteria>gammaproteobacteria	Paraglaciecola arctica	hypothetical protein [Paraglaciecola arctica]	494891879
EPJ52922.1	SP	TUDOR+CHROMO	-	259	OFPI_12340	bacteria>proteobacteria>gammaproteobacteria	Osedax symbiont Rs2	hypothetical protein OFPI_12340 [Osedax symbiont Rs2]	515422790
# 2; CHROMO+CHROMO									
AKF05714.1	SP+Tudor-knot	CHROMO+CHROMO	Tudor-knot	221	DB32_002863	bacteria>proteobacteria>deltaproteobacteria	Sandaracinus amylolyticus	hypothetical protein DB32_002863 [Sandaracinus amylolyticus]	816956208
WP_053232954.1	SP+Tudor-knot	CHROMO+CHROMO	Tudor-knot	221	-	bacteria>proteobacteria>deltaproteobacteria	Sandaracinus amylolyticus	hypothetical protein [Sandaracinus amylolyticus]	921297701
# 2; CHROMO									
ADB18036.1	SP+SP+SP+SP	-	-	210	Psta_3372	bacteria>planctomycetes	Pirellula staleyi DSM 6068	hypothetical protein Psta_3372 [Pirellula staleyi DSM 6068]	283439594
WP_012912298.1	SP+SP+SP+SP	-	-	210	-	bacteria>planctomycetes	Pirellula staleyi	hypothetical protein [Pirellula staleyi]	502676569
# 2; CHROMO+CHROMO									
WP_044890980.1	-	-	-	194	-	bacteria>proteobacteria>deltaproteobacteria	Myxococcus hansupus	hypothetical protein [Myxococcus hansupus]	768721814
AKQ68284.1	-	-	-	188	A176_005196	bacteria>proteobacteria>deltaproteobacteria	Myxococcus hansupus	hypothetical protein A176_005196 [Myxococcus hansupus]	891163599
# 2; CHROMO									
WP_041660441.1	SP+Tudor-knot	CHROMO	Tudor-knot	112	-	bacteria>cyanobacteria	Acaryochloris marina	hypothetical protein [Acaryochloris marina]	753955539
ABW30353.1	Tudor-knot	CHROMO	Tudor-knot	99	AM1_5397	bacteria>cyanobacteria	Acaryochloris marina MBIC11017	hypothetical protein AM1_5397 [Acaryochloris marina MBIC11017]	158308736
# 1; CHROMO+CASPASE									
APR79246.1	-	TUDOR+CASPASE	-	480	A7982_04593	bacteria>proteobacteria>deltaproteobacteria	Minicystis rosea	Hypothetical protein A7982_04593 [Minicystis rosea]	1127357843
# 1; CHROMO+SHD1+CHROMO+BetaPropeller									
CAD77645.1	SHD1	-	SHD1	801	RB12538	bacteria>planctomycetes	Rhodopirellula baltica SH 1	hypothetical protein-signal peptide and transmembrane prediction [Rhodopirellula baltica SH 1]	32448128
# 1; SHD1+CHROMO									
KLU04478.1	SHD1+Tudor-knot	CHROMO	SHD1+Tudor-knot	290	RISK_003532	bacteria>planctomycetes	Rhodopirellula islandica	putative signal peptide and transmembrane protein [Rhodopirellula islandica]	831420543
# 1; ZNR+CHROMO									
OJY25264.1	PAT1	ZNR+TUDOR	PAT1	409	BGO98_03845	bacteria>proteobacteria>deltaproteobacteria	Myxococcales bacterium 68-20	hypothetical protein BGO98_03845 [Myxococcales bacterium 68-20]	1113565354
# 1; Beta-lactamase+CHROMO									
WP_051341443.1	SP+Beta-lactamase+Agenet	CHROMO	Beta-lactamase+Agenet	430	-	bacteria>proteobacteria>alphaproteobacteria	Azospirillum halopraeferens	hypothetical protein [Azospirillum halopraeferens]	916734387
# 1; Peptidase+CHROMO									
OGQ47073.1	SP+Peptidase_M48+Tudor-knot	CHROMO	Peptidase_M48+Tudor-knot	384	A3H42_01250	bacteria>proteobacteria>deltaproteobacteria	Deltaproteobacteria bacterium RIFCSPLOWO2_02_FULL_46_8	hypothetical protein A3H42_01250 [Deltaproteobacteria bacterium RIFCSPLOWO2_02_FULL_46_8]	1084811295
# 1; ANK+CHROMO									
WP_052351844.1	Ank_2	ANK	Ank_2	248	-	bacteria>deinococcus-thermus>deinococci	Deinococcus pimensis	hypothetical protein [Deinococcus pimensis]	918218712
# 1;									
APR77355.1	-	CHROMO+CHROMO+CHROMO	-	387	A7982_02702	bacteria>proteobacteria>deltaproteobacteria	Minicystis rosea	Hypothetical protein A7982_02702 [Minicystis rosea]	1127355952
OGL96415.1	LBR_tudor	CHROMO+TUDOR	LBR_tudor	298	A2318_03400	bacteria	Candidatus Uhrbacteria bacterium RIFOXYB2_FULL_45_11	hypothetical protein A2318_03400 [Candidatus Uhrbacteria bacterium RIFOXYB2_FULL_45_11]	1084204816
WP_017717369.1	DUF4537+DUF4537	TUDOR+TUDOR	DUF4537+DUF4537	218	-	bacteria>cyanobacteria	Oscillatoria sp. PCC 10802	hypothetical protein [Oscillatoria sp. PCC 10802]	516326710
OFX28594.1	DUF4537	TUDOR+CHROMO	DUF4537	131	A2X08_16175	bacteria>bacteroidetes	Bacteroidetes bacterium GWA2_32_17	hypothetical protein A2X08_16175 [Bacteroidetes bacterium GWA2_32_17]	1082370077
WP_007679426.1	SP	CHROMO+TUDOR	-	450	-	bacteria>proteobacteria>alphaproteobacteria	alpha proteobacterium BAL199	hypothetical protein [alpha proteobacterium BAL199]	494953398
OJJ25920.1	Tudor-knot+Tudor-knot	CHROMO	Tudor-knot+Tudor-knot	297	BI308_09315	bacteria>cyanobacteria	Roseofilum reptotaenium AO1-A	hypothetical protein BI308_09315 [Roseofilum reptotaenium AO1-A]	1111739498
APZ91266.1	Ribosomal_L12	-	Ribosomal_L12	573	rplL_1	bacteria>planctomycetes	Fuerstia marisgermanicae	50S ribosomal protein L7/L12 [Fuerstia marisgermanicae]	1140106709
WP_026607406.1	-	CHROMO	-	501	-	bacteria>proteobacteria>alphaproteobacteria	Methylocapsa acidiphila	hypothetical protein [Methylocapsa acidiphila]	651613277
AMV19356.1	SP+SP+SP	CHROMO	-	410	VT03_15800	bacteria>planctomycetes	Planctomyces sp. SH-PL14	hypothetical protein VT03_15800 [Planctomyces sp. SH-PL14]	1015437571
WP_054038141.1	Agenet	CHROMO	Agenet	391	-	bacteria	bacterium 336/3	hypothetical protein [bacterium 336/3]	929014210
EKD72297.1	-	CHROMO	-	321	ACD_45C00712G0001	bacteria	uncultured bacterium	hypothetical protein ACD_45C00712G0001 [uncultured bacterium]	406939234
OHD36272.1	Agenet	CHROMO	Agenet	301	A2Y30_16580	bacteria>spirochaetes	Spirochaetes bacterium GWE1_32_154	hypothetical protein A2Y30_16580 [Spirochaetes bacterium GWE1_32_154]	1088506031
SDA38664.1	-	TUDOR	-	239	SAMN02910368_00191	bacteria>firmicutes	Lachnospiraceae bacterium G11	hypothetical protein SAMN02910368_00191 [Lachnospiraceae bacterium G11]	1086294275
WP_043118959.1	Chromo	CHROMO	Chromo	225	-	bacteria>proteobacteria>gammaproteobacteria	Solemya velum gill symbiont	hypothetical protein [Solemya velum gill symbiont]	759394267
AIE84434.1	SP	TUDOR	-	210	OP10G_1066	bacteria>armatimonadetes	Fimbriimonas ginsengisoli Gsoil 348	hypothetical protein OP10G_1066 [Fimbriimonas ginsengisoli Gsoil 348]	663072279
OHD82823.1	53-BP1_Tudor	TUDOR	53-BP1_Tudor	209	A3J97_15250	bacteria>spirochaetes	Spirochaetes bacterium RIFOXYC1_FULL_54_7	hypothetical protein A3J97_15250 [Spirochaetes bacterium RIFOXYC1_FULL_54_7]	1088556041
WP_041764146.1	-	TUDOR	-	209	-	bacteria>cyanobacteria	Leptolyngbya sp. PCC 7376	hypothetical protein [Leptolyngbya sp. PCC 7376]	754132614
AFM11971.1	-	-	-	208	Turpa_1323	bacteria>spirochaetes	Turneriella parva DSM 21527	hypothetical protein Turpa_1323 [Turneriella parva DSM 21527]	390610819

WP_072833409.1	Tudor-knot	CHROMO	Tudor-knot	194	-	bacteria>bacteroidetes	Flavisolibacter ginsengisoli	hypothetical protein [Flavisolibacter ginsengisoli]	1119952611
OHD82840.1	Tudor-knot	CHROMO	Tudor-knot	183	A3J97_08950	bacteria>spirochaetes	Spirochaetes bacterium RIFOXYC1_FULL_54_7	hypothetical protein A3J97_08950 [Spirochaetes bacterium RIFOXYC1_FULL_54_7]	1088556061
OGL88706.1	SP+DUF4537	TUDOR	DUF4537	150	A3H75_00500	bacteria	Candidatus Uhrbacteria bacterium RIFCSPLOWO2_02_FULL_51_9	hypothetical protein A3H75_00500 [Candidatus Uhrbacteria bacterium RIFCSPLOWO2_02_FULL_51_9]	1084196583
WP_027893043.1	SP	-	-	146	-	bacteria>deinococcus-thermus>deinococci	Meiothermus chliarophilus	hypothetical protein [Meiothermus chliarophilus]	654421787
WP_013386960.1	-	-	-	136	-	bacteria>fusobacteria	Ilyobacter polytropus	hypothetical protein [Ilyobacter polytropus]	503152299
KXK41781.1	-	CHROMO	-	90	UZ11_BCD004001754	bacteria>bacteroidetes	Bacteroidetes bacterium OLB11	hypothetical protein UZ11_BCD004001754 [Bacteroidetes bacterium OLB11]	1000279860
KKW20987.1	-	-	-	72	UY62_C0036G0009	bacteria	Parcubacteria group bacterium GW2011_GWF2_50_9	hypothetical protein UY62_C0036G0009 [Parcubacteria group bacterium GW2011_GWF2_50_9]	818885757
WP_043553750.1	Chromo	CHROMO	Chromo	71	-	bacteria>proteobacteria>alphaproteobacteria	Acetobacter malorum	hypothetical protein [Acetobacter malorum]	759863213
OHD37675.1	-	TUDOR	-	68	A2Y30_10990	bacteria>spirochaetes	Spirochaetes bacterium GWE1_32_154	hypothetical protein A2Y30_10990 [Spirochaetes bacterium GWE1_32_154]	1088507481

Back to Contents

- **Supplementary S4: Bacterial chromo domains: multiple sequence alignment of representative sequences**

```
FINAL                      ------EEEEEE--------EEEEEEEEEE------EEEEEEEE---E-----EE----EEEE-----
WP_068362540.1_1   44   -ACPINMAVDIEWN--GG--QFEGVILDGPD--PQGNCLVSYDG---WDAS-WNE----WVDIGRLAP    96
WP_068362540.1_2  106   PTCPVGASREIEWN--GS--LWDGTILEGPN--AAGECYVVTYEG---WDAS-WNE----WVAQERIAP   159
WP_068362540.1_3  165   TECPVGIPLEIQWN--NS--WWSGVVLEGPN--AQGQCFVTYEG---WDAS-WNE----WVTVDRLRA   218
APR83931.1_1       61   PKYGVGDHVLVQWE--GN--EYPAHILEAQ---GPAKFKVHYDG---YADV-WDE----VVPKDRIKS   113
APR83931.1_2      139   NRYKINDRVRVEWH--GQ--IYSATITGIV---GQEQYRVRYDG---YGPE-WDE----IVGLARIQP   191
KXK45002.1_1       50   -AQKVNDKVQINSN--GS--WYPGKILKVNS--EEKTYFVTYDG---WGDS-WNE----WVTVDRLKD   102
KXK45002.1_2      113   TKFKVGDKVEVEYGMIPE----PATIIEV----GENKYHIQ------FDKKAFGDK---WVAEGAIKK   163
WP_010472205.1_1   41   AACAVGQRGEVLWK--GK--WYPAQVLDT----SGRQCYITYDG---YSSS-WDE----WVGPNRFRP    92
WP_010472205.1_2  100   AAYRVGQSVQILWK--GK--WYPGRVLDV----SGNRYFITYDG---YSSS-WNE----WVGPARLSR   151
WP_049783751.1     41   AKCVVGQRGEVLWK--GK--WYPAQVLDT----SGRQCYITYDG---YSSS-WDE----WVGPNRFRP    92
WP_077119839.1     22   SAQTKGDWVLGNYKGAGY--WFPGVVEKL----AGDKITIR------YDDG-DRE----TVSINAVRP    72
WP_045778338.1     92   -PYRLGDAVMVLWK--GK--WYASTVIQL----GKNKVKIHYDN---YDSS-WDE----WVEAARIRY   142
KFB72044.1_1       23   ALCSVGDRAQVSWK--GQ--WYPAKVTKVNE--DQTKCFIRYDG---YGSE-WDE----WVGSDRIKV    76
KFB72044.1_2       82   PGFQVGDSVQVKWK--GE--WYPASVIST----KPDMWKIHYDG---YAES-WDE----WVERDRIRH   133
WP_027844674.1_1   31   PICTRGQKAEAKWK--GK--WYPVKVLDV----SGQKCFITYEG---YDSS-WNE----WIGSRRFRA    82
WP_027844674.1_2   82   AYFRTGSSVNVRWK--GK--WYPARILQV----SGNGYKITYDG---YDSS-WDE----WVEPARVSR   133
WP_041739350.1_1   29   SPCSVGQKAEVLWK--GK--WYPAEVLNV----KNNKCYVTYEG---YDSS-WNE----WVEARRFRA    80
WP_041739350.1_2   80   ASFKTGDSVRIFWK--GK--WYPGKILNV----GKNSYKVTYTG---YDSS-WDE----SVEPARVSR   131
AHZ86290.1_1      205   TTVVVGDRVLFDNG--TS---YVGTVKEIF---MSGKARVSFPG---YSQD-S------ILDLKDLAR   254
AHZ86290.1_2      260   ATQCVGDRVLFDNG--NQ---YVGMISMVF---ANGVRQVKFPG---YSEP-S------FIKSTKLTK   309
AHZ86290.1_3      315   GSVCVGERIIFSNG--SD--YYAGTVRETF---VNGMVSIKFDG---YNEL-S------FLDHKKLMK   365
AHZ86290.1_4      371   SDICVDTRVMFDNG--SG--NYLGRVTEIY---ADGTAKIKFEG---YSEY-S------FIKTSRLVK   421
AHZ86290.1_5      427   KGYSGGTRVLFNNG--SG--YYVGTVREVF---ADGTASIRFEG---YSDL-S------FIKVDRLGR   477
AHZ86290.1_6      483   KNICTGNRVIL--N--GQ---YSGTVRAVY---SNGYALVKYDG---YTDL-T------YTDVSKLSR   530
SFT96502.1_1       27   SAQTAGDWVLGNYKNAGY--WFPGIAEKV----EGGTVTIR------YDDG-DRE----TVSLDKVRP    77
SFT96502.1_2       79   -DWMIGMKVQCNYKNAGE--WYPGTIASL----AGEKIGIN------YDDG-DKE----TTKTGRCRT   128
WP_026817379.1_1   73   -HWEAGSTLQCNWRNRGR--YYPGVVERI----DGERIEFR------YDDG-DRE----TMTISRCRS   122
WP_026817379.1_2   21   SAQAPGDRVLALWPADGM--WYPARVEAR----AGDEVQVA------YDDG-DVG----TVRADQFVP    71
WP_054134435.1_1   58   -AQEVGDWVLSPWR--GSSVFYPGVVESR----SGSVITVR------FDDG-DVE----TRQADTVVP   107
WP_054134435.1_2  109   -DWQAGSRISCAWSD-GK--WYKATIRSIAA--DGYTMQIR------YDDDGTVE----NTNTGRCRT   160
AFZ02755.1_1      331   NKYTVGQTIEVWNSEEED--WYRATIEKA----SEKRFFVNYIG---KGSS-NDE----WVEEDNIRI   384
AFZ02755.1_2      393   NGYAIGNKVKVWDEENED--WYSAIIGKT----QGNQYFVHYVD---YDSS-YDE----WLDLEEIS-   445
AFY43036.1_1      325   TQYEVNSTVEVWDEEQEE--WYKATIQKI----KDDQFFINYVG---YGSS-ADE----WVGEDVIRI   378
AFY43036.1_2      387   NGFAVGQKVKCWDEENEE--WYSATIEEI----RGQQYFVHYQG---YDSS-YDE----WLDLAEIR-   439
WP_054466600.1_1  320   GEFKVGQLVDVLSE--DY--WYAGTVREL----QGNLCLIRYLYN--NDPS-DDE----WIAQTNLAP   372
WP_054466600.1_2  375   SNYEVGDEIEVLYSD-GE--WYEAEIKKI----KGDTYLVEYTDENFYDDE-DDEEETEWVAWQRICC   434
WP_023067754.1    320   SRFSVGETVEVWDKDEGE--WYESTIQDIKVKKEKTKYFVHYIG---YDSD-EDE----WVKEKNIRS   377
WP_046279647.1    320   SRFSVGETVEVWDKDEGE--WYEATIKEIRIKKEKNQYFVHYVG---YDSD-EDE----WVKEKNIRS   377
ADG68645.1_1      280   TDPEIGQRTEIEWH--GK--YYRGFVIGR----NGALSRVHYYG---YQES-DDE----WVTPERLRT   331
ADG68645.1_2      336   -TRPINSPVEIWWK--SE--WYRGRIIGV----KHGLHLVKYDK---WGPE-WNE----WVTIHRLRD   386
WP_068848381.1    273   TDPEVGQRAEIEWH--GK--YYRGFVIGR----NGALSRVHYYG---YQES-DDE----WVTPERLRI   324
WP_064196371.1_1  251   KNSRIGERVQVDYD--GT--DWLGFIENVE---ADKRLKVRFYS---YTNN-EVD----VVEASRVKA   303
WP_064196371.1_2  308   KDFPIGARVKVYST--NDKAWYAAKVLKK----FSCLHYIHYEG---YDDE-YDE----WVSTDKIKK   361
WP_012694986.1     92   RAISFKGLDEEEDE--ES--YWLSFLLTE----KQTRYVGVL-----YGDN-ESV----VLGWCQLKA   141
ACO46634.1         92   RGLTYKQLAEEEDD--ES--YSLSFLLTE----KSNRYVAVL-----YGDA-ESV----VLGWCQLKG   141
CCG08965.1        170   GLCHPGRRVEVLWE--GD--WYPARVLEET---GPGRCYIHYEG---YGSD-DDE----EVSPRRMRP   222
WP_061629242.1     61   SARKVGDRVEVEWK--GA--YKPAIITGVV---APGSYRVHFEG---YDAQ-WDE----VVTEARIKG   113
AFY00688.1        250   GTHCVGDRVLFDNG--NQ---YVGVISLVF---SNGVRQVKFPG---YSEP-S------YLKGQDLQK   299
WP_049780241.1     30   ALCVKGRAVSVLYG--GD--WYPAKVLDGPD--RMSTCLVSYDG---YGSN-WDE----WVSAKRMRP    83
KXK41781.1         18   KSQNIKEIVQINHN--AT--WYEGKILKF----ENGSFYVSYYV---WSHS-WNE----WGNKDKLKG    69
consensus/70%           s.hplGpplbh..p..up..aY.uplbp......ssphblpa.s...Ysss..sE....hlp.p+lp.
```

Back to Contents

- **Supplementary Figure S1. The C-terminal zinc ribbon of nicotinate phosphoribosyltransferase.**

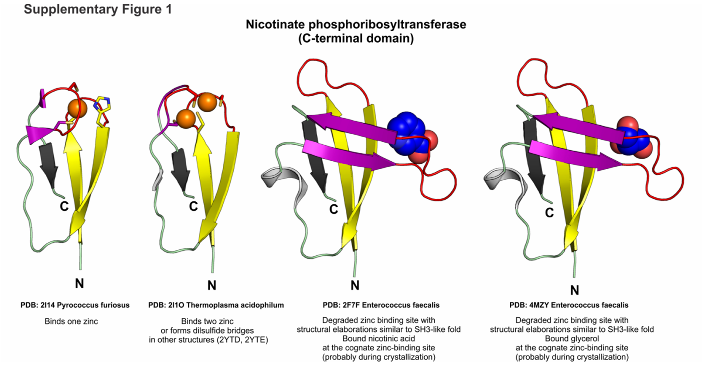

Coloring scheme follows that used in Figure 1(A-H). The zinc ions and chemical moieties bound in the open end of the barrels are shown as spheres.

Back to Contents
